# Overcoming Multidrug Resistance (MDR): Design, Biological Evaluation and Molecular Modelling Studies of 2,4‐Substituted Quinazoline Derivatives

**DOI:** 10.1002/cmdc.202200027

**Published:** 2022-05-12

**Authors:** Laura Braconi, Elisabetta Teodori, Marialessandra Contino, Chiara Riganti, Gianluca Bartolucci, Dina Manetti, Maria Novella Romanelli, Maria Grazia Perrone, Nicola Antonio Colabufo, Stefano Guglielmo, Silvia Dei

**Affiliations:** ^1^ Department of Neuroscience, Psychology, Drug Research and Child Health Section of Pharmaceutical and Nutraceutical Sciences University of Florence via Ugo Schiff 6 50019 Sesto Fiorentino Italy; ^2^ Department of Pharmacy – Drug Sciences University of Bari “A. Moro” via Orabona 4 70125 Bari Italy; ^3^ Department of Oncology University of Turin Via Santena 5/bis 10126 Torino Italy; ^4^ Department of Drug Science and Technology University of Turin Via P. Giuria 9 10125 Torino Italy

**Keywords:** multidrug resistance reversers, efflux transporters modulators, molecular docking studies, membrane proteins, structure-activity relationships

## Abstract

Some 2,4‐disubstituted quinazolines were synthesized and studied as multidrug resistance (MDR) reversers. The new derivatives carried the quinazoline‐4‐amine scaffold found in modulators of the ABC transporters involved in MDR, as the TKIs gefitinib and erlotinib. Their behaviour on the three ABC transporters, P‐gp, MRP1 and BCRP, was investigated. Almost all compounds inhibited the P‐gp activity in MDCK‐MDR1 cells overexpressing P‐gp, showing EC_50_ values in the nanomolar range (**1 d**, **1 e**, **2 a**, **2 c**, **2 e**). Some compounds were active also towards MRP1 and/or BCRP. Docking results obtained by in silico studies on the P‐gp crystal structure highlighted common features for the most potent compounds. The P‐gp selective compound **1 e** was able to increase the doxorubicin uptake in HT29/DX cells and to restore its antineoplastic activity in resistant cancer cells in the same extent of sensitive cells. Compound **2 a** displayed a dual inhibitory effect showing good activities towards both P‐gp and BCRP.

## Graphical abstract

**2,4‐disubstituted quinazoline derivatives as MDR reversers**: Derivatives carrying the quinazoline‐4‐amine scaffold were studied for their activity on ABC transporters, P‐gp, MRP1 and BCRP, involved in multidrug resistance (MDR). Potent P‐gp inhibitors with activity in the nanomolar range were identified. The results allowed us to propose structural requirements for defining P‐gp, MRP1 and BCRP activity and selectivity.

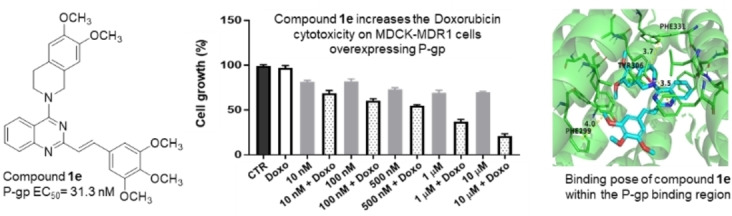

## Introduction

Chemotherapeutic treatments are the most important methods to eradicate malignant tumours. However, the success of chemotherapy is often impaired by the resistance that tumour cells develop to the anticancer drugs during clinical treatments. One of the main mechanisms involved in this phenomenon is multidrug resistance (MDR), a type of acquired cross resistance to a variety of unrelated anticancer drugs after exposure to even a single chemotherapeutic agent.^[1]^ MDR is due to complex and multifactorial mechanisms, among which the overexpression of efflux pumps plays a crucial role in the progress of chemoresistance in cancer.[^2^, ^3^] The main efflux pumps responsible for MDR belong to the ATP‐binding cassette (ABC) protein family. These proteins are ATP dependent transporters that extrude chemotherapeutic agents reducing their intracellular concentration; consequently, the effectiveness of anticancer drugs is weakened.^[4]^ In humans, the transporter proteins mainly associated with MDR are P‐glycoprotein (P‐gp, ABCB1), Multidrug‐Resistance‐associated Protein‐1 (MRP1, ABCC1), and Breast Cancer Resistance Protein (BCRP, ABCG2) which are overexpressed in several resistant tumours.[^5^, ^6^]

P‐glycoprotein is the most studied and important ABC transporter and it was considered responsible for the efflux of chemotherapeutic agents from many cancer cells leading to their drug insensitivity.[^7^, ^8^] Recent studies have also shown that P‐gp overexpression was associated with a more aggressive tumour phenotype promoting tumour invasion and metastasis.^[9]^ MRP1 and BCRP were also associated with reduced tumour responses to cytotoxic drugs. These three ABC transporters can be co‐expressed in many resistant cancer cells.^[10]^ In particular, P‐gp and BCRP are co‐overexpressed in many solid tumours and cancer stem cells causing their insensitivity to chemotherapeutic agents and consequently the failure of many long‐lasting chemotherapeutic treatments.[^11^, ^12^, ^13^]

In consideration of the relationship between multidrug resistance and ABC transporters, an appropriate strategy to circumvent MDR is the co‐administration of anticancer drugs that are substrates of the efflux pumps, with an inhibitor/substrate of these transporter proteins improving both the chemotherapeutic response and the patient outcomes. For this reason, many efforts have been made to identify modulators of these three proteins in the last years.[^14^, ^15^, ^16^] Several of these compounds, also known as chemosensitizers, have been studied in clinical trials carried out in several different cancer types. However, no substantial survival benefits have been established. The observed drawbacks are mainly due to lack of significant clinical efficacy, pharmacokinetic interactions, adverse effects and toxicity.[^17^, ^18^, ^19^]

Therefore, the discovery of novel potent and efficacious ABC transporter modulators is still a key issue to overcome some of the obstacles to the use of chemosensitizers in MDR reversing.

Due to the co‐expression of P‐gp, MRP1 and BCRP in many tumours and to their overlapping specificity, exhibited towards a variety of substrates,^[20]^ the selective inhibition of one efflux transporter could be compensated by the remaining transporters. Therefore, in the last years, many efforts have been devoted to identifying new derivatives able to simultaneously modulate the activity of different transporters.

Compounds able to modulate the efflux activity of ABC transporters bear variable scaffolds and are characterized by different chemical structures. Nevertheless, some specific physicochemical features needed to bind these proteins, in particular for P‐gp and BCRP, were identified, such as the ability to establish hydrogen bond interactions, the presence of aromatic rings, high lipophilicity and one or more protonable nitrogen atoms.^[21]^

**Figure 1:**
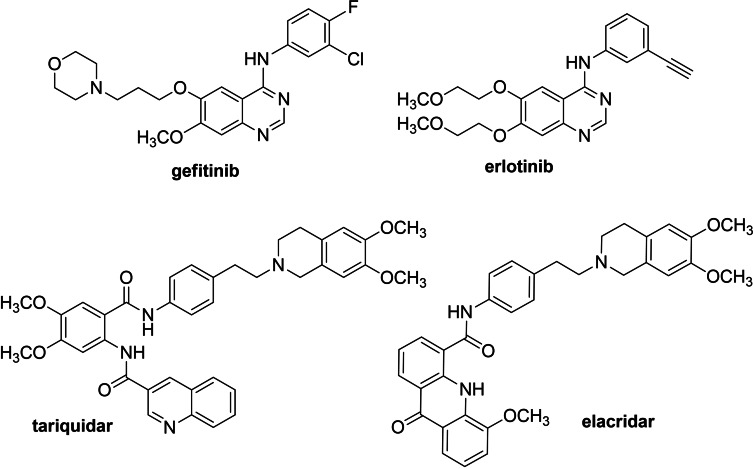
TKIs with P‐gp and BCRP inhibitory activity, gefitinib and erlotinib, and third generation P‐gp inhibitors, tariquidar and elacridar.

Recently, quinazoline‐4‐amine based tyrosine kinase inhibitors (TKIs), such as gefitinib and erlotinib, have been identified as ABC transporters modulators (Figure 1),[^22^, ^23^, ^24^] and many studies reported on compounds with the 4‐anilino‐quinazoline scaffold as potent BCRP inhibitors.[^25^, ^26^, ^27^] In addition, the quinazoline moiety was introduced into compounds that proved to be P‐gp inhibitors.^[28]^

**Figure 2:**
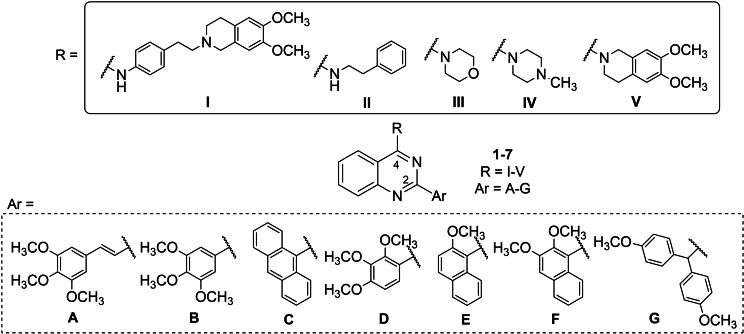
General structure of derivatives **1**–**7**.

In the present study, we designed and synthesized a new series of 2,4‐disubstituted quinazoline derivatives with the aim of discovering new P‐gp and/or BCRP inhibitors (Figure 2). For this purpose, secondary or tertiary protonable amines were inserted in position 4 of the quinazoline scaffold in place of the aniline residues typical of TKIs. The selected amines were 4‐(2‐(6,7‐dimethoxy‐3,4‐dihydroisoquinolin‐2(1*H*)‐yl)ethyl)aniline (**I**), 2‐phenylethan‐1‐amine (**II**), morpholine (**III**), 1‐methylpiperazine (**IV**) and 6,7‐dimethoxy‐1,2,3,4‐tetrahydroisoquinoline (**V**) (Figure 2). The amine **I** was chosen since it is present in two of the most interesting third generation P‐gp inhibitors tariquidar and elacridar that are also able to bind the BCRP transporter (Figure 1).^[29]^ The other amines (**II–V**) were chosen to vary both the steric hindrance and the electronic proprieties. Position 2 of the quinazoline nucleus was substituted with aryl residues able to improve the P‐gp interaction. Therefore, aromatic groups, such as anthracene or methoxy‐substituted aryl moieties (Figure 2), were chosen because of their presence in our previously synthesized compounds, with different scaffolds, that have proved to be potent and efficacious P‐gp dependent MDR reversers.[^30^, ^31^] In particular, the hydrogen bond acceptor methoxy group is considered important for the MDR‐reversing activity and is present in many well‐known P‐gp modulators.^[29]^

These new compounds were evaluated for their P‐gp interaction profile and selectivity towards the two other ABC transporters, MRP1 and BCRP. For these studies, Madin‐Darby Canine Kidney (MDCK) transfected cells (MDCK‐MDR1, MDCK‐MRP1 and MDCK‐BCRP cells overexpressing P‐gp, MRP1 and BCRP, respectively) were used. Molecular docking simulation studies were performed in order to identify the binding mode of these compounds within the P‐gp binding pocket. One of the best compounds was further tested alone and in co‐administration with the antineoplastic drug doxorubicin in a pure model, MDCK‐MDR1 cells, and in a model of acquired resistance to doxorubicin, HT29/DX cells.^[32]^

## Results and Discussion

### Chemistry

**Scheme 1:**
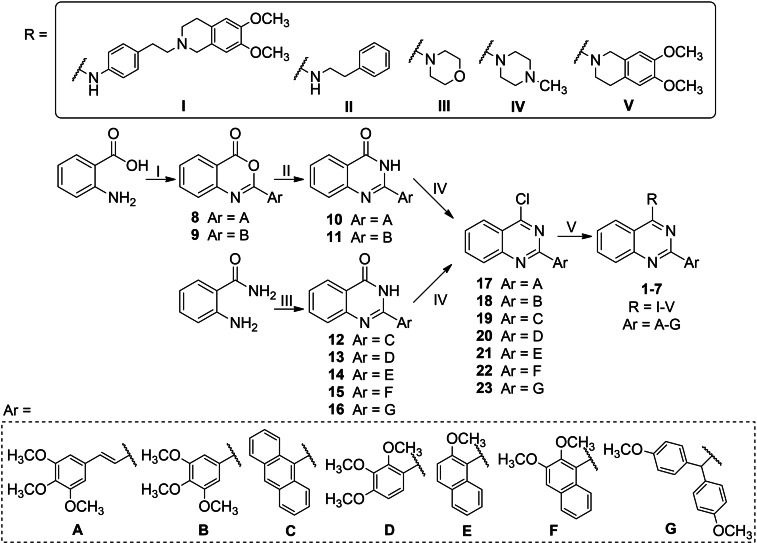
*Reagents and conditions*: I) ArCOCl, dry pyridine, rt, 4 h; II) NH_4_OH (33.0 %), EtOH, 80 °C, 20 h; III) ArCHO, CuCl_2_, EtOH, reflux, 16 h, *or* ArCOOH, HATU, DIPEA, dry CH_2_Cl_2_, 50 °C, 16 h, then NaOH (10.0 M), EtOH, rt, 2 h; IV) SOCl_2_, dry DMF, CHCl_3_ (free of ethanol), 50 °C, 6 h *or* POCl_3_, reflux, 5–12 h; V) amines, CH_3_SO_3_H, abs. EtOH, reflux, 4 h (Method A), *or* amines, K_2_CO_3_, dry DMF, 60 °C, 5 h (Method B). For the structure of final compounds **1–7** see Table 1.

The key intermediates needed to achieve final compounds **1**–**7** were the 4‐chloroquinazolines **17**–**23**, which were synthesized by reaction of the proper quinazolin‐4(3*H*)‐one **10**–**16** with SOCl_2_ in CHCl_3_ (free of ethanol)^[28]^ or POCl_3_ ^[27]^ (Scheme 1). Quinazolin‐4(3*H*)‐ones **10**–**16** were obtained following three different procedures (Scheme 1). On one hand, the commercially available 2‐aminobenzoic acid was reacted with freshly prepared acyl chlorides in dry pyridine, affording the intermediates **8** and **9**, which were treated with ammonia water in ethanol, to obtain the quinazolin‐4(3*H*)‐ones **10** and **11** (Ar=**A**, **B**).^[28]^ On the other hand, **12**–**15** (Ar=**C**‐**F**) were synthesized by reaction of the commercially available anthranilamide, the proper aldehyde and CuCl_2_ in ethanol, with very good yields.^[33]^ Otherwise, the quinazolin‐4(3*H*)‐one **16** (Ar=**G**) was synthesized, following the procedure reported in ref. 34, through a coupling reaction between anthranilamide and 2,2‐bis(4‐methoxyphenyl)acetic acid,^[35]^ by using HATU as the activating agent, in the presence of DIPEA. Intermediates **9**, **11** and **18** were already reported in ref. 28, while **10**,^[36]^ **12**,^[37]^ **13**,^[38]^ and **17** ^[36]^ had been already described but were obtained in different ways.

Finally, to synthesize compounds **1**–**7** the proper 4‐chloroquinazoline (**17**–**23**) was reacted with the suitable amine in the presence of methanesulfonic acid in abs. ethanol (Method A), or in the presence of K_2_CO_3_ in dry DMF (Method B) (Scheme 1). Most of the used amines are commercially available (2‐phenylethanamine, morpholine, 1‐methylpiperazine and 6,7‐dimethoxy‐1,2,3,4‐tetrahydroisoquinoline) while 4‐(2‐(6,7‐dimethoxy‐3,4‐dihydroisoquinolin‐2(1*H*)‐yl)ethyl)aniline was synthesized as previously described.^[30]^ Compound **2 a** was already reported in ref. 28 and compounds **2 c** ^[39]^ and **2 d** ^[39]^ were already described but were synthesized following different procedures.

### Characterization of P‐gp interacting profile and ABC transporter selectivity

The new compounds were tested for their activity toward P‐gp and the other two MDR sister proteins, MRP1 and BCRP, by measuring the inhibition of the transport of the profluorescent probe calcein‐AM (P‐gp and MRP1 substrate) in cells overexpressing P‐gp and MRP1 (MDCK‐MDR1 and MDCK‐MRP1 cells, respectively) and of the fluorescent probe Hoechst 33342 (BCRP substrate) in cells overexpressing BCRP (MDCK‐BCRP cells). In addition, the P‐gp interacting profile of the new compounds was evaluated by the combination of the inhibition of the efflux of calcein‐AM in MDCK‐MDR1 cells test with other two assays: the apparent permeability (*P_app_*) determination in the Caco‐2 cell monolayer, and the ATP cell depletion in MDCK‐MDR1 cells. As previously reported,^[40]^ the apparent permeability (*P_app_*) determination measures the ratio between two fluxes: (1) BA, from the basolateral to apical compartments, representative of passive diffusion; and (2) AB, from the apical to basolateral compartment, influenced by active transport, since P‐gp is apically localized. If the *P*_app_, or BA/AB ratio, is >2, the compound can be considered a P‐gp substrate, since it is able to enter the cell membrane only by passive diffusion, while it is effluxed by P‐gp at the apical level; if the *P*_app_, or BA/AB ratio, is <2, the compound can be considered a P‐gp inhibitor, since it is able to enter the cell membrane avoiding the P‐gp‐mediated efflux at the apical level.^[41]^ This last information is completed by the ATP cell depletion assay, which measures the total ATP cell level in the MDCK cells overexpressing only P‐gp: in this setting, the observed effect can be mainly ascribed to the overexpressed transporter.^[42]^ Only a P‐gp unambiguous substrate (category I), as transported by the pump, induces an ATP consumption, whereas a P‐gp inhibitor does not induce ATP consumption. Compounds displaying a BA/AB >2 but not inducing an ATP cell depletion are classified as class IIB3 substrates.^[43]^

The results of the assays described above are reported in Table 1 together with those of tariquidar and elacridar used as reference compounds. As shown in Table 1, all compounds were able to inhibit the P‐gp‐mediated transport of calcein‐AM, except for compounds **3 b** and **3 c** which resulted not active. Most compounds showed EC_50_ values below 1 μM reaching also the nanomolar range as in the case of compounds **1 d**, **1 e**, **2 a**, **2 c** and **2 e** (EC_50_=36.0 nM, 31.3 nM, 50.0 nM, 85.6 nM and 58.9 nM, respectively).

**Table 1:** Biological results of compounds **1–7**: inhibition activity on MDCK‐MDR1, MDCK‐MRP1 and MDCK‐BCRP cells, overexpressing each transporter P‐gp, MRP1 and BCRP, respectively; ATP cell depletion in MDCK‐MDR1 and apparent permeability (*P_app_*) determination (BA/AB) in Caco‐2 cell monolayer.

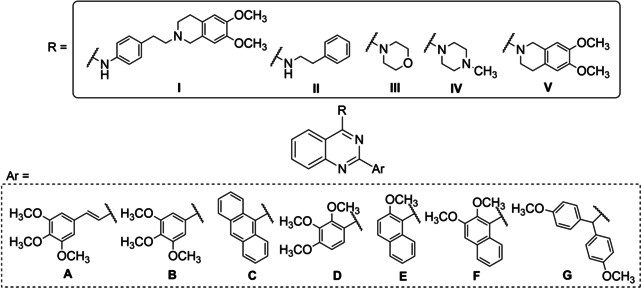							
			EC_50_ μM^[a]^				
Compound	R	Ar	P‐gp	MRP1	BCRP	ATP cell depletion	*P*_app_^[b]^
**1 a**	I	A	0.14±0.02	NA	NA	No	23.4
**1 b**	II	A	0.27±0.04	40.0±8.0	0.40±0.08	No	13.5
**1 c**	III	A	0.37±0.06	7.6±1.50	0.96±0.18	No	9.9
**1 d**	IV	A	0.0360±0.006	52.5±10.0	4.80±0.96	No	9.4
**1 e**	V	A	0.0313±0.005	23.0±4.6	NA	No	23.0
**2 a**	I	B	0.0500±0.001	NA	0.26±0.050	No	16.1
**2 b**	II	B	0.38±0.06	10.0±1.89	0.31±0.06	No	3.6
**2 c**	III	B	0.0856±0.014	3.9±0.66	3.62±0.60	No	6.1
**2 d**	IV	B	1.21±0.22	2.7±0.50	7.70±1.50	No	7.4
**2 e**	V	B	0.0589±0.011	2.1±0.40	NA	No	9.3
**3 a**	I	C	0.38±0.06	NA	NA	No	17.0
**3 b**	II	C	NA	NA	NA	ND	10.5
**3 c**	III	C	NA	NA	NA	ND	20.0
**3 d**	IV	C	8.95±1.60	NA	7.2±1.40	No	9.8
**3 e**	V	C	1.14±0.20	NA	NA	No	18.5
**4 a**	I	D	0.24±0.03	NA	NA	No	6.3
**4 b**	II	D	1.41±0.25	52.0±10.1	NA	No	14.8
**4 c**	III	D	9.55±1.80	NA	NA	No	3.8
**4 d**	IV	D	8.64±1.50	NA	NA	No	3.9
**4 e**	V	D	0.87±0.16	8.47±1.60	NA	No	6.6
**5 a**	I	E	0.20±0.04	NA	NA	No	16.6
**5 b**	II	E	4.00±0.6	NA	NA	No	3.9
**5 c**	III	E	1.25±0.20	NA	NA	No	5.2
**5 d**	IV	E	6.06±1.20	NA	NA	No	5.4
**5 e**	V	E	0.40±0.08	NA	NA	No	5.1
**6 a**	I	F	0.20±0.04	NA	NA	No	19.2
**6 b**	II	F	2.64±0.50	NA	NA	No	6.2
**6 c**	III	F	10.00±1.88	NA	NA	No	5.4
**6 d**	IV	F	1.46±0.28	NA	NA	No	3.4
**6 e**	V	F	0.27±0.05	NA	NA	No	11.8
**7 a**	I	G	0.11±0.02	NA	NA	No	32.9
**7 b**	II	G	0.89±0.16	NA	NA	Yes^[c]^	3.4
**7 c**	III	G	2.13±0.40	NA	NA	No	1.5
**7 d**	IV	G	0.96±0.18	43.2±8.50	8.62±1.60	No	6.9
**7 e**	V	G	0.16±0.026	NA	NA	No	8.4
**tariq**			0.044±0.001	ND	0.010±0.005	Yes^[d]^	>20
**elacr**			0.014±0.003	NA	10.0±2.0	Yes^[e]^	>20

[a] Values are the mean ±SEM of two independent experiments, with samples in triplicate. [b] Apparent permeability estimation: values are from two independent experiments, with samples in duplicate. [c] 50 % at a concentration of 1 μM; [d] 30 % at a concentration of 50 μM; [e] 25 % at a concentration of 10 μM. NA=not active. ND=not determined.

A thorough evaluation of the P‐gp inhibition values indicated that the activity of these compounds was influenced by both the substituents in positions 2 and 4. In fact, the best results were obtained with the aryl residues (*E*)‐3‐(3,4,5‐trimethoxyphenyl)vinyl (**A**) and 3,4,5‐trimethoxyphenyl (**B**) because all derivatives of these two sets showed values in the submicromolar or nanomolar range, except for compound **2 d** (EC_50_=1.21 μM). Otherwise, compounds bearing the aryl moieties **C**, **D**, **E** and **F**, showed low inhibitory effect on P‐gp with EC_50_ values ranging between 0.20 and 10.00 μM. Lastly, as regards the 4,4‐bis(4‐methoxyphenyl)methyl residue (**G**), all the derivatives of this set showed EC_50_ values below 1 μM, except for compound **7 c** (EC_50_=2.13 μM). As regards the R moiety in position 4 of the quinazoline scaffold, the best results, within each set of the series, were obtained for derivatives carrying the 4‐(2‐(6,7‐dimethoxy‐3,4‐dihydroisoquinolin‐2(1*H*)‐yl)ethyl)aniline (**I**), and the 6,7‐dimethoxy‐1,2,3,4‐tetrahydroisoquinoline (**V**) moieties. All these compounds showed EC_50_ values in the submicromolar or nanomolar range except compound **3 e** (EC_50_=1.14 μM). Anyway, in the set with Ar=**A** the most potent compounds were **1 d** and **1 e** that are characterized by R=**IV** and **V**, respectively.

Differently from the activity on P‐gp, the inhibitory activity on MRP1 appeared to be mainly influenced by the nature of the aryl moieties in position 2 of the quinazoline scaffold. Indeed, all compounds of the sets with the aryl residues anthracene (**C**), 2‐methoxynaphthalene (**E**) and 2,3‐dimethoxynaphthalene (**F**) were inactive on this transporter. Otherwise, compounds **2 c**, **2 d** and **2 e**, carrying Ar=**B**, were the most potent with EC_50_=3.9 μM, 2.7 μM and 2.1 μM, respectively. Moreover, derivatives **1 c** (Ar=**A**), **2 b** (Ar=**B**) and **4 e** (Ar=**D**) showed EC_50_ values below or equal to 10 μM (EC_50_=7.6 μM, 10.0 μM and 8.5 μM, respectively) and compounds **1 b**, **1 d**, **1 e**, **4 b** and **7 d** showed a modest MRP1 inhibitory activity (EC_50_=40.0 μM, 52.5 μM, 23.0 μM, 52.0 μM and 43.2 μM, respectively).

The aryl moieties in position 2 also influenced the inhibitory activity on BCRP. In this case, all the compounds of the sets with the aryl residues 2,3,4‐trimethoxyphenyl (**D**), 2‐methoxynaphthalene (**E**) and 2,3‐dimethoxynaphthalene (**F**) were inactive on this transporter. Differently, the most potent compounds were **1 b** and **1 c** (Ar=**A**), and **2 a** and **2 b** (Ar=**B**) with EC_50_ values below 1 μM (EC_50_=0.40 μM, 0.96 μM, 0.26 μM and 0.31 μM, respectively). Moreover, **1 d**, **2 c**, **2 d**, **3 d** and **7 d** showed a moderate activity on BCRP with EC_50_ values ranging between 3.62 and 8.32 μM. Interestingly, most of the compounds able to modulate the BCRP activity also showed a significant effect on P‐gp and MRP1 except for compounds **2 a** and **3 d** that were inactive towards MRP1. Therefore, compound **2 a** showed the best combination of activity on P‐gp and BCRP (EC_50_=0.05 μM on P‐gp and EC_50_=0.26 μM on BCRP) and compound **1 e** was the most active and selective P‐gp ligand (EC_50_=31.3 nM).

As regards the P‐gp interacting profile, the apparent permeability determination *P_app_* (BA/AB) in the Caco‐2 cell monolayer indicated that only compound **7 c**, having a *P_app_* <2, inhibiting calcein‐AM transport and do not inducing ATP cell depletion, may be defined as P‐gp inhibitor. Compound **7 b** may be defined as a P‐gp unambiguous substrate (category I) since it was able to induce ATP cell depletion and to inhibit calcein‐AM transport with a *P_app_* >2. The other compounds (except derivatives **3 b** and **3 c** that were not active on P‐gp) behaved as not transported substrates (category IIB3) since they showed a *P_app_* >2, inhibited calcein‐AM transport and they did not induce ATP cell depletion. In general, all compounds bearing residues **I** show the highest *P_app_* values within each set, except for the set with the aryl residues anthracen‐9‐yl (**C**) and 2,3,4‐ trimethoxyphenyl (**D**) for which the highest *P_app_* values was obtained with residues **III** and **II**, respectively. Considering that a compound can cross membranes in several ways, the high *P*_app_ values indicate that the compounds behave as P‐gp substrates, thus they are not able to cross membranes where P‐gp is present since they are taken out by the pump, and the contribution of passive diffusion is relevant. The lipophilicity of the substituents play an important role in giving the compounds the characteristics suitable for crossing the membrane. Among the R substituents, **I** and **V** are those with a higher lipophilicity and this justifies the greater ability to cross the membrane by passive diffusion (where P‐gp is not present).

### Molecular modeling studies

In order to give a sensible explanation of the activity profile of target compounds towards P‐gp, a molecular docking study was performed using the crystal structure of P‐gp in its inward conformation (PDB code 4XWK).^[44]^ The simulation was carried out using Gold software v. 2020.2.0.^[45]^ The internal surface of the transmembrane region of P‐gp was set as interaction site. After a first run of rigid docking, for each compound a second run was carried out, setting flexibility for relevant residues in the binding region: the best poses of this second computation were selected for analysis.

**Figure 3:**
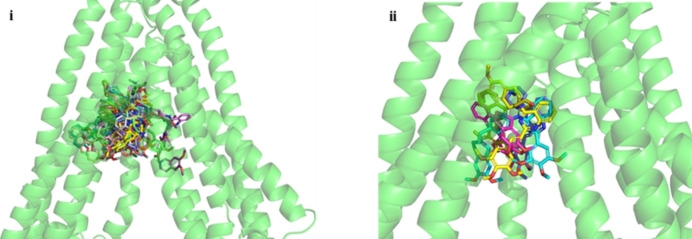
Collective picture of binding poses of the entire set of studied compounds (**i**) and of the most active ones (**1 d**, **1 e**, **2 a**, **2 c** and **2 e**) (**ii**) within the P‐gp binding region.

**Figure 4:**
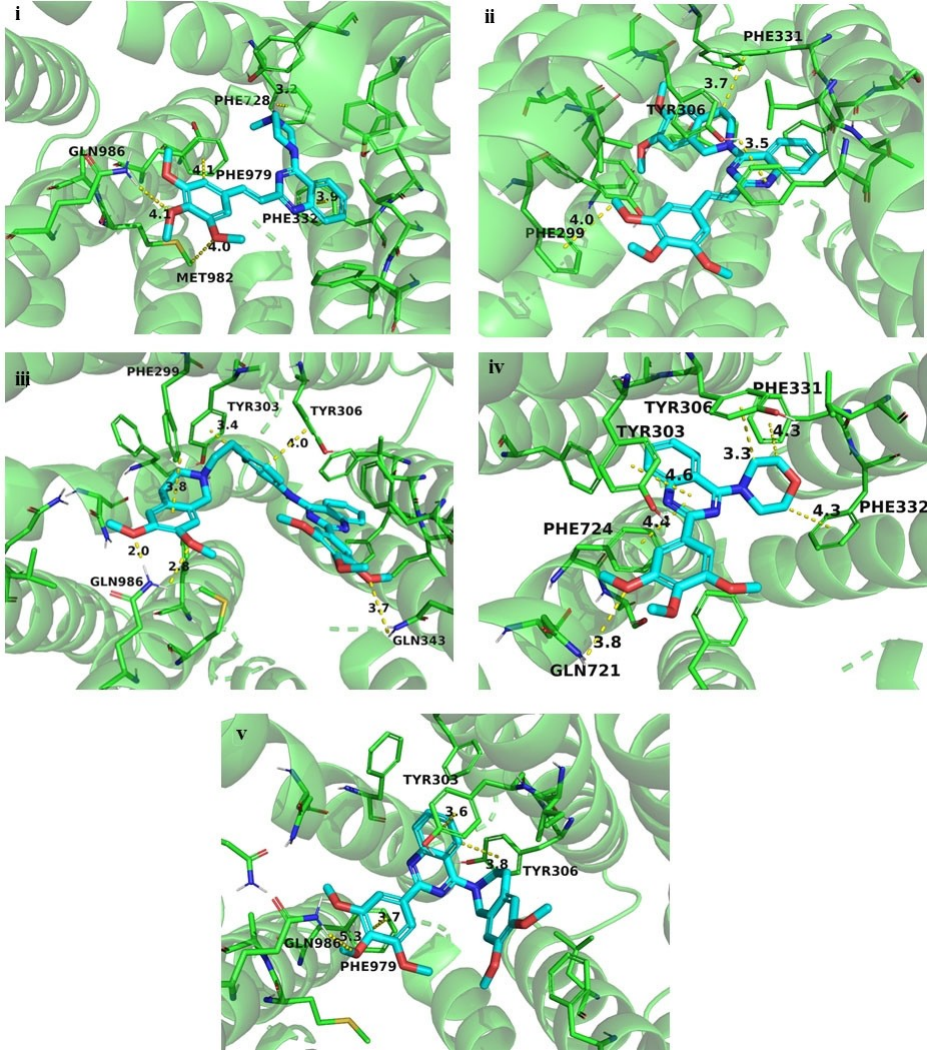
Binding pose of the most active compounds **1 d** (**i**), **1 e** (**ii**), **2 a** (**iii**), **2 c** (**iv**) and **2 e** (**v**) within the P‐gp binding region.

As can be seen in Figure 3 i, there is not a common recognizable binding pattern for the studied compounds, which span nearly throughout the whole transmembrane region. Nevertheless, some structural features are worth noting for selected compounds. In particular, the most potent compounds, **1 d**, **1 e**, **2 a**, **2 c** and **2 e**, bearing aryl groups **A** or **B**, exhibited some common peculiarities with binding poses in a quite delimited region, thus suggesting a possible key for their potency (Figure 3 ii). The interactions with receptor residues are mostly hydrophobic with few or, in some cases, no polar contacts. The transmembrane domains (TM) mainly involved in binding are TM6, TM7 and TM12 (Figures 4 i‐v).

For all the compounds the quinazoline ring is placed as a “pivot”. Depending on the specific position of this moiety, two different patterns can be identified for **1 d**, **1 e**, and **2 a**, and for **2 c** and **2 e**. The 3,4,5‐trimethoxyphenyl ring is projected towards the lower limit of the transmembrane region and establishes contacts with TM7 and TM12 (**1 d**, **2 c** and **2 e**) and with TM6 (**1 e**) and TM12 (**2 a**). In case of **1 d** and, to a lesser extent, **2 e**, this moiety is able to give polar contacts with Gln986 side chain. The other “arm” of these two molecules is kept in the apical part of the binding region and gives additional hydrophobic contacts (cation‐π in case of **1 d**).

Compounds **2 a** and **2 e** display a slightly different binding mode: the two “arms” are both directed downward in a nutcracker fashion, giving hydrophobic interactions and, in case of **2 a**, polar contacts with Gln986 and with Gln343.

**Figure 5:**
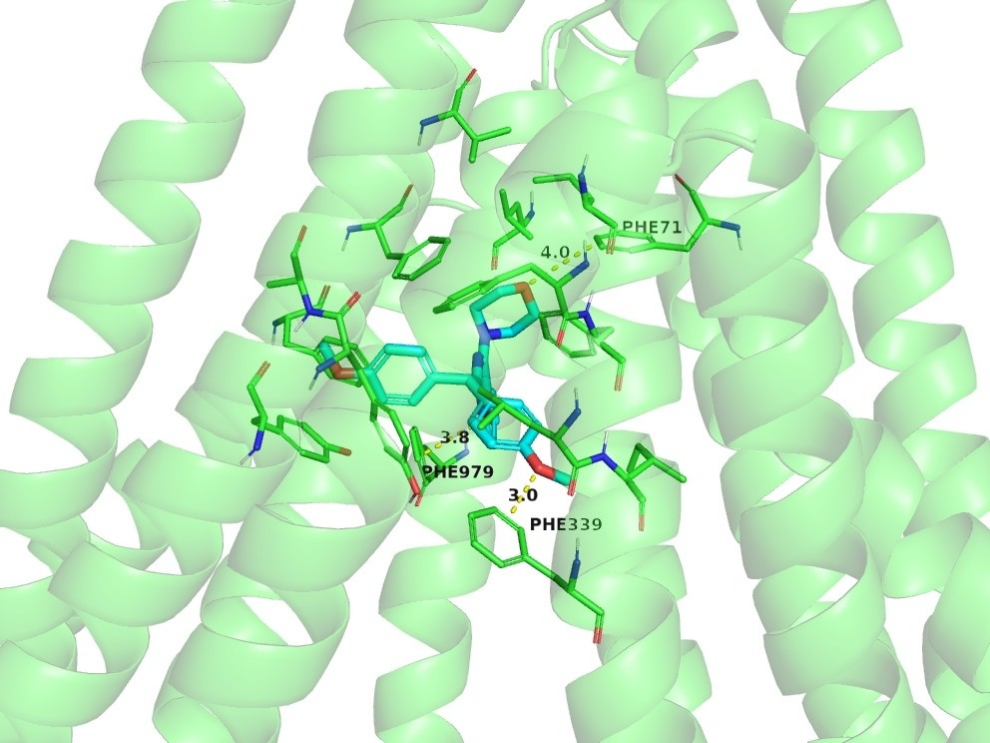
Binding pose within the P‐gp binding region of compound **7 c**, behaving as a pure inhibitor.

A further consideration deserves compound **7 c**, which is the only ligand of the set showing a behaviour of pure inhibitor, with a BA/AB ratio lower than 2, even if with a low potency. This compound is characterized by a binding pose in a very apical position inside the internal cavity of P‐glycoprotein (Figure 5). This localization enables the compound to reach four different domains (TM1, TM6 and TM12) with weak hydrophobic interactions. The peculiar pose of the compound and its “cross‐linking” ability could be an explanation of the functional profile of this ligand that is able to block the protein, rather than being transported.

### Co‐administration assays

**Figure 6:**
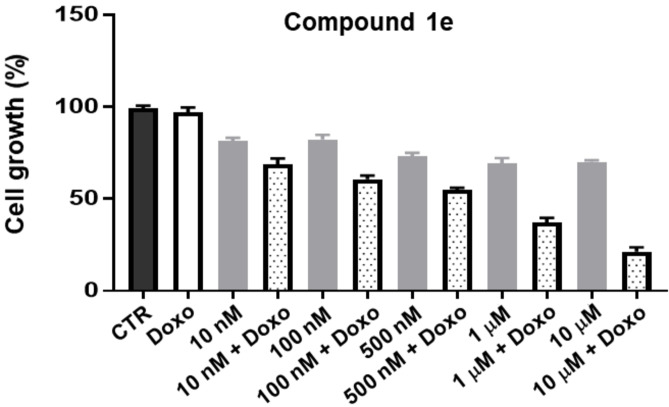
Antiproliferative activity on MDCK‐MDR1 cells of doxorubicin (Doxo) at 10 μM and compound **1 e** at 10 nM, 100 nM, 500 nM, 1 μM and 10 μM, alone and in co‐administration with doxorubicin 10 μM. Each bar represents the mean ±SEM of two experiments performed in triplicate. One‐way analysis of variance (ANOVA) analysis: ****p<0.0001 *vs* control.

Starting from the results reported in Table 1, we selected the highly active and selective P‐gp ligand, compound **1 e**, to study its ability to restore the cytotoxic activity of the antineoplastic drug doxorubicin in a co‐administration assay in the “pure” model of cells overexpressing P‐gp (MDCK‐MDR1 cells), representative for resistant cancers. Doxorubicin, being a P‐gp substrate, is effluxed by the pump out from the cell membranes, with a consequent reduction of its cytotoxic activity. Thus, we tested compound **1 e** alone, to evaluate its intrinsic cytotoxicity, and in the presence of the chemotherapeutic drug at 10 μM, to evaluate its ability to increase the doxorubicin cytotoxic activity. As depicted in Figure 6, compound **1 e** alone shows an intrinsic cytotoxicity of around 20–30 % at each tested dose. In the co‐administration assay, the doxorubicin cytotoxicity increased by 50 % already at the dose of 500 nM of compound **1 e**, reaching an increase of 80 % with 10 μM of compound **1 e**.

**Figure 7:**
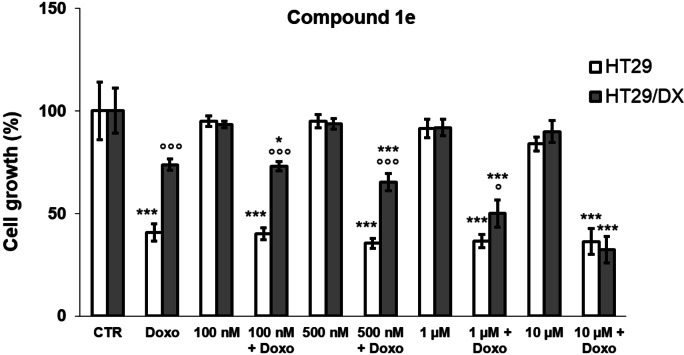
Antiproliferative activity on HT29 and HT29/DX cells of doxorubicin (Doxo) at 10 μM, alone and in co‐administration with compound **1 e** at 100 nM, 500 nM, 1 μM and 10 μM. Each bar represents the mean ± SEM of two experiments performed in triplicate. One‐way analysis of variance (ANOVA) analysis: *p<0.05; ***p<0.001 *vs* control; ° *p*<0.05; °°°*p*<0.001: HT29/DX cells *vs* respective HT29 cells.

The decreased viability induced by compound **1 e** was also observed in the HT29 colon cancer model and in its doxorubicin‐resistant counterpart HT29/DX (Figure 7). Compound **1 e** alone did not significantly reduce cell viability neither in doxorubicin‐sensitive nor in doxorubicin‐resistant cells.

While in the lowly P‐gp expressing HT29 cells,^[32]^ a significant decrease in viable cells treated with doxorubicin was shown, this is not the case in the highly P‐gp expressing HT29/DX cells,^[32]^ as expected. In the HT29 cell line, compound **1 e** did not increase the cytotoxic potential of doxorubicin at 10 μM, but it did so in HT29/DX cells in a dose‐dependent manner. Interestingly, when doxorubicin was co‐administered with **1 e** at 10 μM concentration, the viability of doxorubicin‐resistant cells was reduced at the same extent of doxorubicin‐sensitive cells.

**Figure 8:**
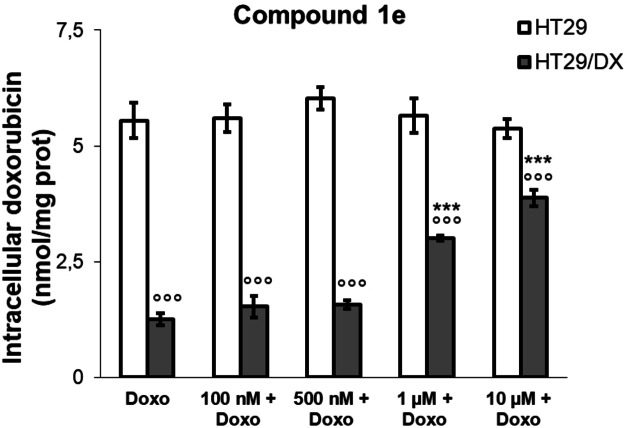
Intracellular accumulation of doxorubicin in HT29 and HT29/DX cells, incubated 24 h with doxorubicin (Doxo) at 10 μM, alone and in co‐administration with compound **1 e** at 100 nM, 500 nM, 1 μM and 10 μM. Each bar represents the mean ± SEM of two experiments performed in triplicate. One‐way analysis of variance (ANOVA) analysis: ***p<0.001: HT29/DX *vs* HT29 cells; °°°*p*<0.001: *vs* Doxo alone.

To investigate whether the reduced viability elicited by compound **1 e** plus doxorubicin in HT29/DX cells was due to a different retention of the anthracycline within the cells, we measured the intracellular content of doxorubicin in cells treated with increasing concentrations of **1 e**. As shown in Figure 8, HT29/DX cells retained a lower amount of doxorubicin, in line with the higher levels of P‐gp. Again, derivative **1 e** did not modify the intracellular drug content in HT29 cells, while it progressively increased it in HT29/DX cells. This difference is likely due to the inhibition of P‐gp efflux activity exerted by **1 e** in resistant cells.

Mechanistically, compound **1 e** increased the Km of doxorubicin in HT29/DX cells, without affecting the maximal velocity (Vmax) of the efflux that remained higher in HT29/DX cells compared to HT29 cells (Table 2).

**Table 2:** Kinetic parameters of doxorubicin efflux in HT29 and HT29/DX cells.

Cell line^[a]^	Condition	Km	Vmax
HT29	Doxo	0.53±0.06	3.15±0.07
HT29	Doxo+**1 e**	0.55±0.08	3.39±0.03
HT29/DX	Doxo	0.52±0.06	8.05±0.64***
HT29/DX	Doxo+**1 e**	0.63±0.03°	7.90±0.72***

[a] HT29 and HT29/DX cells were grown in the absence or presence of 10 μM compound **1 e**, with increasing concentrations of doxorubicin (Doxo) for 24 h. Km (μM) and Vmax (μmoles/min) were calculated with the Enzfitter software. ***p<0.001: HT29/DX *vs* HT29 cells; ° p<0.05: HT29/DX *vs* HT29 cells.

Since Vmax is related to the amount of P‐gp present on cell surface,^[46]^ it is not unexpected that HT29/DX displayed a higher value. Differently from other P‐gp inhibitors recently synthesized by our group,^[47]^ compound **1 e** did not change Vmax. Therefore, it is unlikely that it affects the amount of P‐gp present in the plasma membrane. Interestingly, compound **1 e** increased the Km, indicating a reduced affinity of doxorubicin for P‐gp.^[46]^

## Conclusions

In this work we reported a new series of 2,4‐disubstituted quinazoline derivatives able to modulate the ABC transporters involved in multidrug resistance (MDR). The quinazoline‐4‐amine scaffold was chosen due to its presence in the structure of many ABC transporters modulators, such as the tyrosine kinase inhibitors (TKIs) gefitinib and erlotinib. For this reason, in these compounds the quinazoline moiety was substituted in position 4 with secondary or tertiary protonable amines, and in position 2 with the anthracene or methoxy‐substituted aryl moieties, that were already present in potent and efficacious P‐gp dependent MDR reversers. The new compounds were tested on MDCK transfected cells: MDCK‐MDR1, MDCK‐MRP1 and MDCK‐BCRP cells overexpressing P‐gp, MRP1 and BCRP, respectively. Concerning the activity on P‐gp, almost all compounds were able to inhibit its transporter activity and some derivatives showed EC_50_ values in the nanomolar range (**1 d**, **1 e**, **2 a**, **2 c** and **2 e**). This activity was mainly influenced by the nature of aryl moiety in position 2, and the (*E*)‐3‐(3,4,5‐trimethoxyphenyl)vinyl (**A**) and 3,4,5‐trimethoxyphenyl (**B**) residues, resulted the most favorable ones. However, also the nature of the R moiety in position 4 of the quinazoline scaffold influenced the P‐gp inhibitory effect of these compounds and in general the best results, within each set of the series, were obtained by the presence of the 4‐(2‐(6,7‐dimethoxy‐3,4‐dihydroisoquinolin‐2(1*H*)‐yl)ethyl)aniline (**I**), and the 6,7‐dimethoxy‐1,2,3,4‐tetrahydroisoquinoline (**V**).

Most of the compounds were inactive on MRP1 and/or BCRP, except for almost all the derivatives bearing Ar=**A** and **B**, and compounds **3 d** (Ar=**C**), **4 b** (Ar=**D**), **4 e** (Ar=**D**) and **7 d** (Ar=**G**). Interestingly, compound **2 a** was active towards both P‐gp and BCRP, as well as the reference compounds tariquidar and elacridar. The good activities of compound **2 a** on these two proteins (EC_50_=0.05 μM and 0.26 μM, respectively) is an interesting feature since P‐gp and BCRP are often co‐expressed in several tumours.

As for the P‐gp interacting profile, these compounds behaved as not transported substrates (category IIB3), except compound **7 b** which may be defined as a P‐gp unambiguous substrate (category I), and compound **7 c** which may be considered a P‐gp inhibitor.

To explain the activity of this series of compounds, a molecular docking study was carried out on the crystal structure of P‐gp in its inward conformation (PDB code 4XWK). Some common features were highlighted for the most potent compounds, **1 d**, **1 e**, **2 a**, **2 c** and **2 e**, which exhibited mostly hydrophobic interactions and few or, in some case, no polar contacts with the receptor residues in a quite delimited region. The pure inhibitor compound **7 c** showed a peculiar binding pose in a very apical position inside the internal P‐gp cavity giving hydrophobic interactions with four different domains. Its “cross‐linking” ability could explain its functional profile.

For its high P‐gp activity and selectivity, compound **1 e** was selected for resistant reversion studies both in a pure model of P‐gp overexpressing cells (MDCK‐MDR1 cells) and in a model of acquired resistance to doxorubicin (HT29/DX cells). In co‐administration of 10 μM of doxorubicin in MDCK‐MDR1 cells, compound **1 e** was able to enhance the doxorubicin cytotoxicity reaching values of 70 and 80 % at 1 μM and 10 μM, respectively. Also in HT29/DX cells, in the co‐administration of doxorubicin (10 μM), compound **1 e** was able to restore the doxorubicin cytotoxicity in a dose‐dependent manner until the same extent of doxorubicin‐sensitive cells. Moreover, compound **1 e** was able to enhance the intracellular content of doxorubicin in HT29/DX cells in the co‐administration of doxorubicin (10 μM), confirming that its resistant reversion ability is due to the P‐gp interaction. The P‐gp inhibitory activity of compound **1 e**, was also proved by the evaluation of its influence on P‐gp kinetic parameters; in fact, it was able to increase the Km value of doxorubicin without modifying the Vmax value. These results showed that this compound was able to reduce the doxorubicin affinity for P‐gp without affecting the P‐gp amount in the membrane.

In summary, in this study we identified some potent P‐gp inhibitors endowed with activity in the nanomolar range. In particular, compound **1 e**, displaying the best P‐gp activity and selectivity, was able to increase the uptake of doxorubicin in resistant cancer cells restoring its antineoplastic activity. Moreover, compound **2 a** showed good activities towards both P‐gp and BCRP, showing a dual inhibitory effect.

## Experimental Section

### Chemistry

All melting points were taken on a Büchi apparatus and are uncorrected. NMR spectra were recorded on a Bruker Avance 400 spectrometer (400 MHz for ^1^H‐NMR, 100 MHz for ^13^C‐NMR). ^1^H and ^13^C NMR spectra were measured at room temperature (25 °C) in an appropriate solvent. ^1^H and ^13^C chemical shifts are expressed in ppm (δ) referenced to TMS. Spectral data are reported using the following abbreviations: bs=broad singlet, s=singlet, d=doublet, t=triplet, q=quartet, m=multiplet, and coupling constants are reported in Hz, followed by integration. Assignments of the ^13^C signals were performed using the attached proton test (APT) technique.

Chromatographic separations were performed on a silica gel column by flash chromatography (Kieselgel 40, 0.040–0.063 mm; Merck). Yields are given after purification, unless otherwise stated. The high‐resolution mass spectrometry (HRMS) analysis was performed with a Thermo Finnigan LTQ Orbitrap mass spectrometer equipped with an electrospray ionization source (ESI). The accurate mass measure was carried out by introducing, via syringe pump at 10 μL min^−1^, the sample solution (1.0 μg mL^−1^ in mQ water: acetonitrile 50 : 50), and the signal of the positive ions was acquired. The proposed experimental conditions allowed to monitoring the protonated molecules of studied compounds ([M+H]^+^ species), that they were measured with a proper dwell time to achieve 60 000 units of resolution at Full Width at Half Maximum (FWHM). The elemental composition of compounds was calculated on the basis of their measured accurate masses, accepting only results with an attribution error less than 2.5 ppm and a not integer RDB (double bond/ring equivalents) value, in order to consider only the protonated species.^[48]^

Compounds were named following IUPAC rules as applied by ChemBioDraw Ultra 14.0 software. When reactions were performed in dry conditions, the mixtures were maintained under nitrogen. Free bases **1**–**7** (except for **3 b**) were transformed into the hydrochloride by treatment with a solution of acetyl chloride (1.2 equiv./N atom) in dry CH_3_OH. The salts were crystallized from abs. ethanol/petroleum ether.

#### (*E*)‐2‐(3,4,5‐trimethoxystyryl)‐4*H*‐benzo[*d*][1,3]oxazin‐4‐one (8)

Following the procedure described in ref. 28, starting from 2‐aminobenzoic acid (0.87 g, 6.33 mmol) and (*E*)‐3‐(3,4,5‐trimethoxyphenyl)acryloyl chloride (1.08 g, 4.21 mmol) in 5.0 mL of dry pyridine, compound **8** (0.32 g, 22.1 %) was obtained as a yellow solid.

TLC: CH_2_Cl_2_/CH_3_OH/CH_3_COOH 99 : 1 : 1. ^1^H‐NMR (400 MHz, CDCl_3_) δ: 8.12 (d, *J*=7.6 Hz, 1H, CH arom.); 7.23 (t, *J*=7.6 Hz, 1H, CH arom.); 7.69 (d, *J*=16.4 Hz, 1H, C*H*=CH); 7.51 (d, *J*=7.6 Hz, 1H, CH arom.); 7.42 (t, *J*=7.6 Hz, 1H, CH arom.); 6.75 (s, 2H, CH arom.); 6.63 (d, *J*=16.4 Hz, 1H, C*H*=CH); 3.84 (s, 9H, OCH_3_) ppm.

#### 2‐(3,4,5‐Trimethoxyphenyl)‐4*H*‐benzo[*d*][1,3]oxazin‐4‐one (9)

This compound was already described in ref. 28.

#### (*E*)‐2‐(3,4,5‐Trimethoxystyryl)quinazolin‐4(3*H*)‐one (10)^[36]^

Following the procedure described in ref. 28, starting from **8** (0.46 g, 1.36 mmol) and 33.0 % ammonia water (3.0 mL) in 7.0 mL of abs. ethanol, compound **10** (0.35 g, yield: 76.9 %) was obtained as a yellow solid.

TLC: CH_2_Cl_2_/CH_3_OH 96 : 4. ^1^H‐NMR (400 MHz, CDCl_3_) δ: 11.45 (bs, 1H, NH); 8.29 (d, *J*=8.0 Hz, 1H, CH arom.); 7.84 (d, *J*=16.4 Hz, 1H, C*H*=CH); 7.79‐7.71 (m, 2H, CH arom.); 7.42 (t, *J*=8.0 Hz, 1H, CH arom.); 6.87 (s, 2H, CH arom.); 6.86 (d, *J*=16.4 Hz, 1H, C*H*=CH); 3.92 (s, 6H, OCH_3_); 3.89 (s, 3H, OCH_3_) ppm.

#### 2‐(3,4,5‐Trimethoxyphenyl)quinazolin‐4(3*H*)‐one (11)

This compound was already described in ref. 28.

#### General procedure for the synthesis of quinazolin‐4(3*H*)‐ones (12‐15)

Following the procedure described in ref. 33, to a solution of anthranilamide (1 equiv.) and the proper aldehyde (1 equiv.) in the adequate amount of ethanol, CuCl_2_ (2 equiv.) was added. The reaction mixture was refluxed for 16 h, then was cooled to rt. A proper amount of water was added, yielding a green solid that was filtered, dried under vacuum, and purified by flash chromatography. Finally, quinazolin‐4(3*H*)‐ones **12–15** were obtained as pure solids.

#### 2‐(Anthracen‐9‐yl)quinazolin‐4(3*H*)‐one (12)^[37]^

Starting from anthranilamide (0.099 g, 0.73 mmol) and anthracene‐9‐carbaldehyde (0.15 g, 0.73 mmol) in 3.6 mL of ethanol, compound **12** (0.23 g, yield: 100.0 %) was obtained as a green solid.

Chromatographic eluent: CH_2_Cl_2_/CH_3_OH/NH_4_OH 99 : 1:0.1. ^1^H‐NMR (400 MHz, CDCl_3_) δ: 9.69 (bs, 1H, NH); 8.54 (s, 1H, CH arom.); 8.24 (d, *J*=8.0 Hz, 1H, CH arom.); 8.02–7.98 (m, 2H, CH arom.); 7.89–7.85 (m, 2H, CH arom); 7.84–7.80 (m, 2H, CH arom.); 7.57 (t, *J*=8.0 Hz, 1H, CH arom.); 7.48–7.43 (m, 4H, CH arom.) ppm.

#### 2‐(2,3,4‐Trimethoxyphenyl)quinazolin‐4(3*H*)‐one (13)^[38]^

Starting from anthranilamide (0.35 g, 2.55 mmol) and 2,3,4‐trimethoxybenzaldehyde (0.50 g, 2.55 mmol) in 14.0 mL of ethanol, compound **13** (0.80 g, yield: 100.0 %) was obtained as a white solid.

Chromatographic eluent: CH_2_Cl_2_/CH_3_OH 95 : 5. ^1^H‐NMR (400 MHz, CDCl_3_) δ: 11.03 (bs, 1H, NH); 8.28 (d, *J*=8.0 Hz, 1H, CH arom.); 8.23 (d, *J*=9.2 Hz, 1H, CH arom.); 7.82‐7.70 (m, 2H, CH arom.); 7.44 (t, *J*=8.0 Hz, 1H, CH arom.); 6.86 (d, *J*=9.2 Hz, 1H, CH arom.); 4.04 (s, 3H, OCH_3_); 3.94 (s, 3H, OCH_3_); 3.91 (s, 3H, OCH_3_) ppm.

#### 2‐(2‐Methoxynaphthalen‐1‐yl)quinazolin‐4(3*H*)‐one (14)

Starting from anthranilamide (0.44 g, 3.22 mmol) and 2‐methoxy‐1‐naphthaldehyde (0.60 g, 3.22 mmol) in 24.0 mL of ethanol, compound **14** (0.87 g, yield: 89.3 %) was obtained as a white solid.

Chromatographic eluent: CH_2_Cl_2_/CH_3_OH/NH_4_OH 98 : 2:0.2. ^1^H‐NMR (400 MHz, CDCl_3_) δ: 9.94 (bs, 1H, NH); 8.30 (d, *J*=8.0 Hz, 1H, CH arom.); 8.00 (d, *J*=9.2 Hz, 1H, CH arom.); 7.95‐7.77 (m, 4H, CH arom.); 7.54 (t, *J*=8.0 Hz, 1H, CH arom.); 7.46 (t, *J*=8.0 Hz, 1H, CH arom.); 7.38 (t, *J*=8.0 Hz, 1H, CH arom.); 7.34 (d, *J*=9.2 Hz, 1H, CH arom.); 3.91 (s, 3H, OCH_3_) ppm.

#### 2‐(2,3‐Dimethoxynaphthalen‐1‐yl)quinazolin‐4(3*H*)‐one (15)

Starting from anthranilamide (0.22 g, 1.62 mmol) and 2,3‐dimethoxy‐1‐naphthaldehyde (0.35 g, 1.62 mmol) in 14.0 mL of ethanol, compound **15** (0.46 g, yield: 85.5 %) was obtained as a yellow solid.

Chromatographic eluent: CH_2_Cl_2_/CH_3_OH/NH_4_OH 98 : 2:0.2. ^1^H‐NMR (400 MHz, CDCl_3_) δ: 10.94 (bs, 1H, NH); 8.29 (d, *J*=7.6 Hz, 1H, CH arom.); 7.84–7.74 (m, 2H, CH arom.); 7.72 (d, *J*=7.6 Hz, 1H, CH arom.); 7.61 (d, *J*=7.6 Hz, 1H, CH arom); 7.49 (t, *J*=7.6 Hz, 1H, CH arom.); 7.38 (t, *J*=7.6 Hz, 1H, CH arom.); 7.31 (t, *J*=7.6 Hz, 1H, CH arom.); 6.62 (s, 1H, CH arom.); 3.84 (s, 3H, OCH_3_); 3.15 (s, 3H, OCH_3_) ppm.

#### 2‐(Bis(4‐methoxyphenyl)methyl)quinazolin‐4(3*H*)‐one (16)

Following the procedure described in ref. 37, starting from 2,2‐bis(4‐methoxyphenyl)acetic acid^[35]^ (0.50 g, 1.84 mmol), HATU (0.84 g, 2.21 mmol) and DIPEA (0.64 g, 3.68 mmol) in 13.0 mL of dry CH_2_Cl_2_, compound **16** (0.60 g, yield: 87.6 %) was obtained as a white solid.

TLC: CH_2_Cl_2_/CH_3_OH 95 : 5. ^1^H‐NMR (400 MHz, CDCl_3_) δ: 9.50 (bs, 1H, NH); 8.23 (d, *J*=7.6 Hz, 1H, CH arom.); 7.78–7.71 (m, 2H, CH arom.); 7.47 (t, *J*=7.6 Hz, 1H, CH arom.); 7.17 (d, *J*=8.8 Hz, 4H, CH arom.); 6.86 (d, *J*=8.8 Hz, 4H, CH arom.); 5.51 (s, 1H, CH); 3.77 (s, 6H, OCH_3_) ppm.

### General procedure for the synthesis of 4‐chloroquinazolines (17‐19 and 21,22).

The procedure described in ref. 28 was followed with slight modifications: to a solution of the quinazolin‐4(3*H*)‐one (1 equiv.) in the adequate amount of CHCl_3_ (free of ethanol), SOCl_2_ (10 equiv.) and 3 drops of dry DMF were added. The reaction mixture was stirred at 50 °C for 6 h, then cooled to rt and the solvent was removed under vacuum. The residue was treated twice with cyclohexane and the solvent was removed under reduced pressure. The obtained red solid was suspended into a 1 N NaOH solution, stirred for 10 minutes, and then treated with CH_2_Cl_2_. The organic layer was washed twice with water and brine, dried over Na_2_SO_4_, and concentrated under vacuum, to afford the proper 4‐chloroquinazoline as a solid.

#### (*E*)‐4‐Chloro‐2‐(3,4,5‐trimethoxystyryl)quinazoline (17)^[36]^

Starting from **10** (0.24 g, 0.71 mmol) and SOCl_2_ (0.50 mL, 7.10 mmol) in 10.0 mL of CHCl_3_ (free of ethanol), compound **17** (0.23 g, yield: 91.8 %) was obtained as a yellow solid.

TLC: CH_2_Cl_2_/CH_3_OH/NH_4_OH 98 : 2:0.2. ^1^H‐NMR (400 MHz, CDCl_3_) δ: 8.04 (d, *J*=8.4 Hz, 1H, CH arom.); 7.90 (d, *J*=16.0 Hz, 1H, C*H*=CH); 7.83 (d, *J*=8.4 Hz, 1H, CH arom.); 7.77 (t, 1H, *J*=7.2 Hz, CH arom.); 7.48 (t, *J*=7.2 Hz, 1H, CH arom.); 7.08 (d, *J*=16.0 Hz, 1H, C*H*=CH); 6.77 (s, 2H, CH arom.); 3.81 (s, 9H, OCH_3_) ppm.

#### 4‐Chloro‐2‐(3,4,5‐trimethoxyphenyl)quinazoline (18)

The compound was already described in ref. 28.

#### 2‐(Anthracen‐9‐yl)‐4‐chloroquinazoline (19)

Starting from **12** (0.24 g, 0.74 mmol) and SOCl_2_ (0.54 mL, 7.40 mmol) in 8.0 mL of CHCl_3_ (free of ethanol), compound **19** (0.23 g, yield: 90.6 %) was obtained as an orange solid.

TLC: CH_2_Cl_2_/CH_3_OH/NH_4_OH 98 : 2:0.2. Yield: 90.6 %. ^1^H‐NMR (400 MHz, CDCl_3_) δ: 8.61 (s, 1H, CH arom.); 8.46 (d, *J*=8.0 Hz, 1H, CH arom.); 8.23 (d, *J*=8.0 Hz, 1H, CH arom.); 8.09–8.06 (m, 3H, CH arom.); 7.86 (t, *J*=8.0 Hz, 1H, CH arom.); 7.64 (d, *J*=8.4 Hz, 2H, CH arom.); 7.47 (t, *J*=8.4 Hz, 2H, CH arom.) 7.41 (t, *J*=8.4 Hz, 2H, CH arom.) ppm.

#### 4‐Chloro‐2‐(2‐methoxynaphthalen‐1‐yl)quinazoline (21)

Starting from **14** (0.87 g, 2.88 mmol) and SOCl_2_ (2.10 mL, 28.80 mmol) in 16.0 mL of CHCl_3_ (free of ethanol), compound **21** (0.81 g, yield: 88.8 %) was obtained as a pale‐yellow solid.

TLC: CH_2_Cl_2_/CH_3_OH/NH_4_OH 98 : 2:0.2. ^1^H‐NMR (400 MHz, CDCl_3_) δ: 8.37 (d, *J*=8.0 Hz, 1H, CH arom.); 8.18 (d, *J*=8.4 Hz, 1H, CH arom.); 8.06‐7.93 (m, 2H, CH arom.); 7.88–7.81 (m, 1H, CH arom.); 7.78 (t, *J*=8.4 Hz, 1H, CH arom.); 7.43–7.29 (m, 4H, CH arom.); 3.89 (s, 3H, OCH_3_) ppm.

#### 4‐Chloro‐2‐(2,3‐dimethoxynaphthalen‐1‐yl)quinazoline (22)

Starting from **15** (0.46 g, 1.38 mmol) and SOCl_2_ (1.00 mL, 13.84 mmol) in 12.0 mL of CHCl_3_ (free of ethanol), compound **22** (0.36 g, yield: 74.1 %) was obtained as a pale‐yellow solid.

TLC: CH_2_Cl_2_/CH_3_OH 98 : 2. ^1^H‐NMR (400 MHz, CDCl_3_) δ: 8.38 (d, *J*=8.4 Hz, 1H, CH arom.); 8.18 (d, *J*=8.4 Hz, 1H, CH arom.); 8.02 (t, *J*=7.6 Hz, 1H, CH arom.); 7.80 (t, *J*=7.6 Hz, 1H, CH arom.); 7.76 (d, *J*=8.4 Hz, 1H, CH arom.); 7.43‐7.34 (m, 2H, CH arom.); 7.31 (s, 1H, CH arom.); 7.26 (t, J=8.4 Hz, 1H, CH arom.); 4.03 (s, 3H, OCH_3_); 3.92 (s, 3H, OCH_3_) ppm.

#### 4‐Chloro‐2‐(2,3,4‐trimethoxyphenyl)quinazoline (20)

Following the procedure described in ref. 27, starting from **13** (0.73 g, 2.34 mmol) and POCl_3_ (6.80 mL, 75.79 mmol), compound **20** (0.58 g, yield: 75.1 %) was obtained as a yellow solid.

TLC: CH_2_Cl_2_/CH_3_OH/NH_4_OH 98 : 2:0.2. ^1^H‐NMR (400 MHz, CDCl_3_) δ: 8.26 (d, *J*=8.0 Hz, 1H, CH arom.); 8.12 (d, *J*=8.0 Hz, 1H, CH arom.); 7.94 (t, *J*=8.0 Hz, 1H, CH arom.); 7.74 (d, *J*=8.8 Hz, 1H, CH arom.); 7.68 (t, *J*=8.0 Hz, 1H, CH arom.); 6.82 (d, *J*=8.8 Hz, 1H, CH arom.); 4.04 (s, 3H, OCH_3_); 3.92 (s, 6H, OCH_3_) ppm.

#### 2‐(Bis(4‐methoxyphenyl)methyl)‐4‐chloroquinazoline (23)

Following the procedure described in ref. 27, starting from **16** (0.050 g, 0.13 mmol) and POCl_3_ (0.40 mL, 4.30 mmol), compound **23** (0.050 g, yield: 95.6 %) was obtained as a white solid.

Chromatographic eluent: CH_2_Cl_2_ and CH_2_Cl_2_/CH_3_OH/NH_4_OH 99 : 1:0.1. ^1^H‐NMR (400 MHz, CDCl_3_) δ: 8.20 (d, *J*=8.4 Hz, 1H, CH arom.), 8.02 (d, *J*=8.4 Hz, 1H, CH arom.), 7.89 (t, *J*=8.4 Hz, 1H, CH arom.), 7.64 (t, *J*=8.4 Hz, 1H, CH arom.), 7.37 (d, *J*=8.8 Hz, 4H, CH arom.), 6.86 (d, *J*=8.8 Hz, 4H, CH arom.), 5.77 (s, 1H, CH), 3.77 (s, 6H, OCH_3_) ppm.

#### General procedure for the synthesis of final compounds (1–7)

**Method A**:^[28]^ To a solution of the proper 4‐chloroquinazolines (1 equiv.) in the adequate amount of abs. ethanol, the suitable amine (1 equiv.) and methanesulfonic acid (5.0 μL) were added. The reaction mixture was refluxed for 4 h, then it was cooled to rt and the solvent was removed under reduced pressure. The residue was suspended into a 1 N NaOH solution and stirred for 1 h, then it was treated with CH_2_Cl_2_. The organic layer was washed twice with water and brine, dried over Na_2_SO_4_ and concentrated under vacuum. The desired derivatives were obtained as pure solids, or they were purified by flash chromatography using the proper eluting system. Final compounds were transformed into the corresponding hydrochloride as solid. The salts were crystallized from abs. ethanol/petroleum ether.

**Method B**: To a solution of the proper 4‐chloroquinazolines (1 equiv.) in the adequate amount of dry DMF, the suitable amine (1 equiv.) and K_2_CO_3_ (1 equiv.) were added. The mixture was heated at 60 °C for 5 h, then was cooled to rt. A proper amount of cold water was added: if a solid precipitated, it was filtrated and dried under vacuum. Otherwise, the mixture was extracted with CH_2_Cl_2_, and the organic phase was washed twice with brine, dried over Na_2_SO_4_ and concentrated under vacuum. The desired derivatives were obtained as pure solids, or they were purified by flash chromatography using the proper eluting system. Final compounds were transformed into the corresponding hydrochloride as solid. The salts were crystallized from abs. ethanol/petroleum ether.

#### (*E*)‐*N*‐(4‐(2‐(6,7‐Dimethoxy‐3,4‐dihydroisoquinolin‐2(1*H*)‐yl)ethyl)phenyl)‐2‐(3,4,5‐trimethoxystyryl)quinazolin‐4‐amine (1 a)

**Method A**: starting from the 4‐chloroquinazoline **17** (0.076 g, 0.21 mmol) and 4‐(2‐(6,7‐dimethoxy‐3,4‐dihydroisoquinolin‐2(1*H*)‐yl)ethyl)aniline^[30]^ (0.067 g, 0.21 mmol) in 4.0 mL of abs. ethanol, compound **1 a** (0.067 g, yield: 49.2 %) was synthesized as a yellow solid.

**Free base**: Chromatographic eluent: CH_2_Cl_2_/CH_3_OH/NH_4_OH 98 : 2:0.2. ^1^H‐NMR (400 MHz, CDCl_3_) δ: 7.88‐7.82 (m, 3H, CH arom. and C*H*=CH); 7.77 (d, *J*=8.4 Hz, 2H, CH arom.); 7.72 (t, *J*=7.6 Hz, 1H, CH arom.); 7.56 (bs, 1H, NH); 7.43 (t, *J*=7.6 Hz, 1H, CH arom.); 7.29 (d, *J*=8.4 Hz, 2H, CH arom.); 7.13 (d, *J*=16.0 Hz, 1H, C*H*=CH); 6.82 (s, 2H, CH arom.); 6.57 (s, 1H, CH arom.); 6.51 (s, 1H, CH arom.); 3.87 (s, 6H, OCH_3_); 3.85 (s, 3H, OCH_3_); 3.81 (s, 3H, OCH_3_); 3.80 (s, 3H, OCH_3_); 3.64 (s, 2H, CH_2_); 2.95–2.90 (m, 2H, CH_2_); 2.87‐2.73 (m, 6H, CH_2_) ppm. ^13^C‐NMR (100 MHz, CDCl_3_) δ: 160.6 (C); 157.0 (C); 153.4 (C); 150.8 (C); 147.6 (C); 147.3 (C); 138.9 (C); 137.6 (CH); 136.8 (C); 136.1 (C); 132.9 (CH); 132.2 (C); 129.1 (CH); 128.5 (CH); 128.4 (CH); 126.4 (C); 126.1 (C); 125.9 (CH); 121.5 (CH); 120.8 (CH); 114.1 (C); 111.4 (CH); 109.5 (CH); 104.8 (CH); 61.0 (CH_3_); 60.2 (CH_2_); 56.2 (CH_3_); 55.9 (CH_3_); 55.9 (CH_3_); 55.7 (CH_2_); 51.1 (CH_2_); 33.5 (CH_2_); 28.7 (CH_2_) ppm. ESI‐HRMS (*m/z*) calculated for [M+H]^+^ ion species C_38_H_41_N_4_O_5_= 633.3072, found 633.3073.

**Hydrochloride**: orange solid; mp 244–246 (dec) °C.

#### (*E*)‐*N*‐Phenethyl‐2‐(3,4,5‐trimethoxystyryl)quinazolin‐4‐amine (1 b)

**Method A**: starting from the 4‐chloroquinazoline **17** (0.10 g, 0.29 mmol) and 2‐phenylethanamine (0.040 mL, 0.29 mmol) in 5.0 mL of abs. ethanol, compound **1 b** (0.044 g, yield: 34.8 %) was synthesized as a pale‐yellow solid.

**Free base**: Chromatographic eluent: CH_2_Cl_2_/CH_3_OH 99 : 1. ^1^H‐NMR (400 MHz, CDCl_3_) δ: 7.95 (d, *J*=16.0 Hz, 1H, C*H*=CH); 7.78 (d, *J*=8.4 Hz, 1H, CH arom.); 7.66 (t, *J*=8.4 Hz, 1H, CH arom.); 7.52 (d, *J*=8.4 Hz, 1H, CH arom.); 7.34–7.24 (m, 6H, CH arom.); 7.14 (d, *J*=16.0 Hz, 1H, C*H*=CH); 6.86 (s, 2H, CH arom.); 5.90–5.78 (m, 1H, NH); 4.01 (q, *J*=6.8 Hz, 2H, CH_2_); 3.88 (s, 6H, OCH_3_); 3.86 (s, 3H, OCH_3_); 3.07 (t, *J*=6.8 Hz, 2H, CH_2_) ppm. ^13^C‐NMR (100 MHz, CDCl_3_) δ: 160.7 (C); 159.0 (C); 153.4 (C); 150.0 (C); 139.2 (C); 137.2 (CH); 132.7 (CH); 132.2 (C); 128.9 (CH); 128.7 (CH); 128.4 (CH); 128.1 (CH); 126.6 (CH); 125.4 (CH); 120.7 (CH); 113.9 (C); 104.6 (CH); 61.0 (CH_3_); 56.1 (CH_3_); 42.5 (CH_2_); 35.4 (CH_2_) ppm. ESI‐HRMS (*m/z*) calculated for [M+H]^+^ ion species C_27_H_28_N_3_O_3_=442.2125, found 442.2123.

**Hydrochloride**: yellow solid; mp 125–128 °C.

#### (*E*)‐4‐(2‐(3,4,5‐Trimethoxystyryl)quinazolin‐4‐yl)morpholine (1 c)

**Method A**: starting from the 4‐chloroquinazoline **17** (0.10 g, 0.29 mmol) and morpholine (0.025 mL, 0.29 mmol) in 5.0 mL of abs. ethanol, compound **1 c** (0.032 g, yield: 27.3 %) was synthesized as a pale‐yellow solid.

**Free base**: Chromatographic eluent: CH_2_Cl_2_/CH_3_OH 99 : 1. ^1^H‐NMR (400 MHz, CDCl_3_) δ: 7.89‐7.81 (m, 3H, CH arom. and C*H*=CH); 7.67 (t, *J*=8.0 Hz, 1H, CH arom.); 7.36 (t, *J*=8.0 Hz, 1H, CH arom.); 7.14 (d, *J*=15.6 Hz, 1H, C*H*=CH); 6.84 (s, 2H, CH arom.); 3.92‐3.90 (m, 4H, CH_2_); 3.87 (s, 6H, OCH_3_); 3.85 (s, 3H, OCH_3_); 3.78‐3.76 (m, 4H, CH_2_) ppm. ^13^C‐NMR (100 MHz, CDCl_3_) δ: 164.6 (C); 159.8 (C); 153.4 (C); 152.5 (C); 138.9 (C); 137.3 (CH); 132.7 (CH); 132.1 (C); 128.6 (CH); 128.1 (CH); 125.1 (CH); 124.8 (CH); 115.5 (C); 104.6 (CH); 66.8 (CH_2_); 61.0 (CH_3_); 56.1 (CH_3_); 50.4 (CH_2_) ppm. ESI‐HRMS (*m/z*) calculated for [M+H]^+^ ion species C_23_H_26_N_3_O_4_= 408.1918, found 408.1916.

**Hydrochloride**: yellow solid; mp 244–246 (dec) °C.

#### (*E*)‐4‐(4‐Methylpiperazin‐1‐yl)‐2‐(3,4,5‐trimethoxystyryl)quinazoline (1 d)

**Method A**: starting from the 4‐chloroquinazoline **17** (0.068 g, 0.19 mmol) and 1‐methylpiperazine (0.021 mL, 0.19 mmol) in 5.0 mL of ethanol, compound **1 d** (0.080 g, yield: 100.0 %) was synthesized as a pale‐yellow solid.

**Free base**: TLC: CH_2_Cl_2_/CH_3_OH 96 : 4. ^1^H‐NMR (400 MHz, CDCl_3_) δ: 7.83 (d, *J*=16.0 Hz, 1H, C*H*=CH); 7.78 (d, *J*=8.8 Hz, 2H, CH arom.); 7.61 (t, *J*=8.8 Hz, 1H, CH arom.); 7.30 (t, *J*=8.8 Hz, 1H, CH arom.); 7.10 (d, *J*=16.0 Hz, 1H, C*H*=CH); 6.81 (s, 2H, CH arom.); 3.83 (s, 6H, OCH_3_); 3.81 (s, 3H, OCH_3_); 3.77 (t, *J*=4.8 Hz, 4H, CH_2_); 2.58 (t, *J*=4.8 Hz, 4H, CH_2_); 2.31 (s, 3H, NCH_3_) ppm. ^13^C‐NMR (100 MHz, CDCl_3_) δ: 164.4 (C); 159.7 (C); 153.3 (C); 152.5 (C); 138.8 (C); 137.1 (CH); 132.4 (CH); 132.1 (C); 128.4 (CH); 128.3 (CH); 125.0 (CH); 124.8 (CH); 115.5 (C); 104.6 (CH); 60.9 (CH_3_); 56.1 (CH_3_); 55.0 (CH_2_); 49.6 (CH_2_); 46.2 (CH_3_) ppm. ESI‐HRMS (*m/z*) calculated for [M+H]^+^ ion species C_24_H_29_N_4_O_3_= 421.2234, found 421.2237.

**Hydrochloride**: yellow solid; mp 203–205 (dec) °C.

#### (*E*)‐4‐(6,7‐Dimethoxy‐3,4‐dihydroisoquinolin‐2(1*H*)‐yl)‐2‐(3,4,5‐trimethoxystyryl)quinazoline (1 e)

**Method B**: starting from the 4‐chloroquinazoline **17** (0.13 g, 0.36 mmol), 6,7‐dimethoxy‐1,2,3,4‐tetrahydroisoquinoline (0.071 g, 0.36 mmol) and K_2_CO_3_ (0.050 g, 0.36 mmol) in 4.0 mL of dry DMF, compound **1 e** (0.18 g, yield: 96.1 %) was synthesized as a yellow solid.

**Free base**: TLC: CH_2_Cl_2_/CH_3_OH 95 : 5. ^1^H‐NMR (400 MHz, CDCl_3_) δ: 7.98‐7.94 (m, 2H, CH arom. and C*H*=CH); 7.90 (d, *J*=7.6 Hz, 1H, CH arom.); 7.72 (t, *J*=7.6 Hz, 1H, CH arom.); 7.43 (t, *J*=7.6 Hz, 1H, CH arom.); 7.21 (d, *J*=16.0 Hz, 1H, C*H*=CH); 6.91 (s, 2H, CH arom.); 6.72 (s, 2H, CH arom.); 4.95 (s, 2H, NCH_2_Ar); 4.08 (t, *J*=5.4 Hz, 2H, CH_2_); 3.93 (s, 6H, OCH_3_); 3.89 (s, 9H, OCH_3_); 3.14 (t, *J*=5.4 Hz, 2H, CH_2_) ppm. ^13^C‐NMR (100 MHz, CDCl_3_) δ: 164.0 (C); 159.6 (C); 153.4 (C); 152.3 (C); 147.9 (C); 147.7 (C); 138.8 (C); 137.2 (CH); 132.5 (CH); 132.2 (C); 128.2 (CH); 126.5 (C); 125.7 (C); 125.0 (CH); 124.8 (CH); 115.5 (C); 111.6 (CH); 109.4 (CH); 104.6 (CH); 61.0 (CH_3_); 56.1 (CH_3_); 56.0 (CH_3_); 56.0 (CH_3_); 51.0 (CH_2_); 48.3 (CH_2_); 28.5 (CH_2_) ppm. ESI‐HRMS (*m/z*) calculated for [M+H]^+^ ion species C_30_H_32_N_3_O_5_= 514.2337, found 514.2333.

**Hydrochloride**: yellow solid; mp 228–231 (dec) °C.

#### *N*‐(4‐(2‐(6,7‐Dimethoxy‐3,4‐dihydroisoquinolin‐2(1*H*)‐yl)ethyl)phenyl)‐2‐(3,4,5‐trimethoxyphenyl) quinazolin‐4‐amine (2 a)

This compound was already described in ref. 28.

#### *N*‐Phenethyl‐2‐(3,4,5‐trimethoxyphenyl)quinazolin‐4‐amine (2 b)

**Method A**: starting from the 4‐chloroquinazoline **18** ^[28]^ (0.13 g, 0.39 mmol) and 2‐phenylethanamine (0.049 mL, 0.39 mmol) in 6.0 mL of abs. ethanol, compound **2 b** (0.077 g, yield: 47.7 %) was synthesized as a pale‐yellow solid.

**Free base**: Chromatographic eluent: CH_2_Cl_2_/CH_3_OH 99 : 1. ^1^H‐NMR (400 MHz, CDCl_3_) δ: 7.90 (s, 2H, CH arom.); 7.89 (d, *J*=7.6 Hz, 1H, CH arom.); 7.68 (t, *J*=7.6 Hz, 1H, CH arom.); 7.56 (d, *J*=7.6 Hz, 1H, CH arom.); 7.35 (t, *J*=7.6 Hz, 1H, CH arom.); 7.32‐7.21 (m, 5H, CH arom.); 5.85 (bs, 1H, NH); 4.04‐4.00 (m, 2H, CH_2_); 3.98 (s, 6H, OCH_3_); 3.91 (s, 3H, OCH_3_); 3.08 (t, *J*=7.2 Hz, 2H, CH_2_) ppm. ^13^C‐NMR (100 MHz, CDCl_3_) δ: 160.0 (C); 159.4 (C); 153.1 (C); 150.5 (C); 140.1 (C); 139.1 (C); 134.6 (C); 132.5 (CH); 128.8 (CH); 128.7 (CH); 126.6 (CH); 125.4 (CH); 120.4 (CH); 113.6 (C); 105.6 (CH); 60.9 (CH_3_); 56.2 (CH_3_); 42.5 (CH_2_); 35.4 (CH_2_) ppm. ESI‐HRMS (*m/z*) calculated for [M+H]^+^ ion species C_25_H_26_N_3_O_3_= 416.1969, found 416.1966.

**Hydrochloride**: pale yellow solid; mp 241–243 (dec) °C.

#### 4‐(2‐(3,4,5‐Trimethoxyphenyl)quinazolin‐4‐yl)morpholine (2 c)^[39]^

**Method A**: starting from the 4‐chloroquinazoline **18** ^[28]^ (0.12 g, 0.38 mmol) and morpholine (0.035 mL, 0.38 mmol) in 6.0 mL of abs. ethanol, compound **2 c** (0.064 g, yield: 43.9 %) was synthesized as a pale‐yellow solid.

**Free base**: Chromatographic eluent: CH_2_Cl_2_/CH_3_OH 99 : 1. ^1^H‐NMR (400 MHz, CDCl_3_) δ: 7.95 (d, *J*=8.0 Hz, 1H, CH arom.); 7.85 (d, *J*=8.0 Hz, 1H, CH arom.); 7.83 (s, 2H, CH arom.); 7.71 (t, *J*=8.0 Hz, 1H, CH arom.); 7.39 (t, *J*=8.0 Hz, 1H, CH arom.); 3.98 (s, 6H, OCH_3_); 3.94‐3.91 (m, 4H, CH_2_); 3.89 (s, 3H, OCH_3_); 3.81‐3.79 (m, 4H, CH_2_) ppm. ^13^C‐NMR (100 MHz, CDCl_3_) δ: 165.0 (C); 159.0 (C); 153.2 (C); 152.8 (C); 140.3 (C); 134.0 (C); 132.6 (CH); 129.1 (CH); 125.1 (CH); 124.6 (CH); 115.3 (C); 105.7 (CH); 66.8 (CH_2_); 60.9 (CH_3_); 56.2 (CH_3_); 50.4 (CH_2_) ppm. ESI‐HRMS (*m/z*) calculated for [M+H]^+^ ion species C_21_H_24_N_3_O_4_= 382.1761, found 382.1763.

**Hydrochloride**: pale yellow solid; mp 239–241 (dec) °C.

#### 4‐(4‐Methylpiperazin‐1‐yl)‐2‐(3,4,5‐trimethoxyphenyl)quinazoline (2 d)^[39]^

**Method A**: starting from the 4‐chloroquinazoline **18** ^[28]^ (0.10 g, 0.30 mmol) and 1‐methylpiperazine (0.035 mL, 0.30 mmol) in 10.0 mL of abs. ethanol, compound **2 d** (0.11 g, yield: 93.9 %) was synthesized as a pale‐yellow solid.

**Free base**: TLC: CH_2_Cl_2_/CH_3_OH 96 : 4. ^1^H‐NMR (400 MHz, CDCl_3_) δ: 7.88 (d, *J*=8.0 Hz, 1H, CH arom.); 7.81 (s, 2H, CH arom.); 7.79 (d, *J*=8.0 Hz, 1H, CH arom.); 7.62 (t, *J*=8.0 Hz, 1H, CH arom.); 7.31 (t, *J*=8.0 Hz, 1H, CH arom.); 3.93 (s, 6H, OCH_3_); 3.85 (s, 3H, OCH_3_); 3.82‐3.73 (m, 4H, CH_2_); 2.60‐2.51 (s, 4H, CH_2_); 2.30 (s, 3H, NCH_3_) ppm. ^13^C‐NMR (100 MHz, CDCl_3_) δ: 164.8 (C); 158.9 (C); 153.1 (C); 152.8 (C); 140.2 (C); 134.1 (C); 132.4 (CH); 128.9 (CH); 124.8 (CH); 124.8 (CH); 115.3 (C); 105.7 (CH); 60.9 (CH_3_); 56.2 (CH_3_); 54.9 (CH_2_); 49.7 (CH_2_); 46.2 (CH_3_) ppm. ESI‐HRMS (*m/z*) calculated for [M+H]^+^ ion species C_22_H_27_N_4_O_3_= 395.2078, found 395.2080.

**Hydrochloride**: yellow solid; mp 250–252 (dec) °C.

#### 4‐(6,7‐Dimethoxy‐3,4‐dihydroisoquinolin‐2(1*H*)‐yl)‐2‐(3,4,5‐trimethoxyphenyl)quinazoline (2 e)

**Method A**: starting from the 4‐chloroquinazoline **18** ^[28]^ (0.10 g, 0.32 mmol) and 6,7‐dimethoxy‐1,2,3,4‐tetrahydroisoquinoline (0.062 g, 0.32 mmol) in 4.0 mL of abs. ethanol, compound **2 e** (0.090 g, yield: 71.1 %) was synthesized as a pale‐yellow oil.

**Free base**: Chromatographic eluent: cyclohexane/EtOAc 50 : 50. TLC: CH_2_Cl_2_/CH_3_OH 98 : 2. ^1^H‐NMR (400 MHz, CDCl_3_) δ: 7.98 (d, *J*=7.6 Hz, 2H, CH arom.); 7.88 (s, 2H, CH arom.); 7.72 (t, *J=*7.6 Hz, 1H, CH arom.); 7.42 (t, *J*=7.6 Hz, 1H, CH arom.); 6.69 (s, 1H, CH arom.); 6.66 (s, 1H, CH arom.); 4.97 (s, 2H, NCH_2_Ar); 4.11 (t, *J=*5.4 Hz, 2H, CH_2_); 4.02 (s, 6H, OCH_3_); 3.92 (s, 3H, OCH_3_); 3.87 (s, 3H, OCH_3_); 3.86 (s, 3H, OCH_3_); 3.11 (t, *J*=5.4 Hz, 2H, CH_2_) ppm. ^13^C‐NMR (100 MHz, CDCl_3_) δ: 164.2 (C); 153.2 (C); 148.0 (C); 147.8 (C); 126.5 (C); 125.0 (CH); 111.6 (CH); 109.3 (CH); 106.1 (CH); 61.0 (CH_3_); 56.4 (CH_3_); 56.0 (CH_3_); 51.3 (CH_2_); 48.0 (CH_2_); 28.2 (CH_2_) ppm. ESI‐HRMS (*m/z*) calculated for [M+H]^+^ ion species C_28_H_30_N_3_O_5_= 488.2180, found 488.2179.

**Hydrochloride**: yellow solid; mp 155–158 (dec) °C.

#### 2‐(Anthracen‐9‐yl)‐*N*‐(4‐(2‐(6,7‐dimethoxy‐3,4‐dihydroisoquinolin‐2(1*H*)‐yl)ethyl)phenyl)quinazolin‐4‐amine (3 a)

**Method A**: starting from the 4‐chloroquinazoline **19** (0.090 g, 0.26 mmol) and 4‐(2‐(6,7‐dimethoxy‐3,4‐dihydroisoquinolin‐2(1*H*)‐yl)ethyl)aniline^[30]^ (0.082 g, 0.26 mmol) in 1.5 mL of abs. ethanol, compound **3 a** (0.040 g, yield: 24.6 %) was synthesized as a pale‐yellow solid.

**Free base**: Chromatographic eluent: CH_2_Cl_2_/2‐propanol/NH_4_OH 95 : 5:0.5. ^1^H‐NMR (400 MHz, CDCl_3_) δ: 8.47 (s, 1H, CH arom.); 8.05‐7.99 (m, 4H, CH arom.); 7.97 (bs, 1H, NH); 7.86 (d, *J*=8.0 Hz, 2H, CH arom.); 7.80 (t, *J*=8.0 Hz, 1H, CH arom.); 7.60 (d, *J*=8.4 Hz, 2H, CH arom.); 7.46 (t, *J*=8.0 Hz, 1H, CH arom.); 7.41 (t, *J*=8.0 Hz, 2H, CH arom.); 7.33 (t, *J*=8.0 Hz, 2H, CH arom.); 6.94 (d, *J*=8.4 Hz, 2H, CH arom.); 6.54 (s, 1H, CH arom.); 6.45 (s, 1H, CH arom.); 3.81 (s, 3H, OCH_3_); 3.78 (s, 3H, OCH_3_); 3.53 (s, 2H, CH_2_); 2.79‐2.64 (m, 6H, CH_2_); 2.61‐2.53 (m, 2H, CH_2_) ppm. ^13^C‐NMR (100 MHz, CDCl_3_) δ: 162.7 (C); 157.5 (C); 150.6 (C); 147.6 (C); 147.3 (C); 136.6 (C); 135.7 (C); 134.9 (C); 133.1 (CH); 131.5 (C); 129.7 (C); 129.0 (CH); 128.4 (CH); 127.6 (CH); 126.7 (CH); 126.3 (CH); 125.9 (C); 125.7 (CH); 125.0 (CH); 121.2 (CH); 120.9 (CH); 113.6 (C); 111.4 (CH); 109.5 (CH); 59.8 (CH_2_); 55.9 (CH_3_); 55.4 (CH_2_); 50.8 (CH_2_); 33.0 (CH_2_); 28.3 (CH_2_) ppm. ESI‐HRMS (*m/z*) calculated for [M+H]^+^ ion species C_41_H_37_N_4_O_2_= 617.2911, found 617.2905.

**Hydrochloride**: yellow solid; mp 285–288 (dec) °C.

#### 2‐(Anthracen‐9‐yl)‐*N*‐phenethylquinazolin‐4‐amine (3 b)

**Method B**: starting from the 4‐chloroquinazoline **19** (0.085 g, 0.25 mmol), 2‐phenylethanamine (0.031 mL, 0.25 mmol) and K_2_CO_3_ (0.034 g, 0.25 mmol) in 2.0 mL of dry DMF, compound **3 b** (0.070 g, yield: 66.3 %) was synthesized as a white solid.

**Free base**: mp 236–238 (dec) °C. Chromatographic eluent: CH_2_Cl_2_/CH_3_OH 99.5:0.5. ^1^H‐NMR (400 MHz, DMSO‐d_6_) δ: 8.70 (bs, 1H, NH); 8.68 (s, 1H, CH arom.); 8.39 (d, *J*=8.0 Hz, 1H, CH arom.); 8.13 (d, *J*=8.4 Hz, 2H, CH arom.); 7.83 (t, *J*=8.0 Hz, 1H, CH arom.); 7.74 (d, *J*=8.0 Hz, 1H, CH arom.); 7.70 (d, *J*=8.4 Hz, 2H, CH arom.); 7.61 (t, *J*=8.0 Hz, 1H, CH arom.); 7.49 (t, *J*=8.4 Hz, 2H, CH arom.); 7.40 (t, *J*=8.4 Hz, 2H, CH arom.); 7.16‐7.06 (m, 5H, CH, arom.); 3.67 (q, *J*=6.8 Hz, 2H, CH_2_); 2.93 (t, *J*=6.8 Hz, 2H, CH_2_) ppm. ^13^C‐NMR (100 MHz, DMSO‐d_6_) δ: 162.4 (C); 160.2 (C); 149.6 (C); 139.9 (C); 135.9 (C); 133.5 (CH); 131.3 (C); 129.2 (C); 129.1 (CH); 128.8 (CH); 128.7 (CH); 127.2 (CH); 126.5 (CH); 126.5 (CH); 126.3 (CH); 125.8 (CH); 123.3 (CH); 114.0 (C); 42.6 (CH_2_); 34.9 (CH_2_) ppm. ESI‐HRMS (*m/z*) calculated for [M+H]^+^ ion species C_30_H_24_N_3_= 426.1965, found 426.1963.

#### 4‐(2‐(Anthracen‐9‐yl)quinazolin‐4‐yl)morpholine (3 c)

**Method B**: starting from the 4‐chloroquinazoline **19** (0.052 g, 0.15 mmol), morpholine (0.013 mL, 0.15 mmol) and K_2_CO_3_ (0.021 g, 0.15 mmol) in 2.5 mL of dry DMF, compound **3 c** (0.060 g, yield: 100.0 %) was synthesized as a yellow solid.

**Free base**: Chromatographic eluent: CH_2_Cl_2_. ^1^H‐NMR (400 MHz, CDCl_3_) δ: 8.55 (s, 1H, CH arom.); 8.17‐8.08 (m, 1H, CH arom.); 8.05 (d, *J*=8.4 Hz, 3H, CH arom.); 7.84 (t, *J*=7.6 Hz, 1H, CH arom.); 7.76 (d, *J*=8.4 Hz, 2H, CH arom.); 7.59 (t, 7.6 Hz, 1H, CH arom.); 7.45 (t, *J*=8.4 Hz, 2H, CH arom.); 7.37 (t, *J*=8.4 Hz, 2H, CH arom.) 3.88 (s, 8H, CH_2_) ppm. ^13^C‐NMR (100 MHz, CDCl_3_) δ: 164.2 (C); 161.1 (C); 133.5 (CH); 131.4 (C); 130.1 (C); 129.8 (C); 128.6 (CH); 128.3 (CH); 126.1 (CH); 125.8 (CH); 125.1 (CH); 125.1 (CH); 124.8 (CH); 114.1 (C); 66.9 (CH_2_); 50.3 (CH_2_) ppm. ESI‐HRMS (*m/z*) calculated for [M+H]^+^ ion species C_26_H_22_N_3_O= 392.1757, found 392.1758.

**Hydrochloride**: yellow solid; mp 198–201 (dec) °C.

#### 2‐(Anthracen‐9‐yl)‐4‐(4‐methylpiperazin‐1‐yl)quinazoline (3 d)

**Method B**: starting from the 4‐chloroquinazoline **19** (0.060 g, 0.18 mmol), 1‐methylpiperazine (0.019 mL, 0.18 mmol) and K_2_CO_3_ (0.024 g, 0.18 mmol) in 3.0 mL of dry DMF, compound **3 d** (0.060 g, yield: 84.3 %) was synthesized as a pale‐yellow solid.

**Free base**: TLC: CH_2_Cl_2_/CH_3_OH/NH_4_OH 95 : 5:0.5. ^1^H‐NMR (400 MHz, CDCl_3_) δ: 8.52 (s, 1H, CH arom.); 8.07‐8.02 (m, 4H, CH arom.); 7.83‐7.78 (m, 3H, CH arom.); 7.54 (t, *J*=7.6 Hz, 1H, CH arom.); 7.44 (t, *J*=8.0 Hz, 2H, CH arom.); 7.37 (t, *J*=8.0 Hz, 2H, CH arom.); 3–90‐3.80 (m, 4H, CH_2_); 2.58 (t, *J*=4.0 Hz, 4H, CH_2_); 2.33 (s, 3H, CH_3_) ppm. ^13^C‐NMR (100 MHz, CDCl_3_) δ: 164.6 (C); 161.9 (C); 152.6 (C); 134.9 (C); 132.7 (CH); 131.6 (C); 129.8 (C); 129.1 (CH); 128.5 (CH); 127.6 (CH); 126.3 (CH); 125.8 (CH); 125.6 (CH); 125.0 (CH); 125.0 (CH); 114.9 (C); 55.0 (CH_2_); 49.7 (CH_2_); 46.1 (CH_3_) ppm. ESI‐HRMS (*m/z*) calculated for [M+H]^+^ ion species C_27_H_25_N_4_= 405.2074, found 405.2074.

**Hydrochloride**: orange solid; mp 278–280 (dec) °C.

#### 2‐(Anthracen‐9‐yl)‐4‐(6,7‐dimethoxy‐3,4‐dihydroisoquinolin‐2(1*H*)‐yl)quinazoline (3 e)

**Method B**: starting from the 4‐chloroquinazoline **19** (0.085 g, 0.25 mmol), 6,7‐dimethoxy‐1,2,3,4‐tetrahydroisoquinoline (0.048 g, 0.25 mmol) and K_2_CO_3_ (0.034 g, 0.25 mmol) in 2.0 mL of dry DMF, compound **3 e** (0.050 g, yield: 40.2 %) was synthesized as a pale‐yellow oil.

**Free base**: Chromatographic eluent: CH_2_Cl_2_/CH_3_OH 99.5:0.5. ^1^H‐NMR (400 MHz, CDCl_3_) δ: 8.53 (s, 1H, CH arom.); 8.15 (d, *J*=8.4 Hz, 1H, CH arom.); 8.08 (d, *J*=8.4 Hz, 1H, CH arom.); 8.04 (d, *J*=8.4 Hz, 2H, CH arom.); 7.84‐7.99 (m, 3H, CH arom.); 7.58 (t, *J*=8.4 Hz, 1H, CH arom.); 7.44 (t, *J*=7.2 Hz, 2H, CH arom.); 7.35 (t, *J*=7.2 Hz, 2H, CH arom.); 6.66 (s, 1H, CH arom.); 6.57 (s, 1H, CH arom.); 4.94 (s, 2H, NCH_2_Ar); 4.11 (t, *J*=5.6 Hz, 2H, CH_2_); 3.86 (s, 3H, OCH_3_); 3.77 (s, 3H, OCH_3_); 3.04 (t, *J*=5.6 Hz, 2H, CH_2_) ppm. ^13^C‐NMR (100 MHz, CDCl_3_) δ: 163.8 (C); 161.8 (C); 152.3 (C); 147.9 (C); 147.8 (C); 134.8 (C); 132.7 (CH); 131.6 (C); 129.8 (C); 128.8 (CH); 128.5 (CH); 127.6 (CH); 126.4 (C); 126.3 (CH); 125.8 (CH); 125.6 (C); 125.4 (CH); 125.1 (CH); 114.7 (C); 111.5 (CH); 109.3 (CH); 56.0 (CH_3_); 55.9 (CH_3_); 51.1 (CH_2_); 48.2 (CH_2_); 28.6 (CH_2_) ppm. ESI‐HRMS (*m/z*) calculated for [M+H]^+^ ion species C_33_H_28_N_3_O_2_= 498.2176, found 498.2176.

**Hydrochloride**: yellow solid; mp 210–213 (dec) °C.

#### *N*‐(4‐(2‐(6,7‐Dimethoxy‐3,4‐dihydroisoquinolin‐2(1*H*)‐yl)ethyl)phenyl)‐2‐(2,3,4‐trimethoxyphenyl)quinazolin‐4‐amine (4 a).

**Method A**: starting from the 4‐chloroquinazoline **20** (0.080 g, 0.24 mmol) and 4‐(2‐(6,7‐dimethoxy‐3,4‐dihydroisoquinolin‐2(1*H*)‐yl)ethyl)aniline^[30]^ (0.076 g, 0.24 mmol) in 2.0 mL of abs. ethanol **4 a** (0.070 g, yield: 47.5 %) was recrystallized from ethanol, as an orange solid.

**Free base**: TLC: CH_2_Cl_2_/CH_3_OH/NH_4_OH 95 : 5:0.5. ^1^H‐NMR (400 MHz, CDCl_3_) δ: 7.97‐7.88 (m, 2H, CH arom.); 7.79–7.54 (m, 4H, CH arom.); 7.46–7.34 (m, 1H, CH arom.); 7.25–7.12 (m, 2H, CH arom.); 6.75 (d, *J*=8.8 Hz, 1H, CH arom.); 6.58 (s, 1H, CH arom.); 6.52 (s, 1H, CH arom.); 3.87 (s, 6H, OCH_3_); 3.82 (s, 6H, OCH_3_); 3.81 (s, 3H, OCH_3_); 3.63 (s, 2H, CH_2_); 3.90‐2.70 (m, 8H, CH_2_) ppm. ^13^C‐NMR (100 MHz, CDCl_3_) δ: 161.1 (C); 157.3 (C); 154.7 (C); 153.2 (C); 150.9 (C); 147.5 (C); 147.2 (C); 142.7 (C); 136.9 (C); 136.0 (C); 132.7 (CH); 130.3 (CH); 129.1 (CH); 129.0 (CH); 127.5 (C); 126.5 (C); 126.3 (CH); 126.1 (C); 126.0 (CH); 121.7 (CH); 120.6 (CH); 113.4 (C); 111.4 (CH); 109.5 (CH); 107.2 (CH); 61.7 (CH_3_); 61.0 (CH_3_); 60.2 (CH_2_); 56.1 (CH_3_); 55.9 (CH_3_); 55.7 (CH_2_); 51.1 (CH_2_); 50.6 (CH_3_); 33.4 (CH_2_); 28.6 (CH_2_) ppm. ESI‐HRMS (*m/z*) calculated for [M+H]^+^ ion species C_36_H_39_N_4_O_5_= 607.2915, found 607.2915.

**Hydrochloride**: orange solid; mp 110–113 (dec) °C.

#### *N*‐Phenethyl‐2‐(2,3,4‐trimethoxyphenyl)quinazolin‐4‐amine (4 b).

**Method B**: starting from the 4‐chloroquinazoline **20** (0.11 g, 0.33 mmol), 2‐phenylethanamine (0.042 mL, 0.33 mmol) and K_2_CO_3_ (0.046 g, 0.33 mmol) in 4.0 mL of dry DMF, compound **4 b** (0.030 g, yield: 21.7 %) was synthesized as a pale‐yellow oil.

**Free base**: Chromatographic eluent: CH_2_Cl_2_/CH_3_OH 97 : 3. ^1^H‐NMR (400 MHz, CDCl_3_) δ: 7.85 (d, *J=*7.6 Hz, 1H, CH arom.); 7.73–7.67 (m, 3H, CH arom.); 7.39 (t, *J*=7.6 Hz, 1H, CH arom.); 7.33–7.23 (m, 5H, CH arom.); 6.79 (d, *J*=8.8 Hz, 1H, CH arom.); 6.35 (bs, 1H, NH); 4.02–3.95 (m, 5H, CH_2_ and OCH_3_); 3.92 (s, 6H, OCH_3_); 3.06 (t, *J*=6.8 Hz, 2H, CH_2_) ppm. ^13^C‐NMR (100 MHz, CDCl_3_) δ: 161.0 (C); 159.3 (C); 154.9 (C); 153.0 (C); 142.7 (C); 139.1 (C); 132.6 (CH); 128.9 (CH); 128.7 (CH); 127.9 (CH); 126.5 (CH); 126.3 (CH); 125.7 (CH); 120.9 (CH); 113.2 (C); 107.4 (CH); 61.8 (CH_3_); 61.0 (CH_3_); 56.1 (CH_3_); 42.5 (CH_2_); 35.4 (CH_2_) ppm. ESI‐HRMS (*m/z*) calculated for [M+H]^+^ ion species C_25_H_26_N_3_O_3_= 416.1969, found 416.1967.

**Hydrochloride**: white solid; mp 165–168 (dec) °C.

#### 4‐(2‐(2,3,4‐Trimethoxyphenyl)quinazolin‐4‐yl)morpholine (4 c)

**Method B**: starting from the 4‐chloroquinazoline **20** (0.090 g, 0.27 mmol), morpholine (0.024 mL, 0.27 mmol) and K_2_CO_3_ (0.038 g, 0.27 mmol) in 4.0 mL of dry DMF, compound **4 c** (0.080 g, yield: 77.2 %) was synthesized as a yellow oil.

**Free base**: Chromatographic eluent: CH_2_Cl_2_/CH_3_OH 99 : 1. ^1^H‐NMR (400 MHz, CDCl_3_) δ: 8.02 (d, *J*=8.0 Hz, 1H, CH arom.); 7.88 (d, *J*=8.0 Hz, 1H, CH arom.); 7.72 (t, *J*=8.0 Hz, 1H, CH arom.); 7.65 (d, *J*=8.8 Hz, 1H, CH arom.); 7.43 (t, *J*=8.0 Hz, 1H, CH arom.); 6.78 (d, *J*=8.8 Hz, 1H, CH arom.); 3.98 (s, 3H, OCH_3_); 3.94–3.88 (m, 10H, CH_2_ and OCH_3_); 3.83–3.79 (m, 4H, CH_2_) ppm. ^13^C‐NMR (100 MHz, CDCl_3_) δ: 164.6 (C); 160.0 (C); 154.9 (C); 153.1 (C); 152.1 (C); 142.8 (C); 132.6 (CH); 128.7 (CH); 126.4 (CH); 125.2 (CH); 124.5 (CH); 114.6 (C); 107.4 (CH); 66.9 (CH_2_); 61.7 (CH_3_); 61.0 (CH_3_); 56.1 (CH_3_); 50.4 (CH_2_) ppm. ESI‐HRMS (*m/z*) calculated for [M+H]^+^ ion species C_21_H_24_N_3_O_4_= 382.1761, found 382.1761.

**Hydrochloride**: orange solid; mp 176–178 °C.

#### 4‐(4‐Methylpiperazin‐1‐yl)‐2‐(2,3,4‐trimethoxyphenyl)quinazoline (4 d)

**Method A**: starting from the 4‐chloroquinazoline **20** (0.060 g, 0.18 mmol) and 1‐methylpiperazine (0.020 mL, 0.18 mmol) in 4.0 mL of abs. ethanol, compound **4 d** (0.027 g, yield: 37.7 %) was synthesized as a yellow oil.

**Free base**: Chromatographic eluent: CH_2_Cl_2_/CH_3_OH/NH_4_OH 95 : 5:0.5. ^1^H‐NMR (400 MHz, CDCl_3_) δ: 7.95 (d, *J*=8.0 Hz, 1H, CH arom.); 7.87 (d, *J*=8.0 Hz, 1H, CH arom.); 7.71 (t, *J*=8.0 Hz, 1H, CH arom.); 7.64 (d, *J*=8.8 Hz, 1H, CH arom.); 7.42 (t, *J*=8.0 Hz, 1H, CH arom.); 6.78 (d, *J*=8.8 Hz, 1H, CH arom.); 3.96 (s, 3H, OCH_3_); 3.95–3.91 (m, 4H, CH_2_); 3.90 (s, 6H, OCH_3_); 2.84–2.71 (m, 4H, CH_2_); 2.46 (s, 3H, CH_3_) ppm. ^13^C‐NMR (100 MHz, CDCl_3_) δ: 164.4 (C); 160.1 (C); 154.8 (C); 153.0 (C); 152.6 (C); 142.8 (C); 132.5 (CH); 129.0 (CH); 127.1 (C); 126.3 (CH); 125.2 (CH); 124.6 (CH); 114.8 (C); 107.4 (CH); 61.7 (CH_3_); 61.0 (CH_3_); 56.1 (CH_3_); 54.5 (CH_2_); 49.0 (CH_2_); 45.6 (CH_3_) ppm. ESI‐HRMS (*m/z*) calculated for [M+H]^+^ ion species C_22_H_27_N_4_O_3_= 395.2078, found 395.2075.

**Hydrochloride**: yellow solid; mp 247–249 °C.

#### 4‐(6,7‐Dimethoxy‐3,4‐dihydroisoquinolin‐2(1*H*)‐yl)‐2‐(2,3,4‐trimethoxyphenyl)quinazoline (4 e)

**Method B**: starting from the 4‐chloroquinazoline **20** (0.090 g, 0.27 mmol), 6,7‐dimethoxy‐1,2,3,4‐tetrahydroisoquinoline (0.053 g, 0.27 mmol) and K_2_CO_3_ (0.038 g, 0.27 mmol) in 4.0 mL of dry DMF, compound **4 e** (0.11 g, yield: 83.1 %) was synthesized as a yellow solid.

**Free base**: TLC: CH_2_Cl_2_/CH_3_OH/NH_4_OH 95 : 5:0.5. ^1^H‐NMR (400 MHz, CDCl_3_) δ: 8.05‐7.94 (m, 2H, CH arom.); 7.73 (t, *J*=7.2 Hz, 1H, CH arom.); 7.67 (d, *J*=8.4 Hz, 1H, CH arom.); 7.46 (t, *J*=7.2 Hz, 1H, CH arom.); 6.80 (d, *J*=8.4 Hz, 1H, CH arom.); 6.70 (s, 1H, CH arom.); 6.65 (s, 1H, CH arom.); 4.93 (s, 2H, NCH_2_Ar); 4.12–4.02 (m, 2H, CH_2_); 3.99 (s, 3H, OCH_3_); 3.92 (s, 6H, OCH_3_); 3.88 (s, 3H, OCH_3_); 3.86 (s, 3H, OCH_3_); 3.17‐3.07 (m, 2H, CH_2_) ppm. ^13^C‐NMR (100 MHz, CDCl_3_) δ: 164.1 (C); 160.2 (C); 154.7 (C); 153.0 (C); 152.6 (C); 147.8 (C); 147.7 (C); 142.8 (C); 132.2 (CH); 128.8 (CH); 127.5 (C); 126.5 (C); 126.3 (CH); 125.8 (C); 124.8 (CH); 124.7 (CH); 114.8 (C); 111.6 (CH); 109.3 (CH); 107.3 (CH); 61.8 (CH_3_); 61.0 (CH_3_); 56.1 (CH_3_); 56.0 (CH_3_); 51.1 (CH_2_); 48.4 (CH_2_); 28.6 (CH_2_) ppm. ESI‐HRMS (*m/z*) calculated for [M+H]^+^ ion species C_28_H_30_N_3_O_5_=488.2180, found 488.2184.

**Hydrochloride**: orange solid; mp 94–96 °C.

#### *N*‐(4‐(2‐(6,7‐Dimethoxy‐3,4‐dihydroisoquinolin‐2(1*H*)‐yl)ethyl)phenyl)‐2‐(2‐methoxynaphthalen‐1‐yl)quinazolin‐4‐amine (5 a)

**Method A**: starting from the 4‐chloroquinazoline **21** (0.090 g, 0.28 mmol) and 4‐(2‐(6,7‐dimethoxy‐3,4‐dihydroisoquinolin‐2(1*H*)‐yl)ethyl)aniline^[30]^ (0.088 g, 0.28 mmol) in 3.0 mL of abs. ethanol, compound **5 a** (0.080 g, yield: 47.7 %) was synthesized as a yellow oil.

**Free base**: Chromatographic eluent: CH_2_Cl_2_/CH_3_COCH_3_/NH_4_OH 80 : 20:0.2. ^1^H‐NMR (400 MHz, CDCl_3_) δ: 7.99‐7.91 (m, 3H, NH and CH arom.); 7.86 (d, *J*=8.8 Hz, 1H, CH arom.); 7.79 (d, *J*=8.8 Hz, 1H, CH arom.); 7.72 (t, *J*=7.6 Hz, 1H, CH arom.); 7.60–7.56 (m, 3H, CH arom.); 7.38–7.26 (m, 4H, CH arom.); 6.95 (d, *J*=8.4 Hz, 2H, CH arom.); 6.55 (s, 1H, CH arom.); 6.47 (s, 1H, CH arom.); 3.82 (s, 3H, OCH_3_); 3.81 (s, 3H, OCH_3_); 3.79 (s, 3H, OCH_3_); 3.55 (s, 2H, NCH_2_Ar); 2.82‐2.76 (m, 2H, CH_2_); 2.75‐2.66 (m, 4H, CH_2_); 2.62‐2.52 (m, 2H, CH_2_) ppm. ^13^C‐NMR (100 MHz, CDCl_3_) δ: 161.1 (C); 157.7 (C); 154.4 (C); 150.7 (C); 147.5 (C); 147.2 (C); 136.8 (C); 135.7 (C); 133.0 (C); 132.7 (CH); 130.0 (CH); 129.0 (C); 128.9 (CH); 127.9 (CH); 126.6 (CH); 126.5 (C); 126.2 (CH); 126.1 (C); 124.8 (CH); 124.3 (C); 123.5 (CH); 121.2 (CH); 121.1 (CH); 113.8 (C); 113.8 (CH); 111.3 (CH); 109.4 (CH); 60.1 (CH_2_); 56.6 (CH_3_); 55.9 (CH_3_); 55.6 (CH_2_); 51.0 (CH_2_); 33.3 (CH_2_); 28.6 (CH_2_) ppm. ESI‐HRMS (*m/z*) calculated for [M+H]^+^ ion species C_38_H_37_N_4_O_3_= 597.2860, found 597.2856.

**Hydrochloride**: orange solid; mp 215–218 (dec) °C.

#### 2‐(2‐Methoxynaphthalen‐1‐yl)‐*N*‐phenethylquinazolin‐4‐amine (5 b)

**Method B**: starting from the 4‐chloroquinazoline **21** (0.10 g, 0.31 mmol), 2‐phenylethanamine (0.040 mL, 0.31 mmol) and K_2_CO_3_ (0.043 g, 0.31 mmol) in 4.0 mL of dry DMF, compound **5 b** (0.11 g, yield: 86.9 %) was synthesized as a yellow solid.

**Free base**: TLC: CH_2_Cl_2_/CH_3_OH/NH_4_OH 98 : 2:0.2. ^1^H‐NMR (400 MHz, CDCl_3_) δ: 7.94 (d, *J=*8.4 Hz, 1H, CH arom.); 7.88 (d, *J*=8.8 Hz, 1H, CH arom.); 7.82–7.77 (m, 1H, CH arom.); 7.76–7.64 (m, 2H, CH arom.); 7.58–7.50 (m, 1H, CH arom.); 7.43 (t, *J*=7.6 Hz, 1H, CH arom.); 7.39–7.24 (m, 5H, CH arom.); 7.22 (d, *J*=6.8 Hz, 1H, CH arom.); 7.17 (d, *J*=7.2 Hz, 2H, CH arom.); 6.14 (bs, 1H, NH); 3.93‐3.81 (m, 5H, CH_2_ and OCH_3_); 2.99 (t, *J*=6.8 Hz, 2H, CH_2_) ppm. ^13^C‐NMR (100 MHz, CDCl_3_) δ: 161.6 (C); 159.7 (C); 154.4 (C); 149.9 (C); 139.2 (C); 133.0 (C); 132.5 (CH); 129.9 (CH); 129.1 (C); 128.9 (CH); 128.6 (CH); 128.5 (CH); 127.9 (CH); 126.5 (CH); 126.4 (CH); 125.8 (CH); 124.9 (CH); 124.8 (C); 123.5 (CH); 120.8 (CH); 114.1 (CH); 113.5 (C); 56.9 (CH_3_); 42.4 (CH_2_); 35.1 (CH_2_) ppm. ESI‐HRMS (*m/z*) calculated for [M+H]^+^ ion species C_27_H_24_N_3_O= 406.1914, found 406.1917.

**Hydrochloride**: yellow solid; mp 232–234 (dec) °C.

#### 4‐(2‐(2‐Methoxynaphthalen‐1‐yl)quinazolin‐4‐yl)morpholine (5 c)

**Method B**: starting from the 4‐chloroquinazoline **21** (0.080 g, 0.25 mmol), morpholine (0.022 mL, 0.25 mmol) and K_2_CO_3_ (0.034 g, 0.25 mmol) in 3.5 mL of dry DMF, compound **5 c** (0.090 g, yield: 97.3 %) was synthesized as a yellow solid.

**Free base**: TLC: CH_2_Cl_2_/CH_3_OH 95 : 5. ^1^H‐NMR (400 MHz, CDCl_3_) δ: 8.06 (d, *J*=8.4 Hz, 1H, CH arom.); 7.98 (d, *J*=8.4 Hz, 1H, CH arom.); 7.91 (d, *J*=8.8 Hz, 1H, CH arom.); 7.82–7.75 (m, 2H, CH arom.); 7.51 (t, *J*=7.2 Hz, 1H, CH arom.); 7.47–7.43 (m, 1H, CH arom.); 7.38 (d, *J*=8.8 Hz, 1H, CH arom.); 7.35–7.28 (m, 2H, CH arom.); 3.92–3.85 (m, 7H, CH_2_ and OCH_3_); 3.84–3.77 (m, 4H, CH_2_) ppm. ^13^C‐NMR (100 MHz, CDCl_3_) δ: 164.9 (C); 160.3 (C); 154.6 (C); 132.9 (C); 132.6 (CH); 130.2 (CH); 129.1 (C); 129.0 (CH); 127.9 (CH); 126.6 (CH); 125.5 (CH); 124.7 (CH); 123.6 (CH); 115.0 (C); 114.2 (CH); 66.9 (CH_2_); 57.1 (CH_3_); 50.4 (CH_2_) ppm. ESI‐HRMS (*m/z*) calculated for [M+H]^+^ ion species C_23_H_22_N_3_O_2_= 372.1707, found 372.1709.

**Hydrochloride**: yellow solid; mp 244–246 (dec) °C.

#### 2‐(2‐Methoxynaphthalen‐1‐yl)‐4‐(4‐methylpiperazin‐1‐yl)quinazoline (5 d).

**Method B**: starting from the 4‐chloroquinazoline **21** (0.11 g, 0.33 mmol), 1‐methylpiperazine (0.036 mL, 0.33 mmol) and K_2_CO_3_ (0.045 g, 0.33 mmol) in 4.0 mL of dry DMF, compound **5 d** (0.12 g, yield: 95.4 %) was synthesized as a yellow oil.

**Free base**: Chromatographic eluent: CH_2_Cl_2_/CH_3_OH/NH_4_OH 98 : 2:0.2. ^1^H‐NMR (400 MHz, CDCl_3_) δ: 7.99 (t, *J*=8.4 Hz, 2H, CH arom.); 7.90 (d, *J*=8.8 Hz, 1H, CH arom.); 7.83–7.78 (m, 1H, CH arom.); 7.75 (t, *J*=8.4 Hz, 1H, CH arom.); 7.53–7.43 (m, 2H, CH arom.); 7.38 (d, *J*=8.8 Hz, 1H, CH arom.); 7.35–7.27 (m, 2H, CH arom.); 3.88 (s, 3H, OCH_3_); 3.85 (t, *J*=4.8 Hz, 4H, CH_2_); 2.62 (t, *J*=4.8 Hz, 4H, CH_2_); 2.37 (s, 3H, CH_3_) ppm. ^13^C‐NMR (100 MHz, CDCl_3_) δ: 164.9 (C); 160.4 (C); 154.6 (C); 152.6 (C); 133.0 (C); 132.4 (CH); 130.0 (CH); 129.2 (C); 129.0 (CH); 127.9 (CH); 126.5 (CH); 125.2 (CH); 124.9 (CH); 124.8 (CH); 124.6 (C); 123.6 (CH); 115.1 (C); 114.4 (CH); 57.1 (CH_3_); 55.0 (CH_2_); 49.7 (CH_2_); 46.2 (CH_3_) ppm. ESI‐HRMS (*m/z*) calculated for [M+H]^+^ ion species C_24_H_25_N_4_O= 385.2023, found 385.2025.

**Hydrochloride**: yellow solid; mp 135–138 °C.

#### 4‐(6,7‐Dimethoxy‐3,4‐dihydroisoquinolin‐2(1*H*)‐yl)‐2‐(2‐methoxynaphthalen‐1‐yl)quinazoline (5 e)

**Method B**: starting from the 4‐chloroquinazoline **21** (0.083 g, 0.26 mmol), 6,7‐dimethoxy‐1,2,3,4‐tetrahydroisoquinoline (0.050 g, 0.26 mmol) and K_2_CO_3_ (0.036 g, 0.26 mmol) in 3.0 mL of dry DMF, compound **5 e** (0.090 g, yield: 73.6 %) was synthesized as a yellow solid.

**Free base**: TLC: CH_2_Cl_2_/CH_3_OH/NH_4_OH 95 : 5:0.5. ^1^H‐NMR (400 MHz, CDCl_3_) δ: 8.05 (d, *J*=8.4 Hz, 1H, CH arom.); 8.02 (d, *J*=8.4 Hz, 1H, CH arom.); 7.89 (d, *J*=9.2 Hz, 1H, CH arom.); 7.83–7.76 (m, 1H, CH arom.); 7.75 (t, *J*=8.4 Hz, 1H, CH arom.); 7.50 (t, *J*=8.4 Hz, 1H, CH arom.); 7.47–7.42 (m, 1H, CH arom.); 7.37 (d, *J*=9.2 Hz, 1H, CH arom.); 7.36–7.27 (m, 2H, CH arom.); 6.66 (s, 1H, CH arom.); 6.59 (s, 1H, CH arom.); 4.89 (s, 2H, NCH_2_Ar); 4.05 (t, *J*=5.6 Hz, 2H, CH_2_); 3.87 (s, 3H, OCH_3_); 3.85 (s, 3H, OCH_3_); 3.78 (s, 3H, OCH_3_); 3.06 (t, *J*=5.6 Hz, 2H, CH_2_) ppm. ^13^C‐NMR (100 MHz, CDCl_3_) δ: 164.1 (C); 160.2 (C); 154.6 (C); 152.4 (C); 147.9 (C); 147.8 (C); 133.0 (C); 132.3 (CH); 130.0 (CH); 129.2 (C); 128.7 (CH); 127.9 (CH); 126.5 (CH); 125.8 (C); 125.1 (CH); 124.9 (CH); 123.5 (CH); 114.9 (C); 114.4 (CH); 111.6 (CH); 109.4 (CH); 57.1 (CH_3_); 56.0 (CH_3_); 56.0 (CH_3_); 51.1 (CH_2_); 48.3 (CH_2_); 28.6 (CH_2_) ppm. ESI‐HRMS (*m/z*) calculated for [M+H]^+^ ion species C_30_H_28_N_3_O_3_= 478.2125, found 478.2126.

**Hydrochloride**: yellow solid; mp 184–186 (dec) °C.

#### *N*‐(4‐(2‐(6,7‐Dimethoxy‐3,4‐dihydroisoquinolin‐2(1*H*)‐yl)ethyl)phenyl)‐2‐(2,3‐dimethoxynaphthalen‐1‐yl)quinazolin‐4‐amine 6 a)

**Method A**: starting from the 4‐chloroquinazoline **22** (0.080 g, 0.23 mmol) and 4‐(2‐(6,7‐dimethoxy‐3,4‐dihydroisoquinolin‐2(1*H*)‐yl)ethyl)aniline^[30]^ (0.071 g, 0.23 mmol) in 3.0 mL of abs. ethanol, compound **6 a** (0.030 g, yield: 21.0 %) was synthesized as a yellow oil.

**Free base**: Chromatographic eluent: CH_2_Cl_2_/CH_3_COCH_3_/NH_4_OH 80 : 20:0.2. ^1^H‐NMR (400 MHz, CDCl_3_) δ: 8.04 (d, *J*=8.0 Hz, 1H, CH arom.); 7.97 (d, *J*=8.0 Hz, 1H, CH arom.); 7.87 (bs, 1H, NH); 7.77 (t, *J*=8.0 Hz, 1H, CH arom.); 7.72 (d, *J*=8.0 Hz, 1H, CH arom.); 7.66 (d, *J*=8.0 Hz, 2H, CH arom.); 7.53–7.47 (m, 2H, CH arom.); 7.34 (t, *J*=8.0 Hz, 1H, CH arom.); 7.23‐7.19 (m, 2H, CH arom.); 7.02 (d, *J*=8.0 Hz, 2H, CH arom.); 6.56 (s, 1H, CH arom.); 6.48 (s, 1H, CH arom.); 3.98 (s, 3H, OCH_3_); 3.83 (s, 3H, OCH_3_); 3.82 (s, 3H, OCH_3_); 3.80 (s, 3H, OCH_3_); 3.59 (s, 2H, NCH_2_Ar); 2.82–2.70 (m, 6H, CH_2_); 2.67–2.61 (m, 2H, CH_2_) ppm. ^13^C‐NMR (100 MHz, CDCl_3_) δ: 160.7 (C); 157.5 (C); 152.2 (C); 150.5 (C); 147.6 (C); 147.3 (C); 147.1 (C); 136.8 (C); 135.5 (C); 132.8 (CH); 131.4 (C); 131.1 (C); 129.0 (CH); 127.8 (C); 126.5 (CH); 126.5 (CH); 125.9 (C); 125.3 (CH); 125.2 (CH); 124.2 (CH); 121.4 (CH); 120.9 (CH); 113.8 (C); 111.3 (CH); 109.4 (CH); 107.6 (CH); 61.7 (CH_3_); 59.8 (CH_2_); 55.9 (CH_3_); 55.7 (CH_3_); 55.4 (CH_2_); 50.9 (CH_2_); 33.1 (CH_2_); 28.3 (CH_2_) ppm. ESI‐HRMS (*m/z*) calculated for [M+H]^+^ ion species C_39_H_39_N_4_O_4_= 627.2966, found 627.2971.

**Hydrochloride**: yellow solid; mp 256–259 (dec) °C.

#### 2‐(2,3‐Dimethoxynaphthalen‐1‐yl)‐*N*‐phenethylquinazolin‐4‐amine (6 b)

**Method B**: starting from the 4‐chloroquinazoline **22** (0.070 g, 0.20 mmol), 2‐phenylethanamine (0.025 mL, 0.20 mmol) and K_2_CO_3_ (0.028 g, 0.20 mmol) in 3.0 mL of dry DMF, compound **6 b** (0.060 g, yield: 69.0 %) was synthesized as a white solid.

**Free base**: TLC: CH_2_Cl_2_/CH_3_OH/NH_4_OH 98 : 2:0.2. ^1^H‐NMR (400 MHz, CDCl_3_) δ: 7.93 (d, *J*=8.4 Hz, 1H, CH arom.); 7.76–7.71 (m, 2H, CH arom.); 7.66 (d, *J*=8.0 Hz, 1H, CH arom.); 7.50–7.41 (m, 2H, CH arom.); 7.35 (t, *J*=8.0 Hz, 1H, CH arom.); 7.31–7.15 (m, 7H, CH arom.); 6.02 (bs, 1H, NH); 4.01 (s, 3H, OCH_3_); 3.94‐3.88 (m, 5H, CH_2_ and OCH_3_); 2.98 (t, *J*=6.8 Hz, 2H, CH_2_) ppm. ^13^C‐NMR (100 MHz, CDCl_3_) δ: 161.1 (C); 159.6 (C); 152.2 (C); 149.6 (C); 146.9 (C); 139.0 (C); 132.6 (CH); 131.5 (C); 131.2 (C); 128.9 (CH); 128.7 (CH); 128.4 (CH); 127.8 (C); 126.6 (CH); 126.5 (CH); 126.0 (CH); 125.3 (CH); 125.1 (CH); 124.1 (CH); 120.7 (CH); 113.5 (C); 107.7 (CH); 61.7 (CH_3_); 55.8 (CH_3_); 42.3 (CH_2_); 35.3 (CH_2_) ppm. ESI‐HRMS (*m/z*) calculated for [M+H]^+^ ion species C_28_H_26_N_3_O_2_= 436.2020, found 436.2016.

**Hydrochloride**: white solid; mp 256–259 (dec) °C.

#### 4‐(2‐(2,3‐Dimethoxynaphthalen‐1‐yl)quinazolin‐4‐yl)morpholine (6 c).

**Method B**: starting from the 4‐chloroquinazoline **22** (0.070 g, 0.20 mmol), morpholine (0.017 mL, 0.20 mmol) and K_2_CO_3_ (0.028 g, 0.20 mmol) in 3.0 mL of dry DMF, compound **6 c** (0.070 g, yield: 87.2 %) was synthesized as a white solid.

**Free base**: TLC: CH_2_Cl_2_/CH_3_OH 95 : 5. ^1^H‐NMR (400 MHz, CDCl_3_) δ: 8.03 (d, *J*=8.0 Hz, 1H, CH arom.); 7.98 (d, *J*=8.0 Hz, 1H, CH arom.); 7.77 (t, *J*=8.0 Hz, 1H, CH arom.); 7.73 (d, *J*=8.0 Hz, 1H, CH arom.); 7.51 (t, *J*=8.0 Hz, 1H, CH arom.); 7.42 (d, *J*=8.0 Hz, 1H, CH arom.); 7.35 (t, *J*=8.0 Hz, 1H, CH arom.); 7.26 (s, 1H, CH arom.); 7.22 (t, *J*=8.0 Hz, 1H, CH arom.); 4.01 (s, 3H, OCH_3_); 3.90 (s, 3H, OCH_3_); 3.89–3.84 (m, 4H, CH_2_); 3.83–3.78 (m, 4H, CH_2_) ppm. ^13^C‐NMR (100 MHz, CDCl_3_) δ: 164.7 (C); 159.9 (C); 152.2 (C); 147.2 (C); 132.7 (CH); 131.5 (C); 130.7 (C); 129.0 (CH); 127.8 (C); 126.6 (CH); 125.6 (CH); 125.2 (CH); 125.1 (CH); 124.7 (CH); 124.2 (CH); 115.0 (C); 107.9 (CH); 66.8 (CH_2_); 61.7 (CH_3_); 55.8 (CH_3_); 50.4 (CH_2_) ppm. ESI‐HRMS (*m/z*) calculated for [M+H]^+^ ion species C_24_H_24_N_3_O_3_= 402.1812, found 402.1812.

**Hydrochloride**: white solid; mp 219–221 (dec) °C.

#### 2‐(2,3‐Dimethoxynaphthalen‐1‐yl)‐4‐(4‐methylpiperazin‐1‐yl)quinazoline (6 d)

**Method B**: starting from the 4‐chloroquinazoline **22** (0.070 g, 0.20 mmol), 1‐methylpiperazine (0.022 mL, 0.20 mmol) and K_2_CO_3_ (0.028 g, 0.20 mmol) in 3.0 mL of dry DMF, compound **6 d** (0.050 g, yield: 60.3 %) was synthesized as a white solid.

**Free base**: Chromatographic eluent: CH_2_Cl_2_/CH_3_OH/NH_4_OH 98 : 2:0.2. ^1^H‐NMR (400 MHz, CDCl_3_) δ: 7.99 (d, *J*=9.2 Hz, 2H, CH arom.); 7.79‐7.68 (m, 2H, CH arom.); 7.49 (t, *J*=8.0 Hz, 1H, CH arom.); 7.43 (d, *J*=8.0 Hz, 1H, CH arom.); 7.34 (t, *J*=8.0 Hz, 1H, CH arom.); 7.25 (s, 1H, CH arom.); 7.21 (t, *J*=8.0 Hz, 1H, CH arom.); 4.01 (s, 3H, OCH_3_); 3.89 (s, 3H, OCH_3_); 3.86 (t, *J*=4.4 Hz, 4H, CH_2_); 2.62 (t, *J*=4.4 Hz, 4H, CH_2_); 2.36 (s, 3H, NCH_3_) ppm. ^13^C‐NMR (100 MHz, CDCl_3_) δ: 164.7 (C); 160.0 (C); 152.4 (C); 152.2 (C); 147.1 (C); 132.4 (CH); 131.5 (C); 131.0 (C); 129.0 (CH); 127.9 (C); 126.6 (CH); 125.3 (CH); 125.2 (CH); 125.1 (CH); 124.9 (CH); 124.1 (CH); 115.2 (C); 107.8 (CH); 61.7 (CH_3_); 55.8 (CH_3_); 55.0 (CH_2_); 49.7 (CH_2_); 46.1 (CH_3_) ppm. ESI‐HRMS (*m/z*) calculated for [M+H]^+^ ion species C_25_H_27_N_4_O_2_=415.2129, found 415.2132.

**Hydrochloride**: yellow solid; mp 181–183 (dec) °C.

#### 4‐(6,7‐Dimethoxy‐3,4‐dihydroisoquinolin‐2(1*H*)‐yl)‐2‐(2,3‐dimethoxynaphthalen‐1‐yl)quinazoline (6 e)

**Method B**: starting from the 4‐chloroquinazoline **22** (0.065 g, 0.19 mmol), 6,7‐dimethoxy‐1,2,3,4‐tetrahydroisoquinoline (0.036 g, 0.19 mmol) and K_2_CO_3_ (0.026 g, 0.19 mmol) in 3.0 mL of dry DMF, compound **6 e** (0.090 g, yield: 95.8 %) was synthesized as a white solid.

**Free base**: TLC: CH_2_Cl_2_/CH_3_OH/NH_4_OH 98 : 2:0.2. ^1^H‐NMR (400 MHz, CDCl_3_) δ: 8.07 (d, *J*=8.0 Hz, 1H, CH arom.); 8.04–7.96 (m, 1H, CH arom.); 7.79–7.71 (m, 2H, CH arom.); 7.52 (t, *J*=8.0 Hz, 1H, CH arom.); 7.42 (d, *J*=8.0 Hz, 1H, CH arom.); 7.35 (t, *J*=7.6 Hz, 1H, CH arom.); 7.26 (s, 1H, CH arom.); 7.20 (t, *J*=7.6 Hz, 1H, CH arom.); 6.67 (s, 1H, CH arom.); 6.60 (s, 1H, CH arom.); 4.90 (s, 2H, NCH_2_Ar); 4.08 (t, *J*=5.2 Hz, 2H, CH_2_); 4.02 (s, 3H, OCH_3_); 3.87 (s, 3H, OCH_3_); 3.86 (s, 3H, OCH_3_); 3.80 (s, 3H, OCH_3_); 3.07 (t, *J*=5.2 Hz, 2H, CH_2_) ppm. ^13^C‐NMR (100 MHz, CDCl_3_) δ: 163.9 (C); 152.2 (C); 147.9 (C); 147.8 (C); 147.1 (C); 132.5 (CH); 131.5 (C); 127.9 (C); 126.6 (CH); 126.5 (C); 125.7 (C); 125.2 (CH); 125.2 (CH); 124.9 (CH); 124.2 (CH); 114.9 (C); 111.6 (CH); 109.4 (CH); 107.8 (CH); 61.7 (CH_3_); 56.0 (CH_3_); 56.0 (CH_3_); 55.8 (CH_3_); 51.0 (CH_2_); 48.4 (CH_2_); 28.6 (CH_2_) ppm. ESI‐HRMS (*m/z*) calculated for [M+H]^+^ ion species C_31_H_30_N_3_O_4_=508.2231, found 508.2231.

**Hydrochloride**: white solid; mp 190–193 (dec) °C.

#### 2‐(Bis(4‐methoxyphenyl)methyl)‐*N*‐(4‐(2‐(6,7‐dimethoxy‐3,4‐dihydroisoquinolin‐2(1*H*)‐yl)ethyl)phenyl)quinazolin‐4‐amine (7 a)

**Method A**: starting from the 4‐chloroquinazoline **23** (0.080 g, 0.20 mmol) and 4‐(2‐(6,7‐dimethoxy‐3,4‐dihydroisoquinolin‐2(1*H*)‐yl)ethyl)aniline^[30]^ (0.064 g, 0.20 mmol) in 1.5 mL of abs. ethanol, compound **7 a** (0.040 g, yield: 29.3 %) was synthesized as a pale‐yellow solid.

**Free base**: Chromatographic eluent: CH_2_Cl_2_/CH_3_COCH_3_ 70 : 30. ^1^H‐NMR (400 MHz, CDCl_3_) δ: 7.85 (t, *J*=8.8 Hz, 2H, CH arom.); 7.71 (t, *J*=8.0 Hz, 1H, CH arom.); 7.63 (bs, 1H, NH); 7.52 (d, *J*=8.4 Hz, 2H, CH arom.); 7.43 (t, *J*=8.0 Hz, 1H, CH arom.); 7.30 (d, *J*=8.8 Hz, 4H, CH arom.); 7.13 (d, *J*=8.4 Hz, 2H, CH arom.); 6.81 (d, *J*=8.8 Hz, 4H, CH arom.); 6.60 (s, 1H, CH arom.); 6.54 (s, 1H, CH arom.); 5.63 (s, 1H, CH); 3.84 (s, 3H, OCH_3_); 3.83 (s, 3H, OCH_3_); 3.75 (s, 6H, OCH_3_); 3.69 (s, 2H, CH_2_); 2.93–2.77 (m, 8H, CH_2_) ppm. ^13^C‐NMR (100 MHz, CDCl_3_) δ: 167.6 (C); 158.1 (C); 157.3 (C); 150.6 (C); 147.6 (C); 147.3 (C); 136.9 (C); 135.5 (C); 135.1 (C); 132.7 (CH); 130.4 (CH); 128.9 (CH); 128.6 (CH); 126.1 (C); 126.0 (CH); 121.2 (CH); 120.5 (CH); 113.5 (CH); 111.4 (CH); 109.5 (CH); 60.1 (CH_2_); 59.2 (CH); 55.9 (CH_3_); 55.9 (CH_3_); 55.6 (CH_2_); 55.2 (CH_3_); 51.0 (CH_2_); 33.3 (CH_2_); 28.5 (CH_2_) ppm. ESI‐HRMS (*m/z*) calculated for [M+H]^+^ ion species C_42_H_43_N_4_O_4_= 667.3279, found 667.3282.

**Hydrochloride**: yellow solid; mp 258–260 (dec) °C.

#### 2‐(Bis(4‐methoxyphenyl)methyl)‐*N*‐phenethylquinazolin‐4‐amine (7 b)

**Method B**: starting from the 4‐chloroquinazoline **23** (0.060 g, 0.15 mmol), 2‐phenylethanamine (0.019 mL, 0.15 mmol) and K_2_CO_3_ (0.021 g, 0.15 mmol) in 3.0 mL of dry DMF, compound **7 b** (0.070 g, yield: 95.7 %) was synthesized as a pale‐yellow solid.

**Free base**: TLC: CH_2_Cl_2_/CH_3_OH/NH_4_OH 97 : 3:0.3. ^1^H‐NMR (400 MHz, CDCl_3_) δ: 9.80 (bs, 1H, NH); 7.75 (d, *J*=8.0 Hz, 1H, CH arom.); 7.63 (d, *J*=8.0 Hz, 1H, CH arom.); 7.48‐7.37 (m, 5H, CH arom.); 7.34–7.20 (m, 3H, CH arom.); 7.15–7.01 (m, 3H, CH arom.); 6.84 (d, *J*=8.4 Hz, 4H, CH arom.); 5.64 (s, 1H, CH); 3.86‐3.76 (m, 2H, CH_2_); 3.75 (s, 6H, OCH_3_); 2.90 (t, *J*=7.2 Hz, 2H, CH_2_) ppm. ^13^C‐NMR (100 MHz, CDCl_3_) δ: 167.3 (C); 159.8 (C); 158.2 (C); 148.5 (C); 139.2 (C); 134.9 (C); 132.7 (CH); 130.4 (CH); 128.8 (CH); 128.6 (CH); 126.8 (CH); 126.5 (CH); 125.7 (CH); 121.2 (CH); 113.5 (CH); 113.3 (C); 58.5 (CH); 55.2 (CH_3_); 42.8 (CH_2_); 35.4 (CH_2_) ppm. ESI‐HRMS (*m/z*) calculated for [M+H]^+^ ion species C_31_H_30_N_3_O_2_= 476.2333, found 476.2333.

**Hydrochloride**: white solid; mp 155–158 (dec) °C.

#### 4‐(2‐(Bis(4‐ethoxyphenyl)methyl)quinazolin‐4‐yl)morpholine (7 c)

**Method B**: starting from the 4‐chloroquinazoline **23** (0.050 g, 0.13 mmol), morpholine (0.011 mL, 0.13 mmol) and K_2_CO_3_ (0.018 g, 0.13 mmol) in 3.0 mL of dry DMF, compound **7 c** (0.040 g, yield: 70.9 %) was synthesized as a white solid.

**Free base**: TLC: CH_2_Cl_2_/CH_3_OH/NH_4_OH 97 : 3:0.3. ^1^H‐NMR (400 MHz, CDCl_3_) δ: 7.93 (d, *J*=8.4 Hz, 1H, CH arom.); 7.81 (d, *J*=8.4 Hz, 1H, CH arom.); 7.69 (t, *J*=8.4 Hz, 1H, CH arom.); 7.41–7.34 (m, 5H, CH arom.); 6.82 (d, *J*=8.4 Hz, 4H, CH arom.); 5.61 (s, 1H, CH); 3.84‐3.78 (m, 4H, CH_2_); 3.77–3.72 (m, 10H, OCH_3_ and CH_2_) ppm. ^13^C‐NMR (100 MHz, CDCl_3_) δ: 166.0 (C); 164.6 (C); 158.1 (C); 152.0 (C); 135.1 (C); 132.5 (CH); 130.2 (CH); 128.4 (CH); 125.1 (CH); 124.5 (CH); 114.6 (C); 113.5 (CH); 66.7 (CH_2_); 58.8 (CH); 55.2 (CH_3_); 50.2 (CH_2_) ppm. ESI‐HRMS (*m/z*) calculated for [M+H]^+^ ion species C_27_H_28_N_3_O_3_=442.2125, found 442.2127.

**Hydrochloride**: white solid; mp 236–238 (dec) °C.

#### 2‐(Bis(4‐methoxyphenyl)methyl)‐4‐(4‐methylpiperazin‐1‐yl)quinazoline (7 d).

**Method B**: starting from the 4‐chloroquinazoline **23** (0.060 g, 0.15 mmol), 1‐methylpiperazine (0.017 mL, 0.15 mmol) and K_2_CO_3_ (0.021 g, 0.15 mmol) in 3.0 mL of dry DMF, compound **7 d** (0.030 g, yield: 42.9 %) was synthesized as a white solid.

**Free base**: TLC: CH_2_Cl_2_/CH_3_OH/NH_4_OH 97 : 3:0.3. ^1^H‐NMR (400 MHz, CDCl_3_) δ: 7.89 (d, *J*=8.4 Hz, 1H, CH arom.); 7.81 (d, *J*=8.4 Hz, 1H, CH arom.); 7.68 (t, *J*=8.4 Hz, 1H, CH arom.); 7.40‐7.33 (m, 5H, CH arom.); 6.81 (d, *J*=8.4 Hz, 4H, CH arom.); 5.58 (s, 1H, CH); 3.88‐3.78 (m, 4H, CH_2_); 3.75 (s, 6H, OCH_3_); 2.69–2.51 (m, 4H, CH_2_); 2.36 (s, 3H, CH_3_) ppm. ^13^C‐NMR (100 MHz, CDCl_3_) δ: 166.1 (C); 164.6 (C); 158.1 (C); 152.3 (C); 135.2 (C); 132.5 (CH); 130.2 (CH); 128.5 (CH); 125.1 (CH); 124.6 (CH); 114.8 (C); 113.4 (CH); 58.9 (CH); 55.2 (CH_3_); 54.3 (CH_2_); 49.0 (CH_2_); 45.6 (CH_3_) ppm. ESI‐HRMS (*m/z*) calculated for [M+H]^+^ ion species C_28_H_31_N_4_O_2_= 455.2442, found 455.2442.

**Hydrochloride**: white solid; mp 233–235 (dec) °C.

#### 2‐(Bis(4‐methoxyphenyl)methyl)‐4‐(6,7‐dimethoxy‐3,4‐dihydroisoquinolin‐2(1*H*)‐yl)quinazoline (7 e)

**Method B**: starting from the 4‐chloroquinazoline **23** (0.061 g, 0.16 mmol), 6,7‐dimethoxy‐1,2,3,4‐tetrahydroisoquinoline (0.030 g, 0.16 mmol) and K_2_CO_3_ (0.022 g, 0.16 mmol) in 3.0 mL of dry DMF, compound **7 e** (0.070 g, yield: 82.5 %) was synthesized as a pale‐yellow solid.

**Free base**: TLC: CH_2_Cl_2_/CH_3_OH/NH_4_OH 97 : 3:0.3. ^1^H‐NMR (400 MHz, CDCl_3_) δ: 7.93 (d, *J*=8.4 Hz, 1H, CH arom.); 7.88 (d, *J*=8.4 Hz, 1H, CH arom.); 7.67 (t, *J*=8.4 Hz, 1H, CH arom.); 7.41‐7.35 (m, 5H, CH arom.); 6.82 (d, *J*=8.4 Hz, 4H, CH arom.); 6.66 (s, 1H, CH arom.); 6.63 (s, 1H, CH arom.); 5.59 (s, 1H, CH); 4.86 (s, 2H, CH_2_); 4.02‐3.98 (m, 2H, CH_2_); 3.88 (s, 3H, OCH_3_); 3.86 (s, 3H, OCH_3_); 3.76 (s, 6H, OCH_3_); 2.96‐2.90 (m, 2H, CH_2_) ppm. ^13^C‐NMR (100 MHz, CDCl_3_) δ: 166.0 (C); 164.0 (C); 158.1 (C); 152.5 (C); 147.8 (C); 147.7 (C); 135.4 (C); 132.2 (CH); 130.3 (CH); 128.4 (CH); 126.6 (C); 125.7 (C); 124.8 (CH); 124.6 (CH); 114.8 (C); 113.4 (CH); 111.6 (CH); 109.3 (CH); 59.1 (CH); 56.1 (CH_3_); 56.0 (CH_3_); 55.2 (CH_3_); 51.2 (CH_2_); 47.8 (CH_2_); 28.1 (CH_2_) ppm. ESI‐HRMS (*m/z*) calculated for [M+H]^+^ ion species C_34_H_34_N_3_O_4_= 548.2544, found 548.2548.

**Hydrochloride**: yellow solid; mp 186–189 (dec) °C.

### Biology

#### Materials

Cell culture reagents were purchased from Celbio s.r.l. (Milano, Italy). CulturePlate 96/wells plates were purchased from PerkinElmer Life Science (Waltham, MA) and Falcon (BD Biosciences, Bedford, MA). Calcein‐AM, bisBenzimide H 33342 trihydrochloride were obtained from Sigma‐Aldrich (Milan, Italy).

Cell cultures. MDCK‐MDR1, MDCK‐MRP1 and MDCK‐BCRP cells are a gift of Prof. P. Borst, NKI‐AVL Institute, Amsterdam, The Netherlands. Caco‐2 cells were a gift of Dr. Aldo Cavallini and Dr. Caterina Messa from the Laboratory of Biochemistry, National Institute for Digestive Diseases, “S. de Bellis”, Bari (Italy). HT29 and HT29/DX cells from ATCC (Manassas, VA).

MDCK and Caco‐2 cells were grown in DMEM high glucose supplemented with 10 % fetal bovine serum, 2 mM glutamine, 100 U/mL penicillin, 100 μg/mL streptomycin, in a humidified incubator at 37 °C with a 5 % CO2 atmosphere.

HT29 cells were grown in RPMI‐1640 supplemented with 10 % fetal bovine serum (FBS), 2 mM glutamine, 100 U/mL penicillin, 100 mg/mL streptomycin; HT29/DX cells were grown in the above mentioned medium containing 50 nM doxorubicin to maintain the chemoresistant phenotype.^[32]^

##### Calcein‐AM experiment

The experiments were carried out as described by Contino et al. with minor modifications.^[49]^ Each cell line (30,000 cells per well) was seeded into black CulturePlate 96/wells plate with 100 μL medium and allowed to become confluent overnight. 100 μL of test compounds, solubilized in culture medium, were added to monolayers, with final concentrations ranging from 0.1 to 100 μM. Thus, after a 30 min incubation time at 37 °C, calcein‐AM was added in 100 μL of Phosphate Buffered Saline (PBS) to the 96/wells plate to yield a final concentration of 2.5 μM; the plate was then incubated for 30 min. Each well was washed 3 times with ice cold PBS and saline buffer was added to each well and the plate read with Victor3 (PerkinElmer) at excitation and emission wavelengths of 485 nm and 535 nm, respectively. In these experimental conditions, calcein cell accumulation in the absence and in the presence of tested compounds was evaluated and fluorescence basal level was estimated with untreated cells. In treated wells, the increase of fluorescence with respect to basal level was measured. EC50 values were determined by fitting the fluorescence increase percentage versus log[dose].

##### Hoechst 33342 experiment

These experiments were carried out as described by Contino et al. with minor modifications.^[49]^ Each cell line (30,000 cells per well) was seeded into black CulturePlate 96/wells plate with 100 μL medium and allowed to become confluent overnight. 100 μL of test compounds, solubilized in culture medium, were added to monolayers, with final concentrations ranging from 0.1 to 100 μM. After 30 min incubation time at 37 °C, Hoechst 33342 was added in 100 μL of Phosphate Buffered Saline (PBS) to yield a final concentration of 8 μM to the 96/wells plate and the plate incubated for 30 min. The supernatants were drained and the cells were fixed for 20 min under light protection using 100 μL per well of a 4 % PFA solution. Each well was washed 3 times with ice cold PBS and saline buffer added to each well and the plate read with Victor3 (PerkinElmer) at excitation and emission wavelengths of 340/35 nm and 485/20 nm, respectively. In these experimental conditions, Hoechst 33342 accumulation in the absence and in the presence of tested compounds was evaluated and fluorescence basal level was estimated with untreated cells. In treated wells the increase of fluorescence with respect to basal level was measured. EC_50_ values were determined by fitting the fluorescence increase percentage versus log[dose].

##### Preparation of Caco‐2 monolayer

The experiments were carried out as described by Contino et al. with minor modifications.^[49]^ Caco‐2 cells were seeded onto a Millicell assay system (Millipore), in which a cell monolayer was set in between a filter cell and a receiver plate at a density of 20000 cells/well. The culture medium was replaced every 48 h, and the cells were kept for 21 days in culture. The trans epithelial electrical resistance (TEER) of the monolayers was measured daily, before and after the experiment, by using an epithelial voltohmmeter (Millicell‐ERS). Generally, TEER values greater than 1000 Ω for a 21 day culture are considered optimal.

##### Drug‐transport experiment

After 21 days of Caco‐2 cell growth, the medium was removed from the filter wells and from the receiver plate, and they were filled with fresh Hank's balanced salt solution (HBSS) buffer (Invitrogen). This procedure was repeated twice, and the plates were incubated at 37 °C for 30 min. After the incubation time, the HBSS buffer was removed, and drug solutions and reference compounds were added to the filter well at a concentration of 100 μM, whereas fresh HBSS was added to the receiver plate. The plates were incubated at 37 °C for 120 min. Afterward, samples were removed from the apical (filter well) and basolateral (receiver plate) side of the monolayer to measure the permeability. The apparent permeability (*P_app_*), in units of nms‐1, was calculated by using the same equation reported above.

##### ATPlite assay

The experiments were carried out as described by Contino et al. with minor modifications.^[49]^ The MDCK‐MDR1 cells seeded into 96‐well microplate in 100 μL of complete medium at a density 2×10^4^ cells/well were incubated overnight (O/N) in a humidified atmosphere 5 % CO_2_ at 37 °C. The medium was removed and 100 μL of complete medium either alone or containing different concentrations of test compounds were added. The plate was incubated for 2 h in a humidified 5 % CO_2_ atmosphere at 37 °C. 50 μL of mammalian cell lysis solution was added to all wells and the plate shaken for five minutes in an orbital shaker. 50 μL of substrate solution was added to all wells and the plate shaken for five minutes in an orbital shaker. The plate was dark adapted for ten minutes and the luminescence was measured.

##### Co‐administration assay in MDCK‐MDR1 and HT29/DX cells

The co‐administration assay with Doxorubicin was performed in MDCK‐MDR1, HT29 and HT29/DX cells at 48 h as reported with minor modifications.^[49]^ On day 1, 10000 cells/well were seeded into 96‐well plates in a volume of 100 μL of fresh medium. On day 2, the tested drug was added alone to the cells at different concentrations (10 nM, 100 nM, 500 nM, 1 μM, 10 μM). On day 3, the medium was removed and the drug at the same concentrations was added alone and in co‐administration with 10 μM Doxorubicin to the cells. After the established incubation time with the tested drug, MTT (0.5 mg/mL) was added to each well, and after 3–4 h incubation at 37 °C, the supernatant was removed. The formazan crystals were solubilized using 100 μL of DMSO/EtOH (1 : 1), and the absorbance values at 570 and 630 nm were determined on the microplate reader Victor 3 from PerkinElmer Life Sciences.

##### Intracellular doxorubicin accumulation and kinetic parameters

Doxorubicin content was measured after incubating 10000 HT29 and HT29/DX cells, seeded into 96‐well plates in a volume of 100 μL of fresh medium, for 24 h with 5 μM doxorubicin, in the absence or presence of increasing concentration of compound **1 e**. Cells were collected and the intracellular drug content was measured fluorimetrically as detailed previously,^[47]^ using a Synregy HTX 96‐well plate reader (Bio‐Tek Instruments, Winooski, VT). The results were expressed as nmol doxorubicin/mg cell proteins, according to a titration curve previously set. For the calculation of the kinetic parameters of doxorubicin efflux (Km and maximal velocity, Vmax), cells were incubated for 20 min with increasing (0–100 μmol/L) concentrations of doxorubicin, alone or with compound **1 e** at 10 μM, then washed and analysed for the intracellular concentration of doxorubicin. A second series of dishes, after the incubation with doxorubicin formulations under the same experimental conditions, were left for further 10 min at 37 °C, then washed and tested for the intracellular drug content. The difference of doxorubicin concentration between the two series, expressed as nmol doxorubicin extruded/min/mg cell protein was plotted versus the initial drugs’ concentration. Values were fitted to Michaelis‐Menten equation to calculate Vmax and Km, using the Enzfitter software (Biosoft Corporation, Cambridge, United Kingdom).^[47]^

##### Statistical analysis

All data in the text and figures are provided as means ±SEM. The results were analyzed by a Student's t‐test and ANOVA test, using Graph‐Pad Prism (Graph‐Pad software, San Diego, CA, USA). p <0.05 was considered significant.

##### Molecular modeling studies

Initial structure of murine P‐gp (4XWK^[44]^ was retrieved from Protein Data Bank (www.rcsb.org^[50]^)). Inner missing regions were modeled using Modeller^[51]^ as implemented in UCSF Chimera 1.11.2.^[52]^ The structure was then minimized with Amber force field ff14SB.^[53]^ Molecular docking was carried out with Gold software v. 2020.2.0^[45]^ using default settings. PyMOL was used for analysis and picture rendering (The PyMOL Molecular Graphics System, Version 1.8 Schrödinger, LLC.).

## Abbreviations

P‐gp: P‐glycoprotein
MRP1: multidrug‐resistance‐associated protein‐1
BCRP: breast cancer resistance protein
Doxo: Doxorubicin
HATU: 1‐[bis(dimethylamino)methylene]‐1*H*‐1,2,3‐triazolo[4,5‐*b*]pyridinium 3‐oxid hexafluorophosphate
DIPEA: *N*,*N*‐diisopropylethylamine;
PDB: Protein Data Bank;
*P_app_*
: apparent permeability
BA: basolateral to apical
AB: apical to basolateral
Calcein‐AM: calcein acetoxy methyl ester

## Supporting Information

^1^H‐NMR (400 MHz) and ^13^C‐APT‐NMR (100 MHz) spectra of compounds **1**–**7**.

## Conflict of interest

The authors declare no conflict of interest.

1

## Supporting information

As a service to our authors and readers, this journal provides supporting information supplied by the authors. Such materials are peer reviewed and may be re‐organized for online delivery, but are not copy‐edited or typeset. Technical support issues arising from supporting information (other than missing files) should be addressed to the authors.

Supporting Information

## Data Availability

The data that support the findings of this study are available in the supplementary material of this article.
