# Supplementary material for: Overcoming Multidrug Resistance (MDR): Design, Biological Evaluation and Molecular Modelling Studies of 2,4‐Substituted Quinazoline Derivatives
Source: ChemMedChem. 2022 May 12;17(12):e202200027. doi: 10.1002/cmdc.202200027 (PMC9325490; doi:10.1002/cmdc.202200027)

ChemMedChem

Supporting Information

Overcoming Multidrug Resistance (MDR): Design, Biological Evaluation and Molecular Modelling Studies of 2,4-Substituted Quinazoline Derivatives

Laura Braconi, Elisabetta Teodori,* Marialessandra Contino, Chiara Riganti, Gianluca Bartolucci, Dina Manetti, Maria Novella Romanelli, Maria Grazia Perrone, Nicola Antonio Colabufo, Stefano Guglielmo, and Silvia Dei

Table of Contents

^{1}H-NMR (400 MHz), ^{13}C-APT- NMR (100 MHz) spectra of compounds **1-7.**

^{1}H-NMR and ^{13}C-APT-NMR spectra of compound **1a**

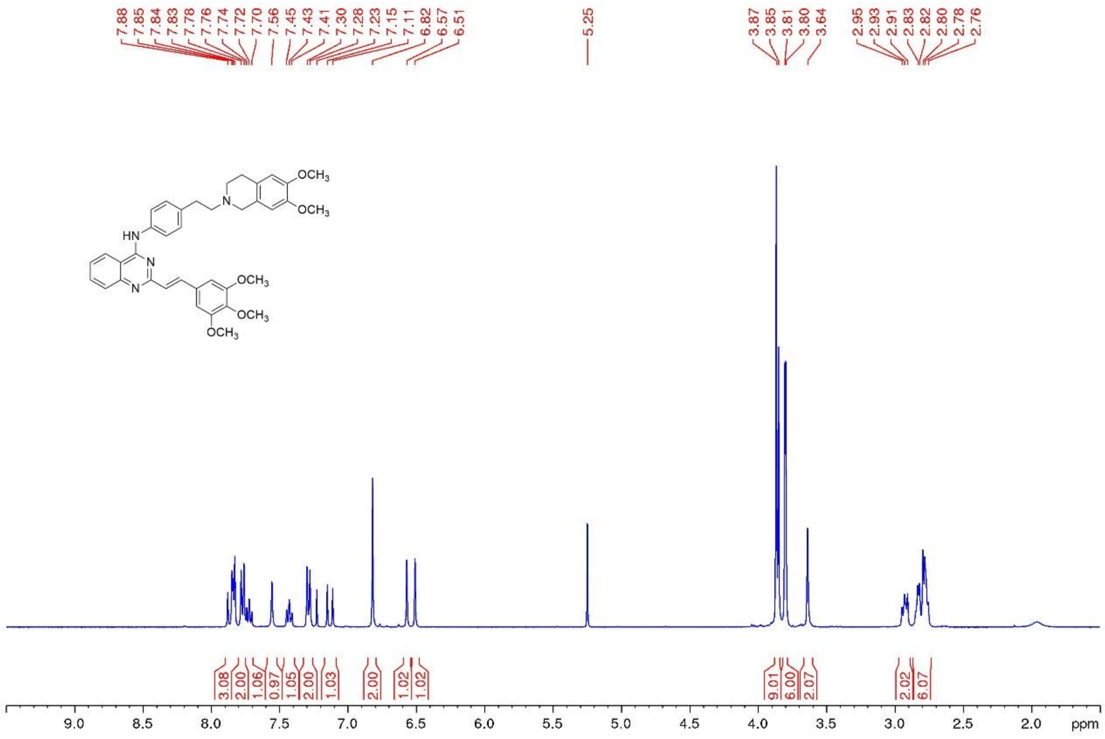

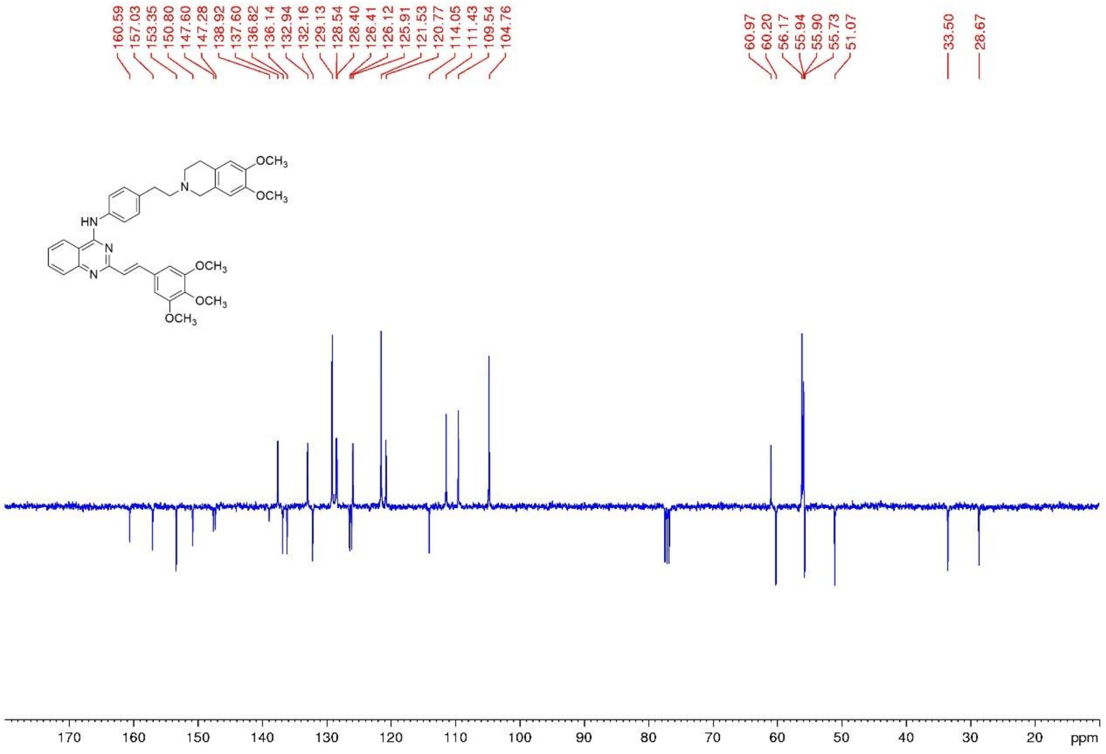

^{13}C-APT-NMR spectrum of compound **1b**

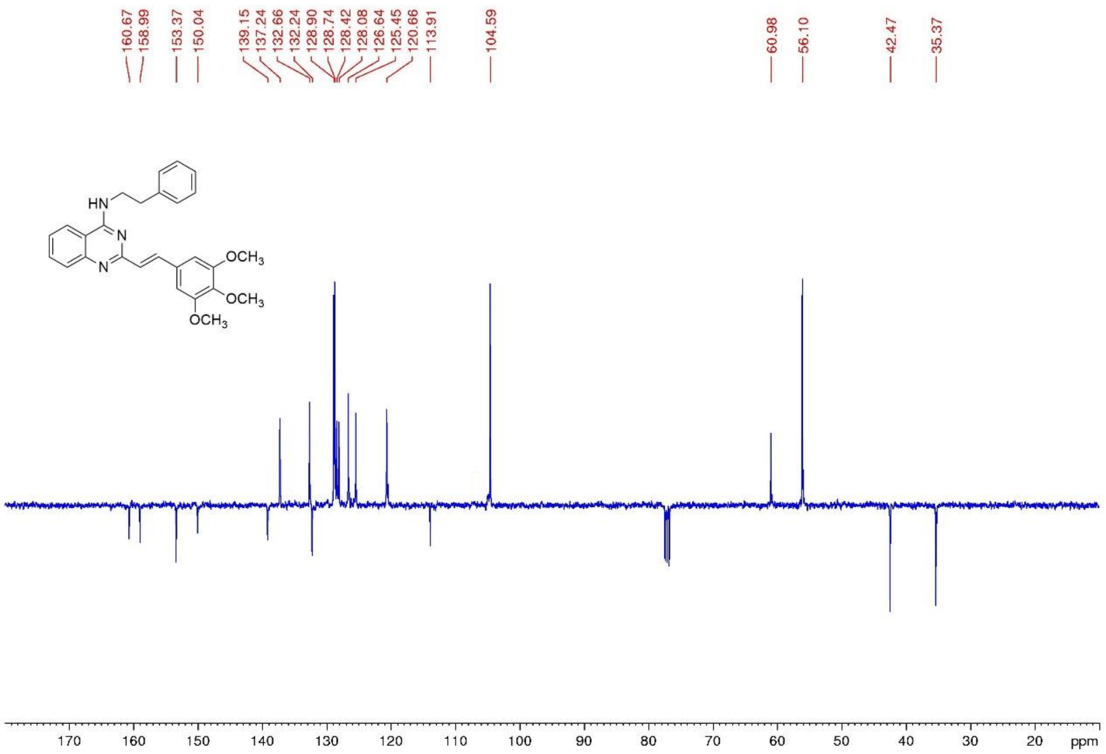

^{1}H-NMR and ^{13}C-APT-NMR spectra of compound **1c**

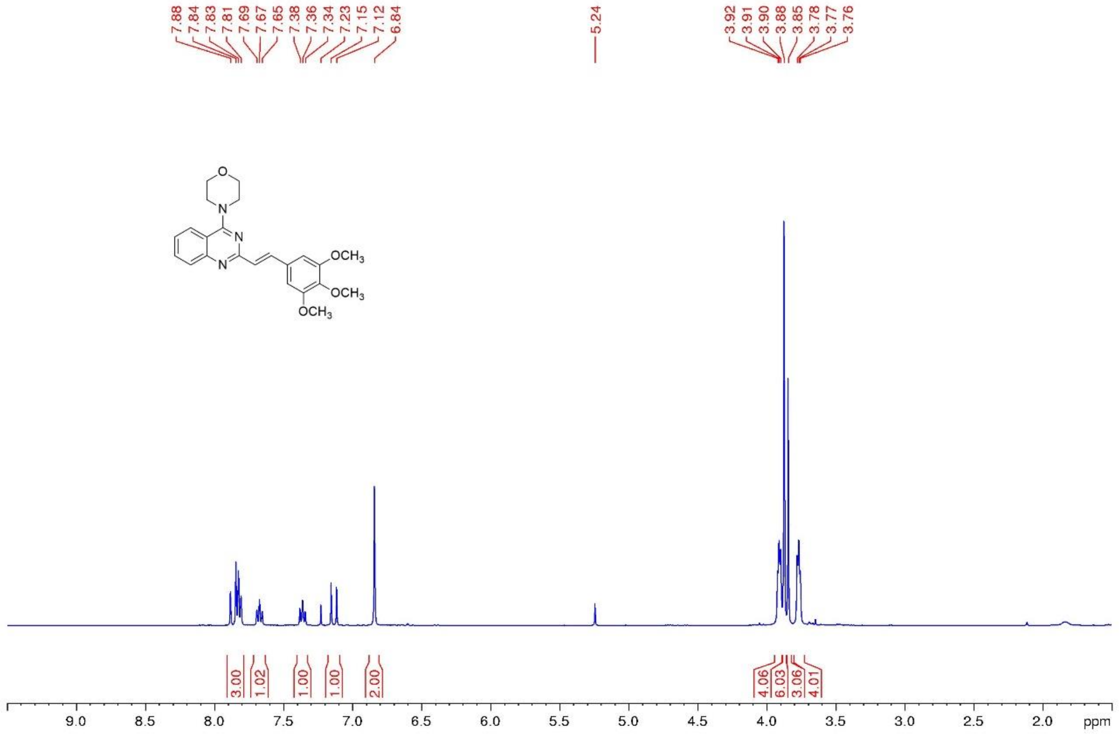

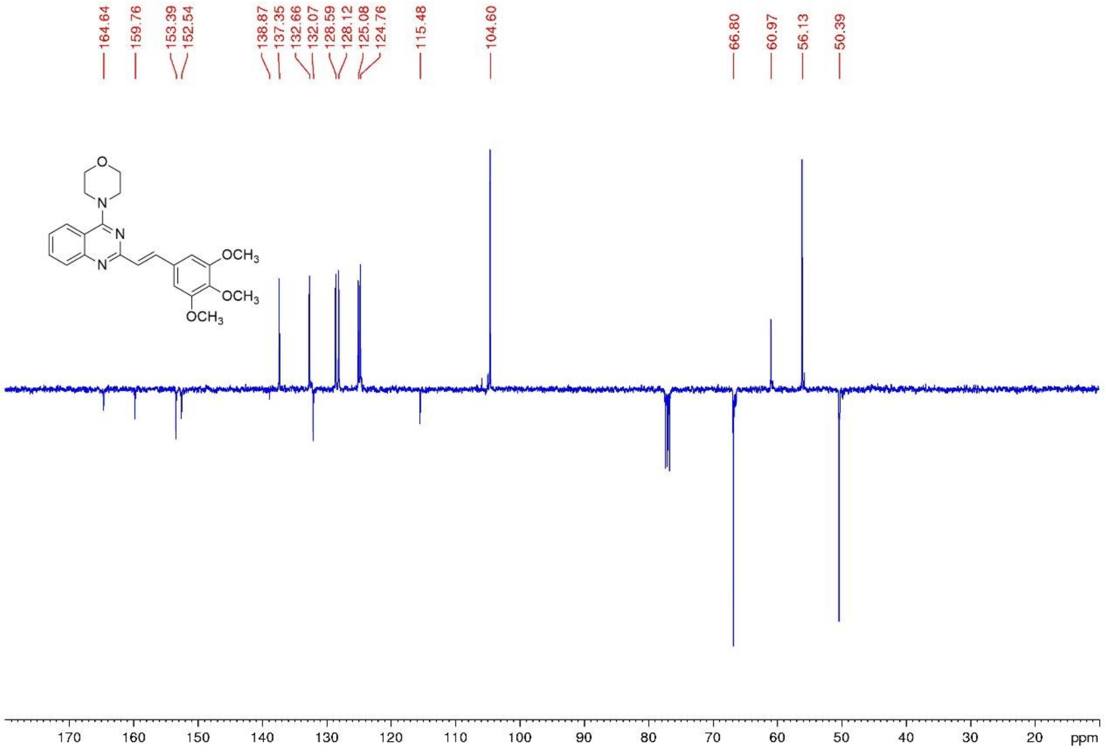

^{1}H-NMR and ^{13}C-APT-NMR spectra of compound **1d**

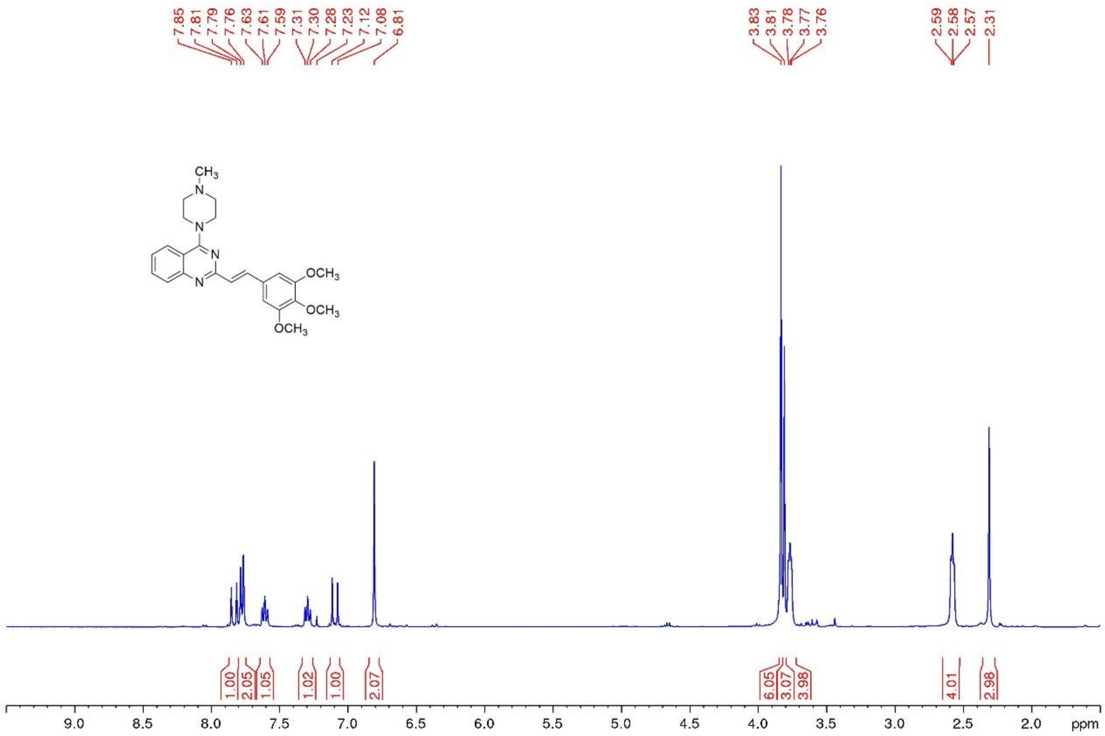

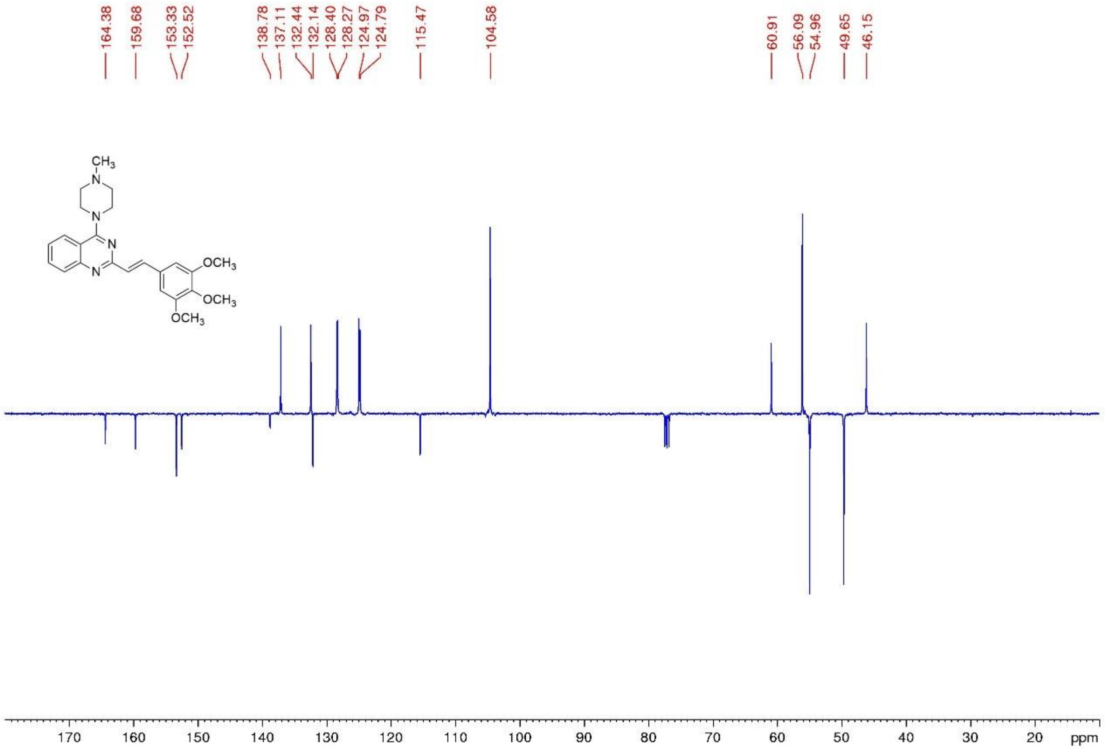

^{1}H-NMR and ^{13}C-APT-NMR spectra of compound **1e**

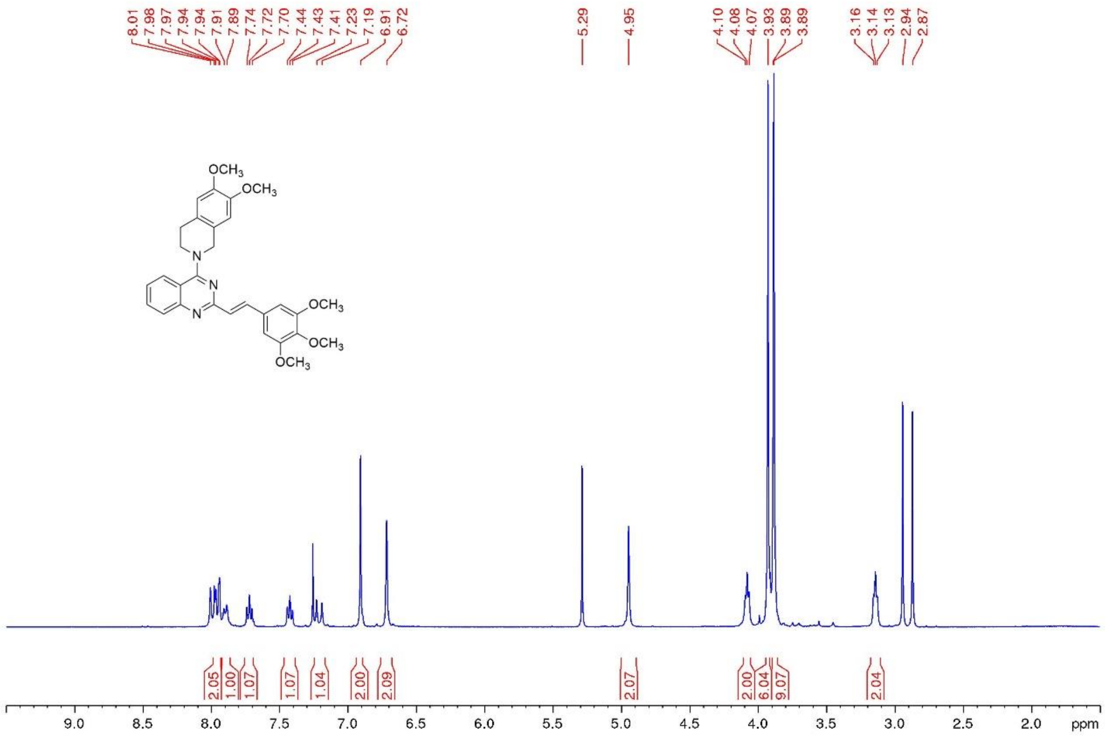

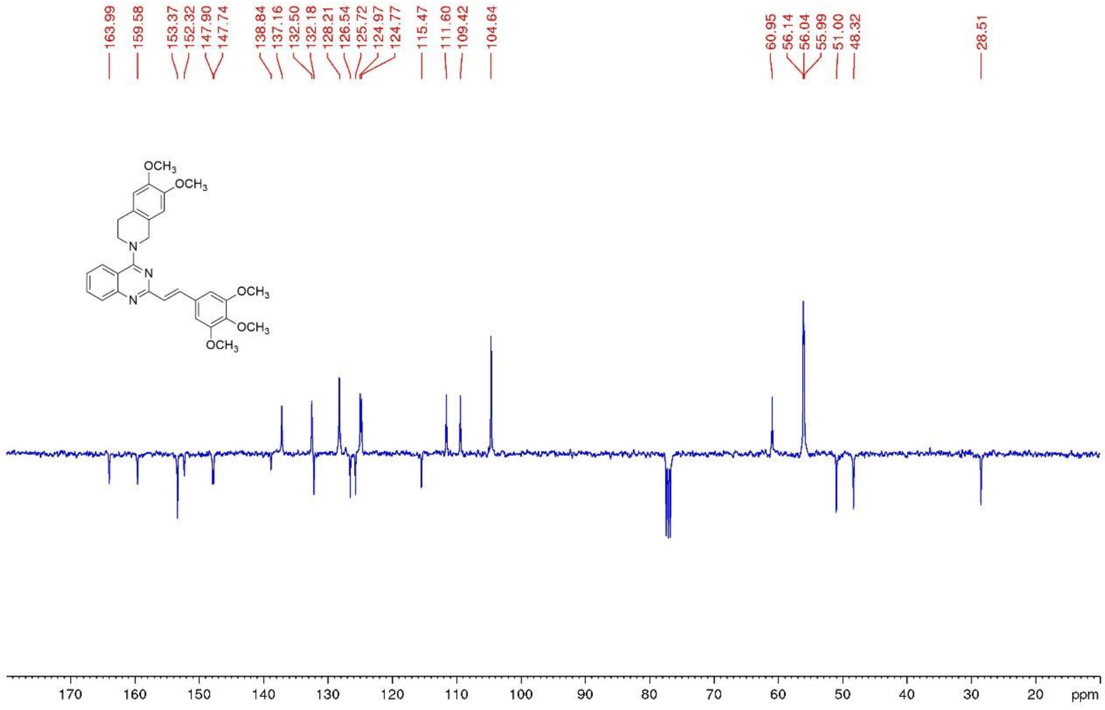

^{1}H-NMR and ^{13}C-APT-NMR spectra of compound **2a**

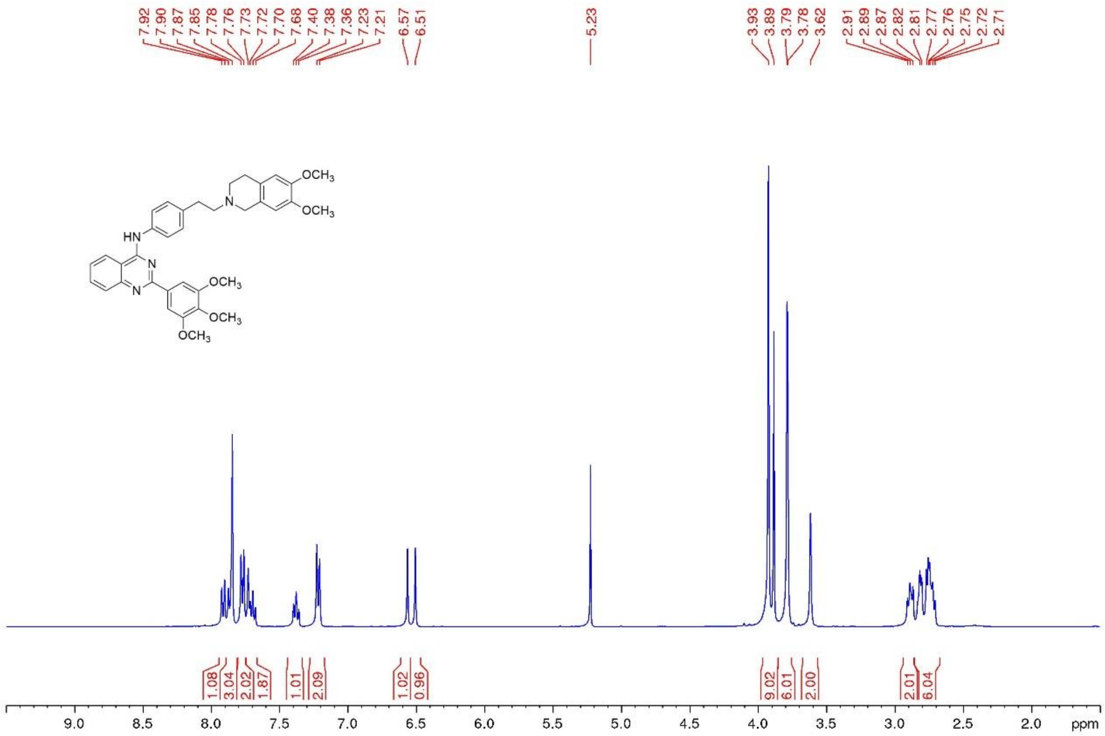

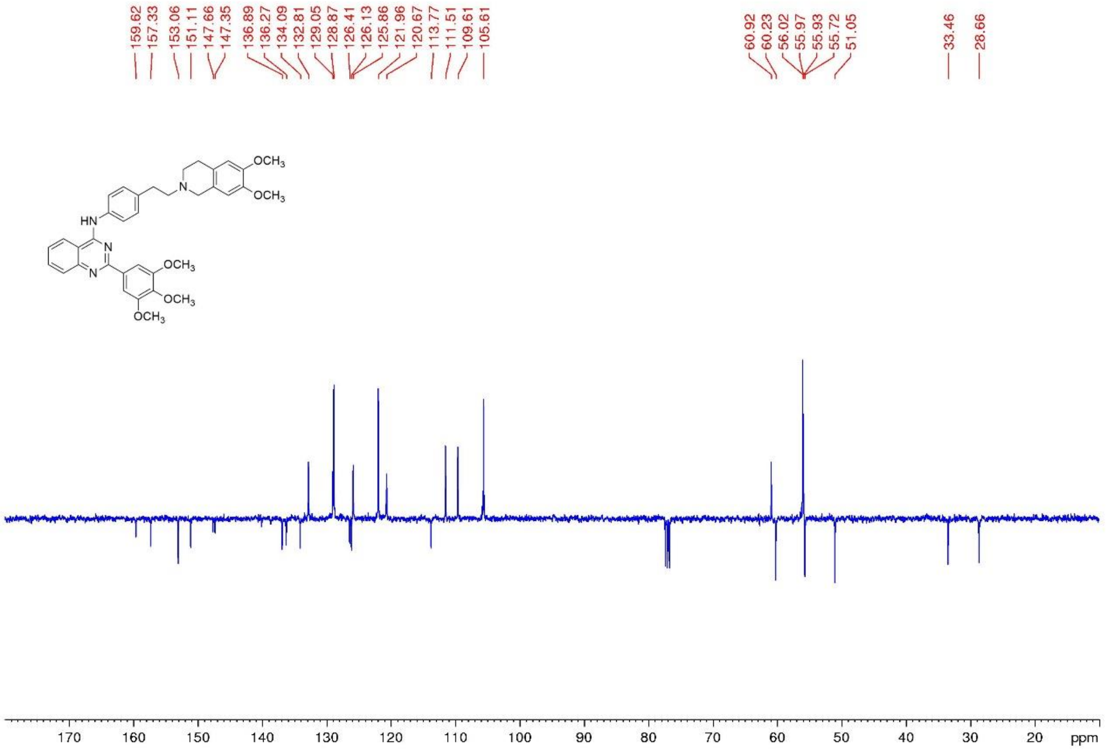

^{1}H-NMR and ^{13}C-APT-NMR spectra of compound **2b**

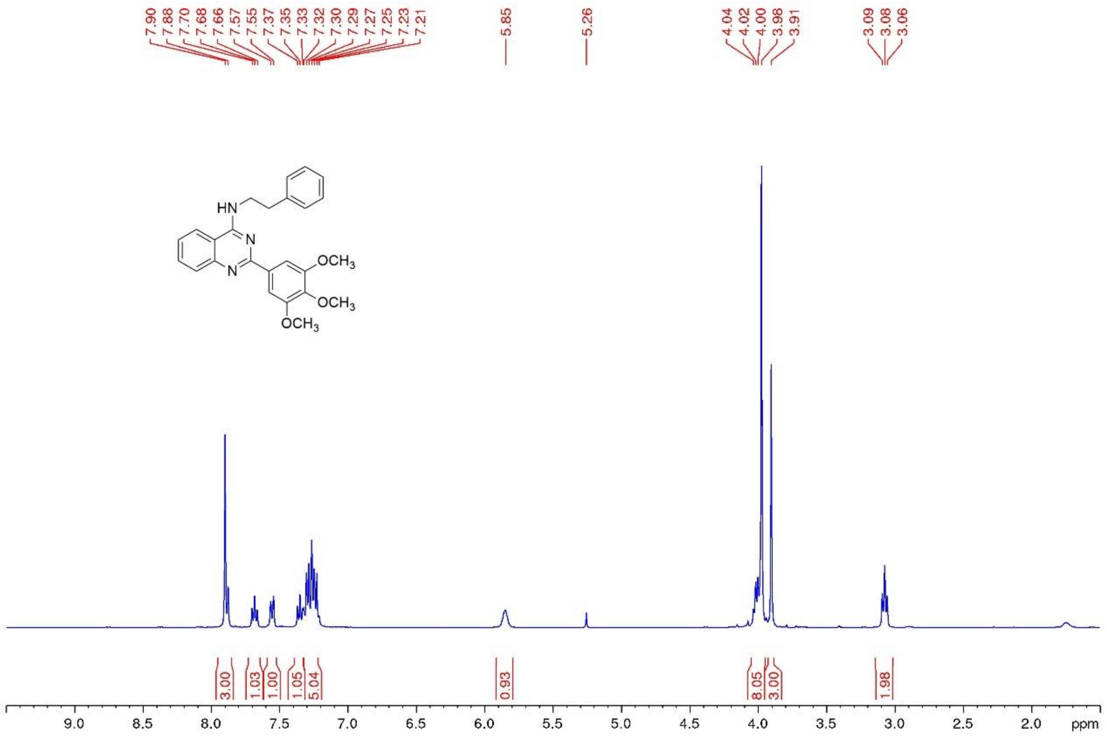

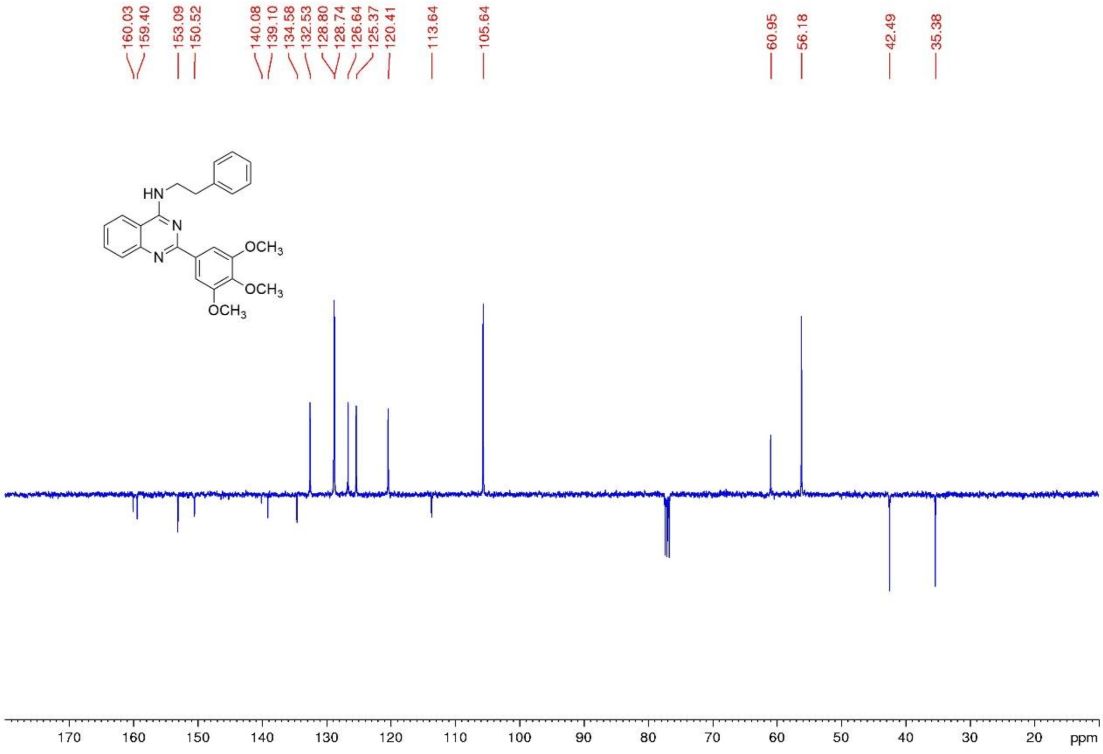

^{1}H-NMR and ^{13}C-APT-NMR spectra of compound **2c**

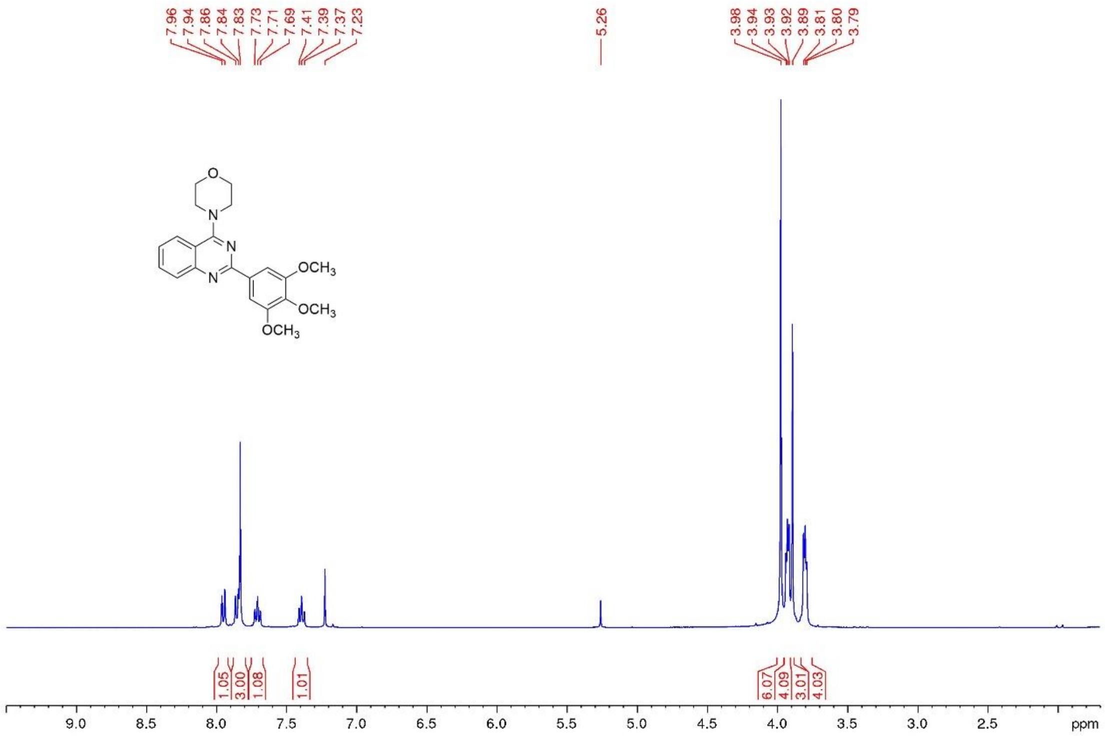

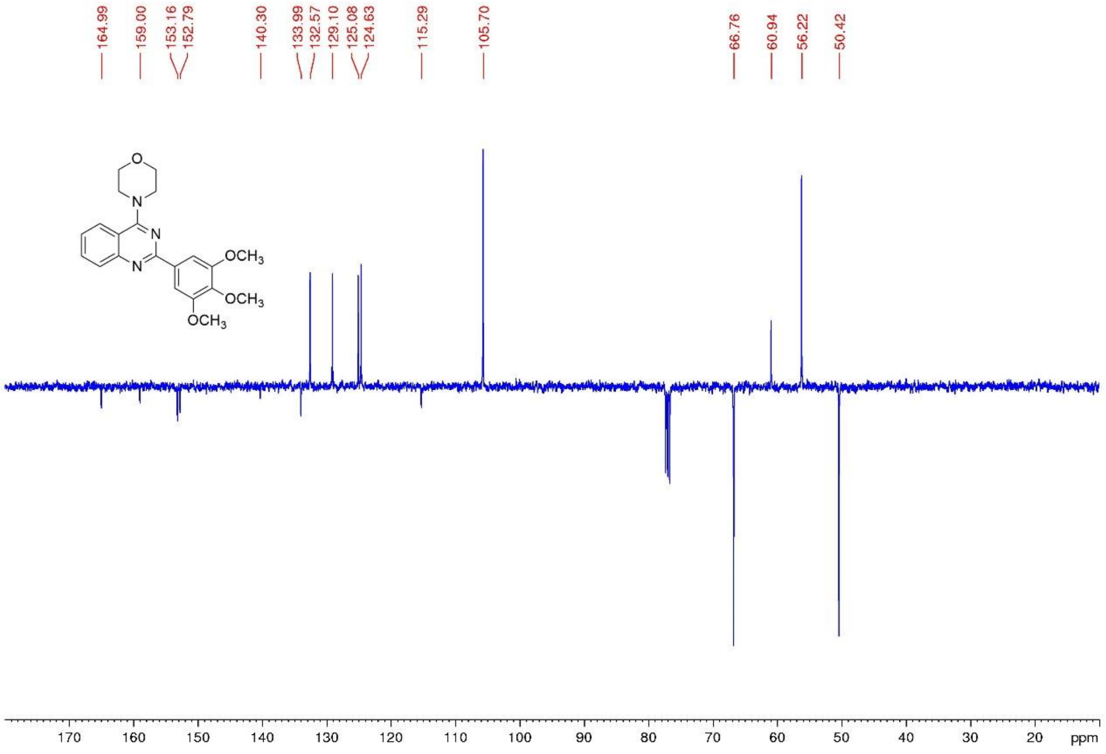

^{1}H-NMR and ^{13}C-APT-NMR spectra of compound **2d**

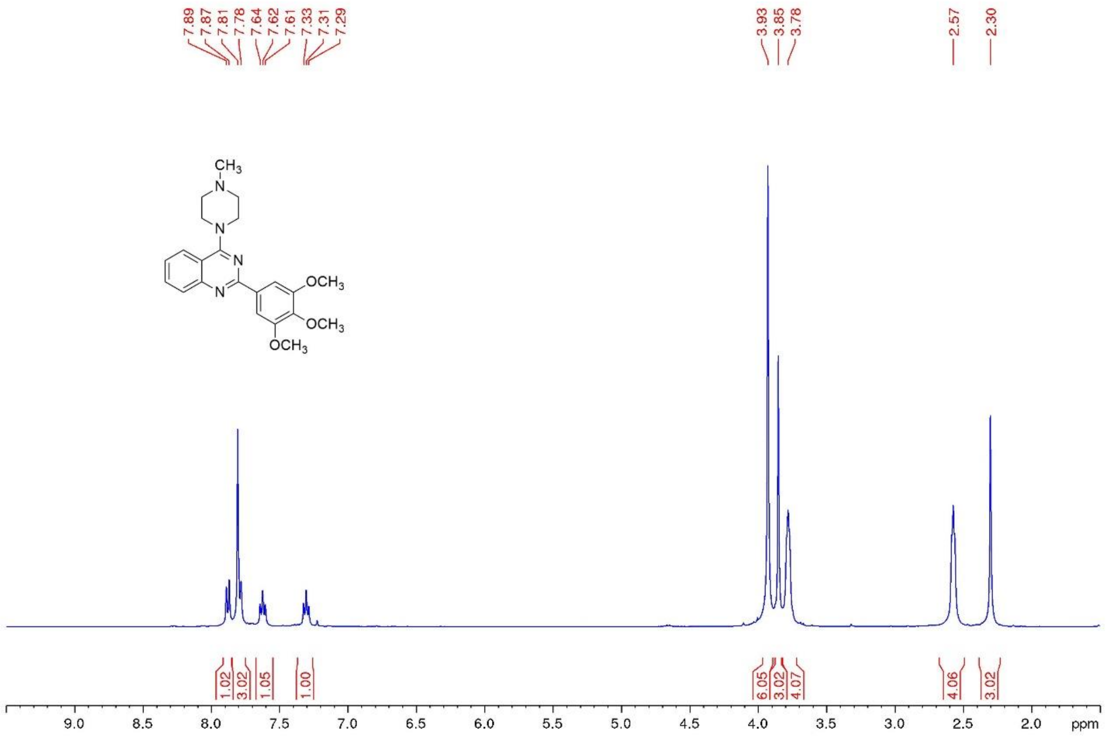

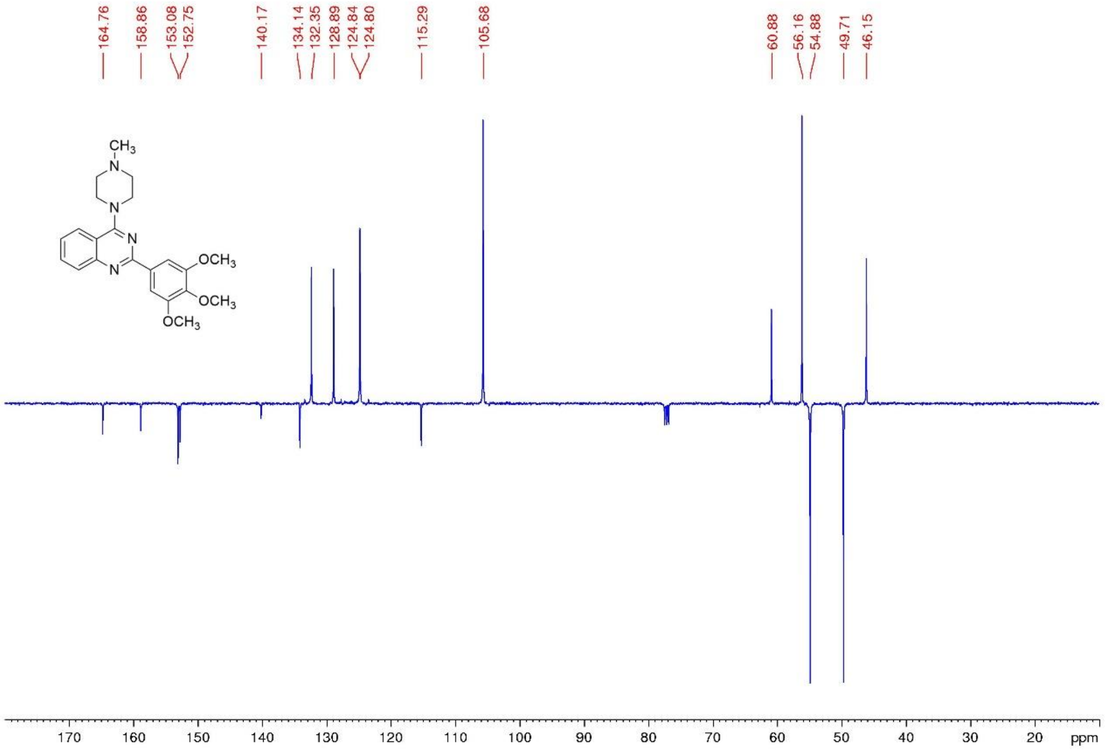

^{1}H-NMR and ^{13}C-APT-NMR spectra of compound **2e**

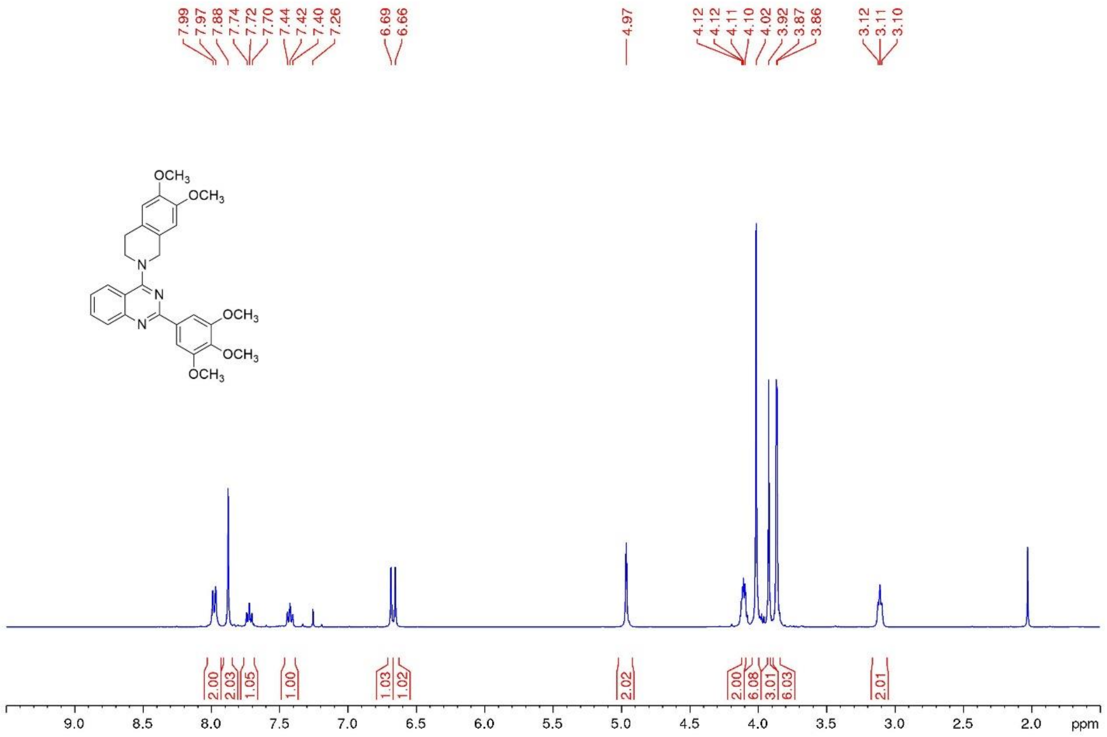

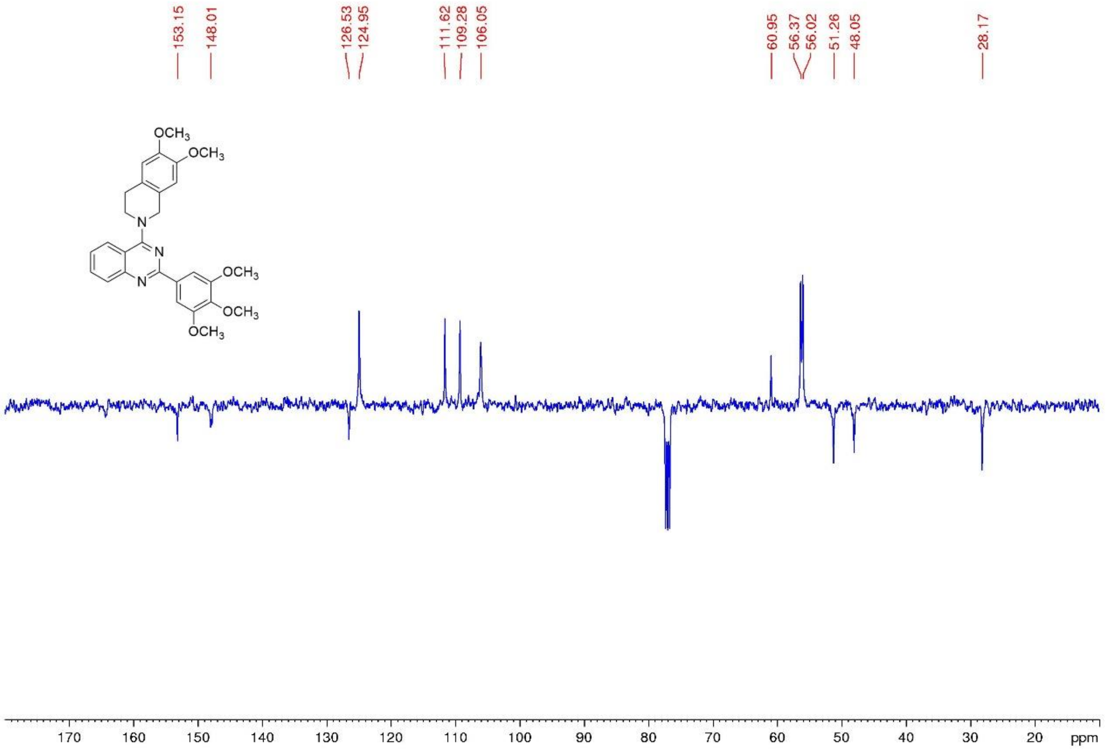

^{1}H-NMR and ^{13}C-APT-NMR spectra of compound **3a**

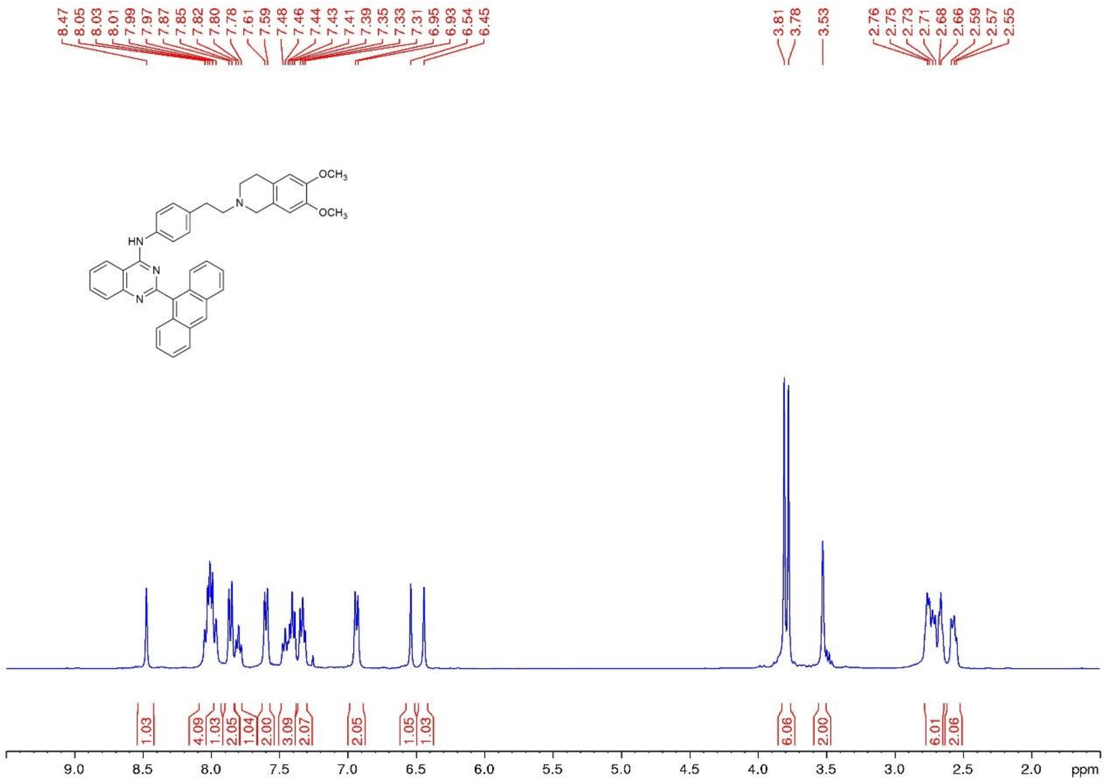

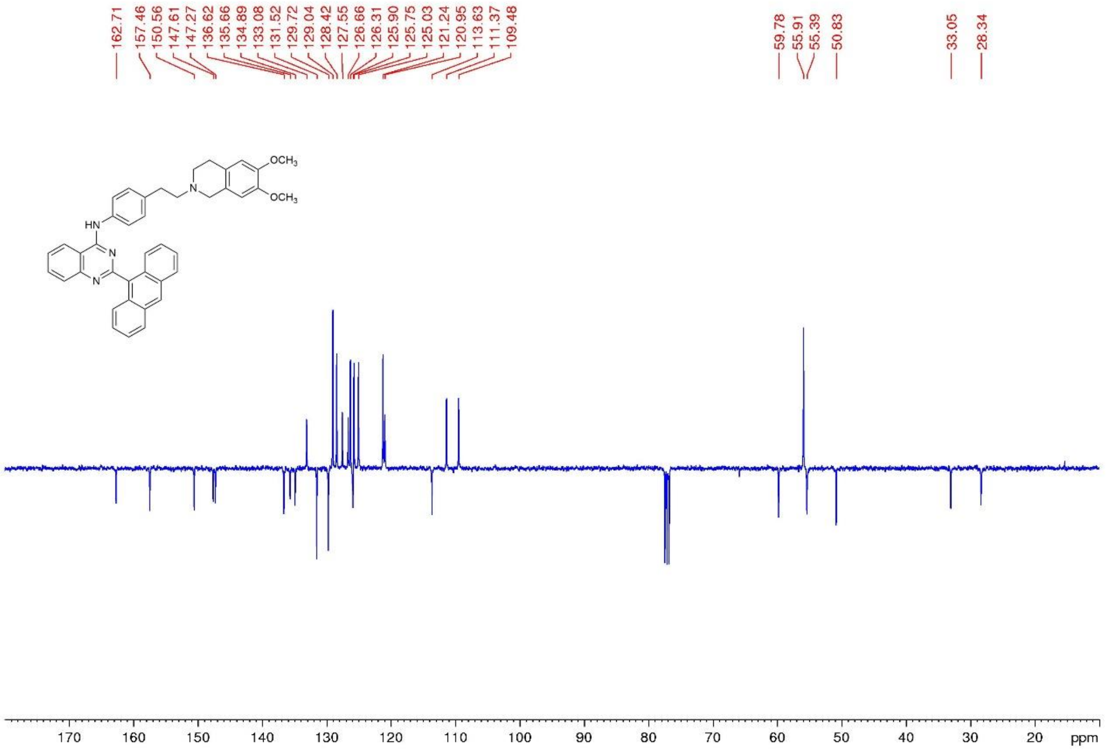

^{1}H-NMR and ^{13}C-APT-NMR spectra of compound **3b**

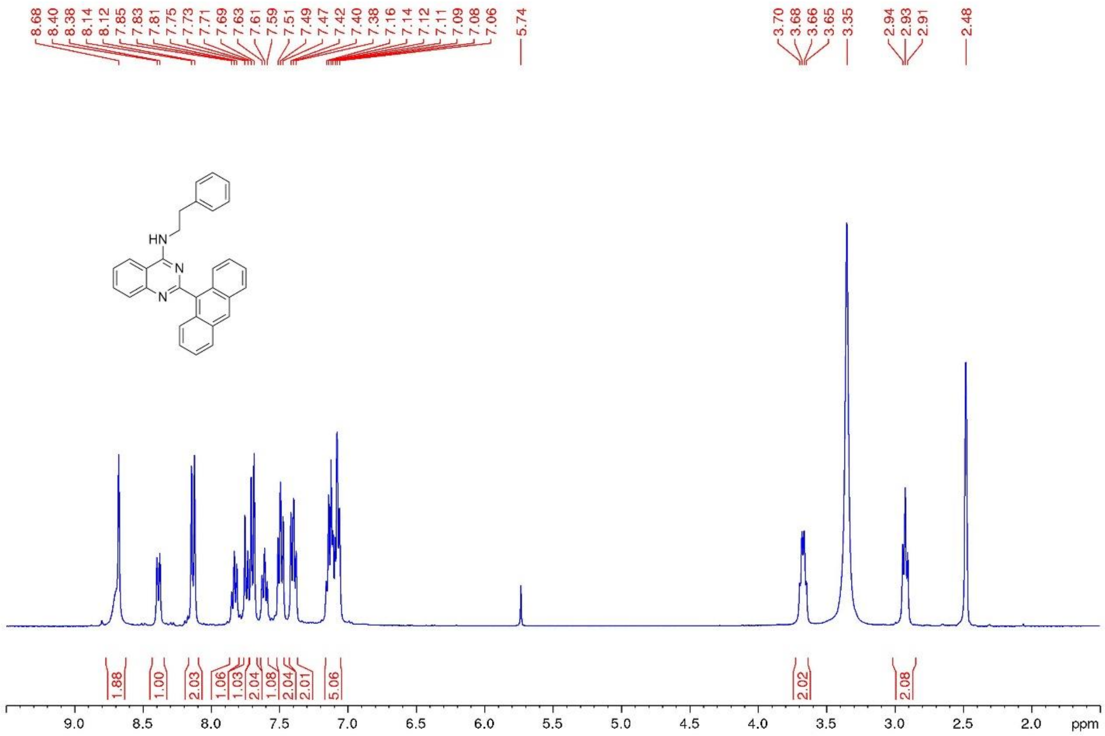

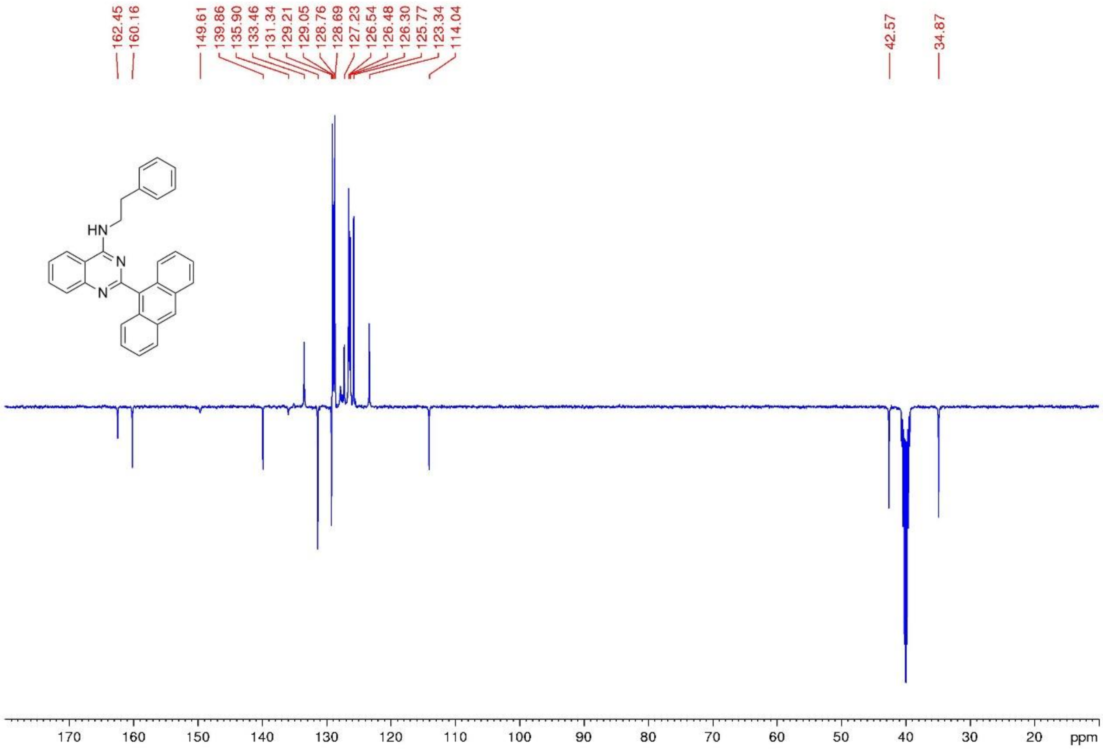

^{1}H-NMR and ^{13}C-APT-NMR spectra of compound **3c**

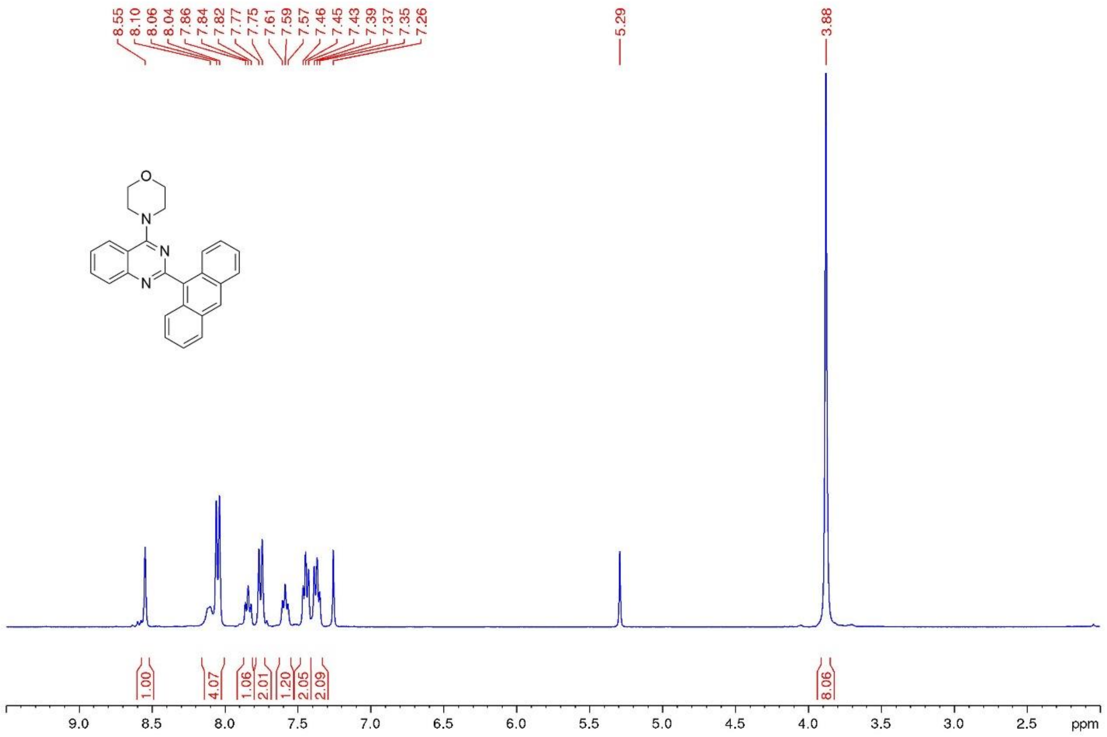

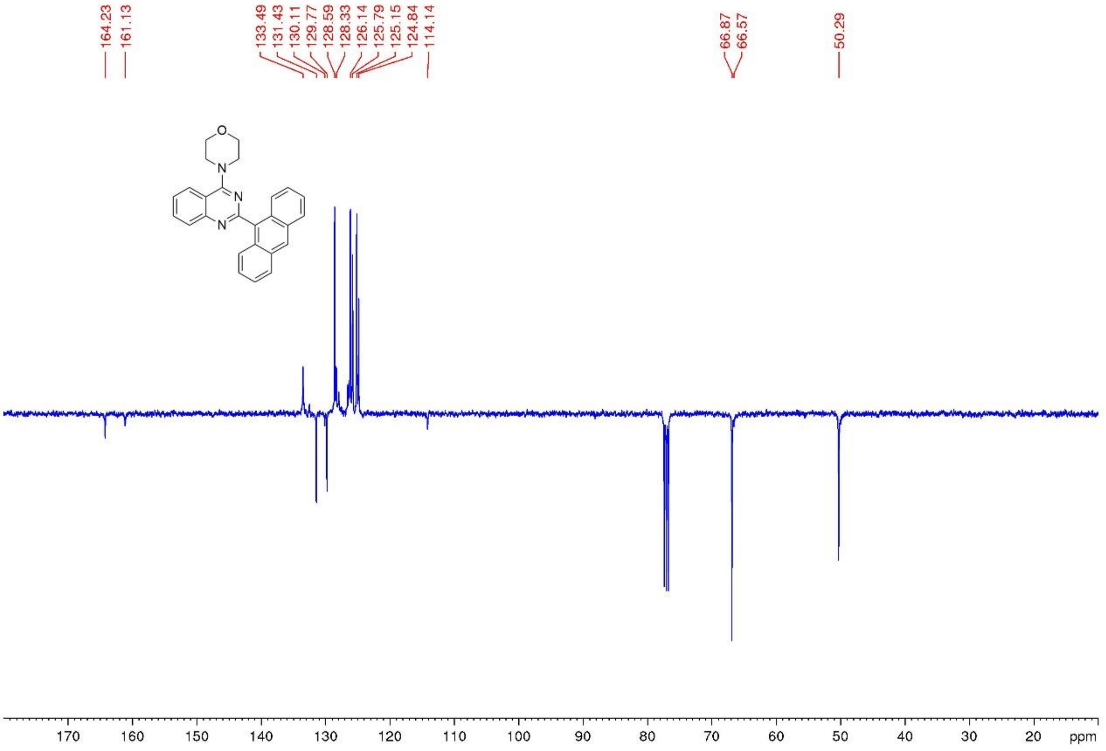

^{1}H-NMR and ^{13}C-APT-NMR spectra of compound **3d**

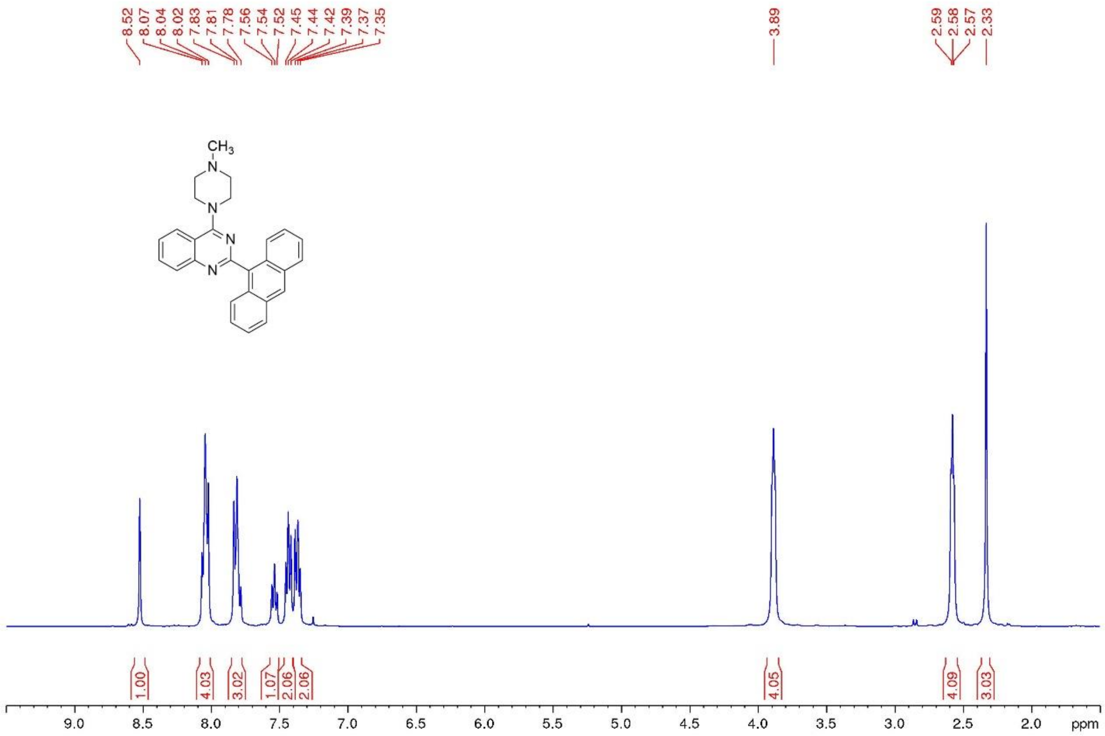

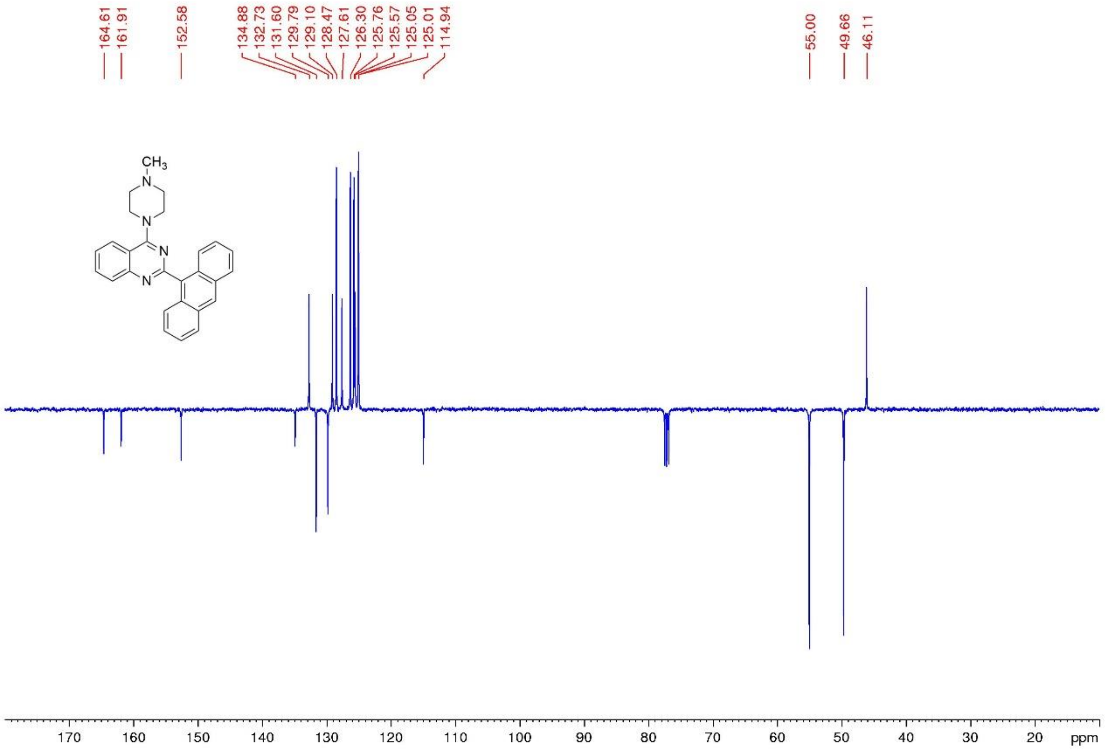

^{1}H-NMR and ^{13}C-APT-NMR spectra of compound **3e**

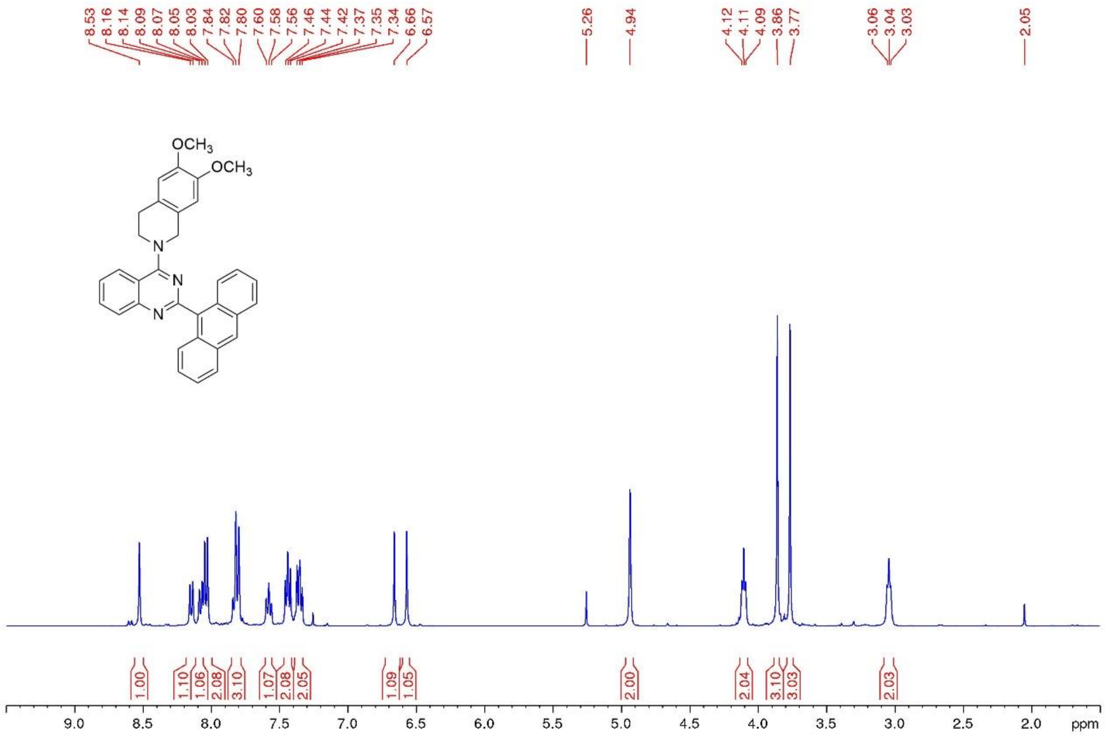

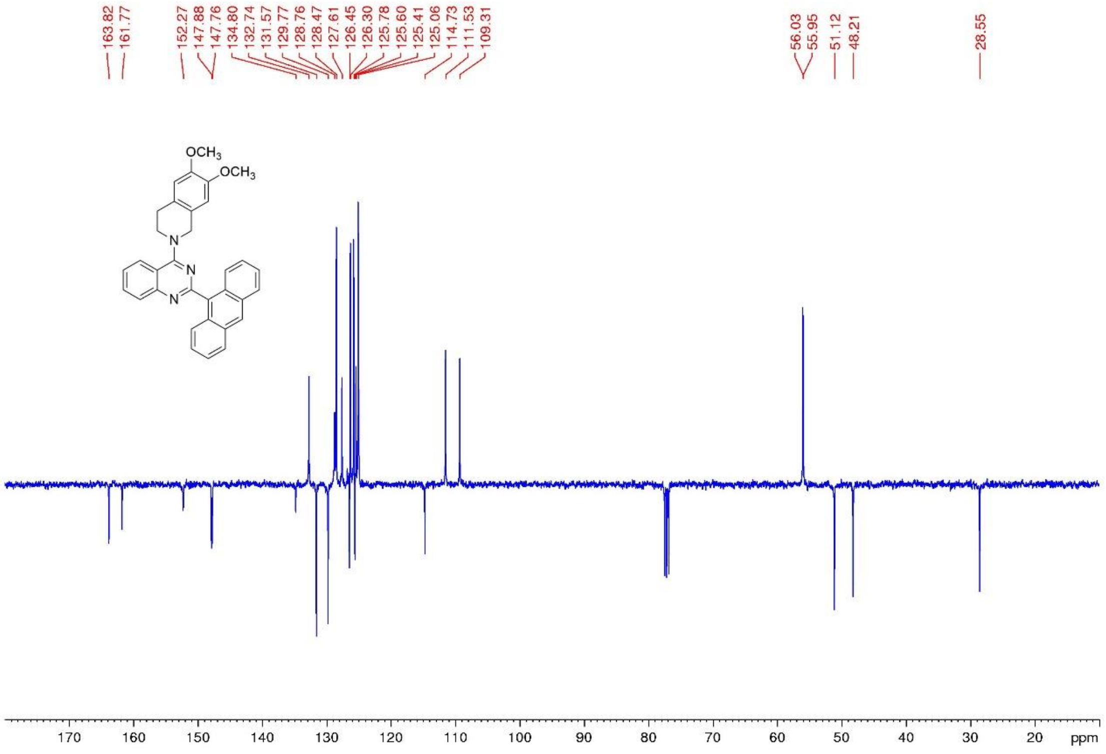

^{13}C-APT-NMR spectrum of compound **4a**

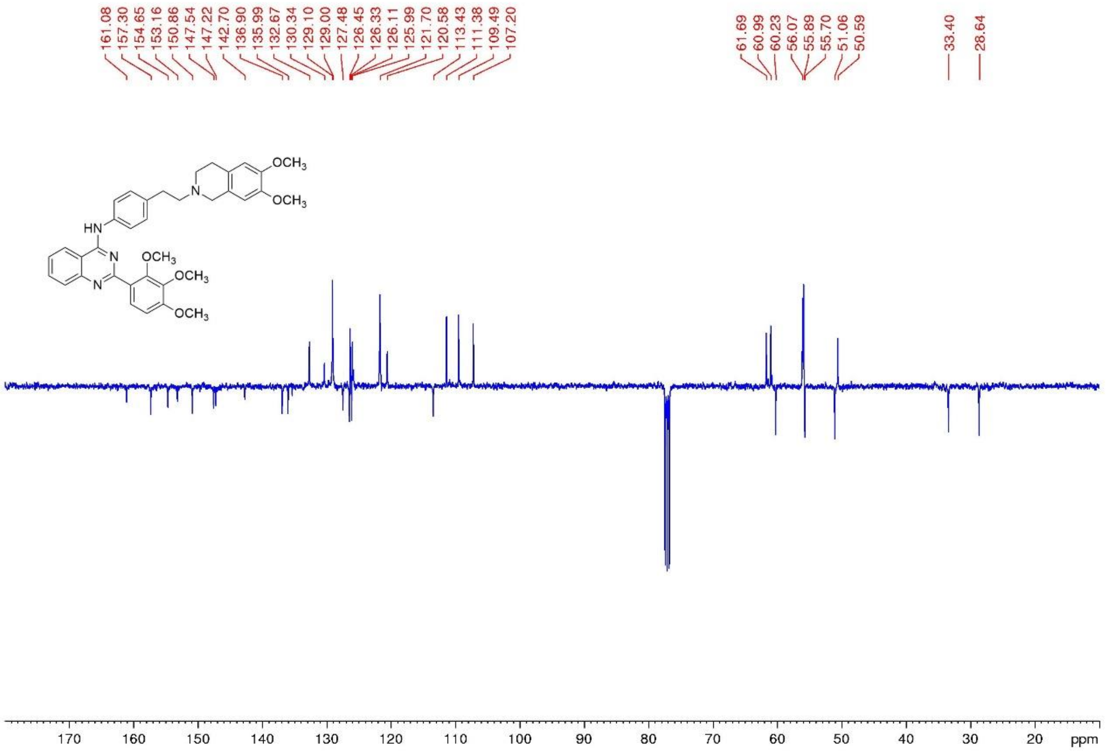

^{1}H-NMR and ^{13}C-APT-NMR spectra of compound **4b**

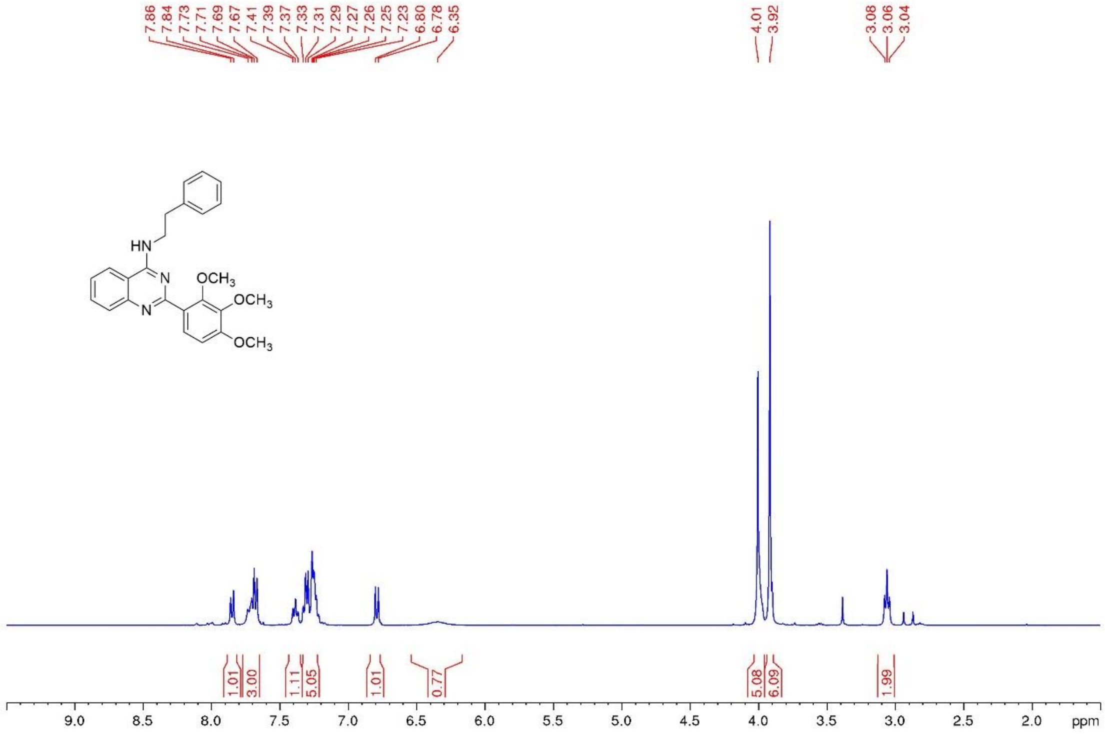

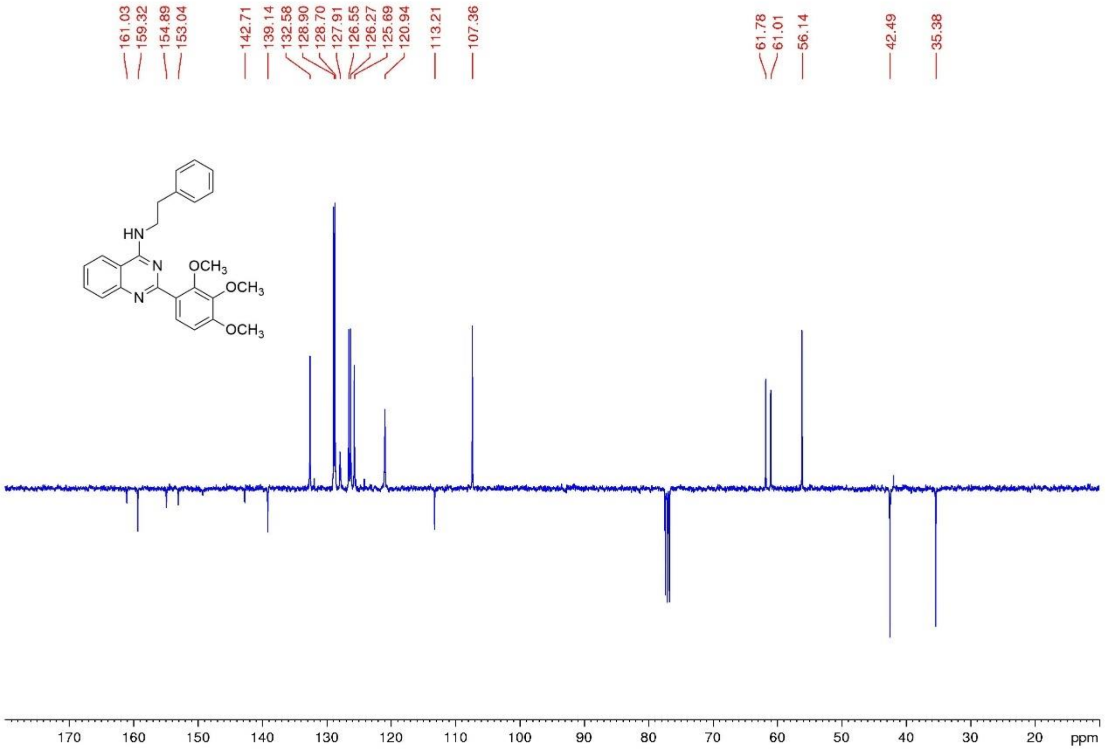

^{1}H-NMR and ^{13}C-APT-NMR spectra of compound **4c**

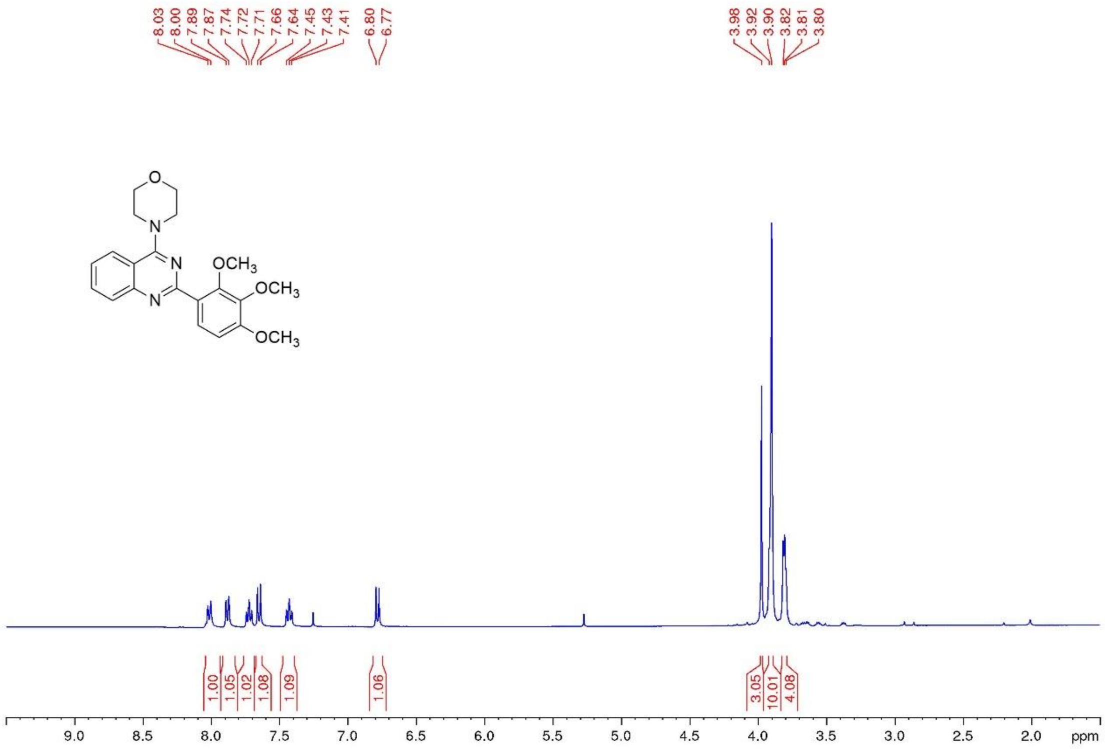

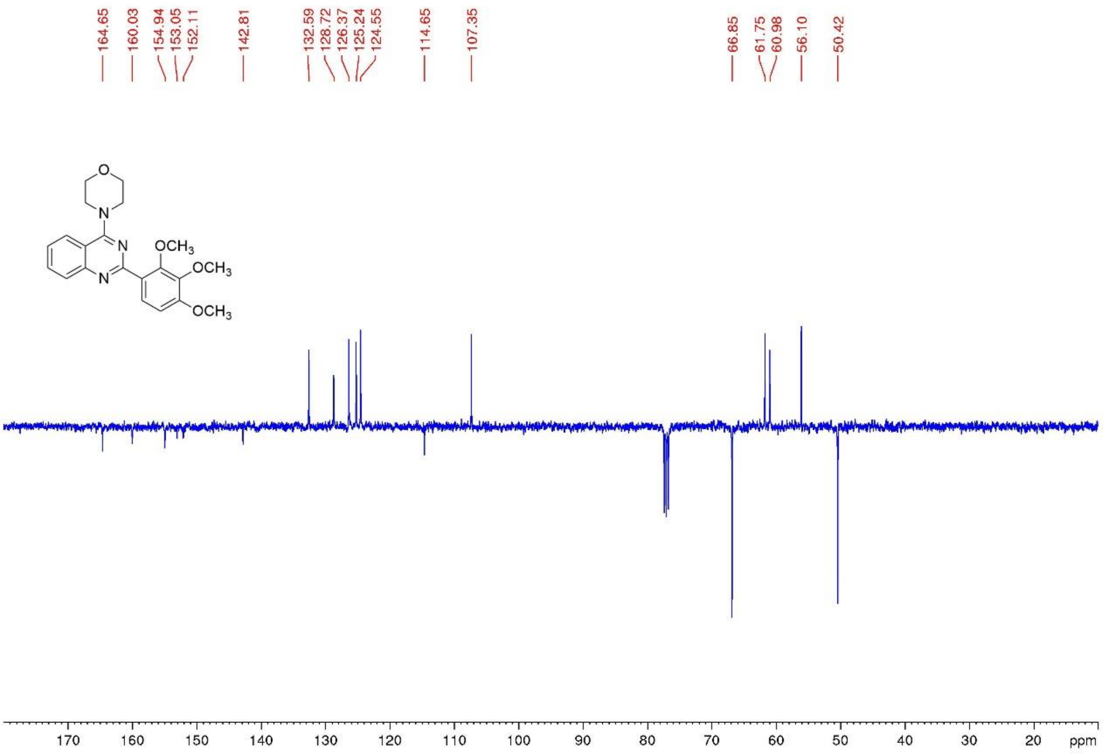

^{1}H-NMR and ^{13}C-APT-NMR spectra of compound **4d**

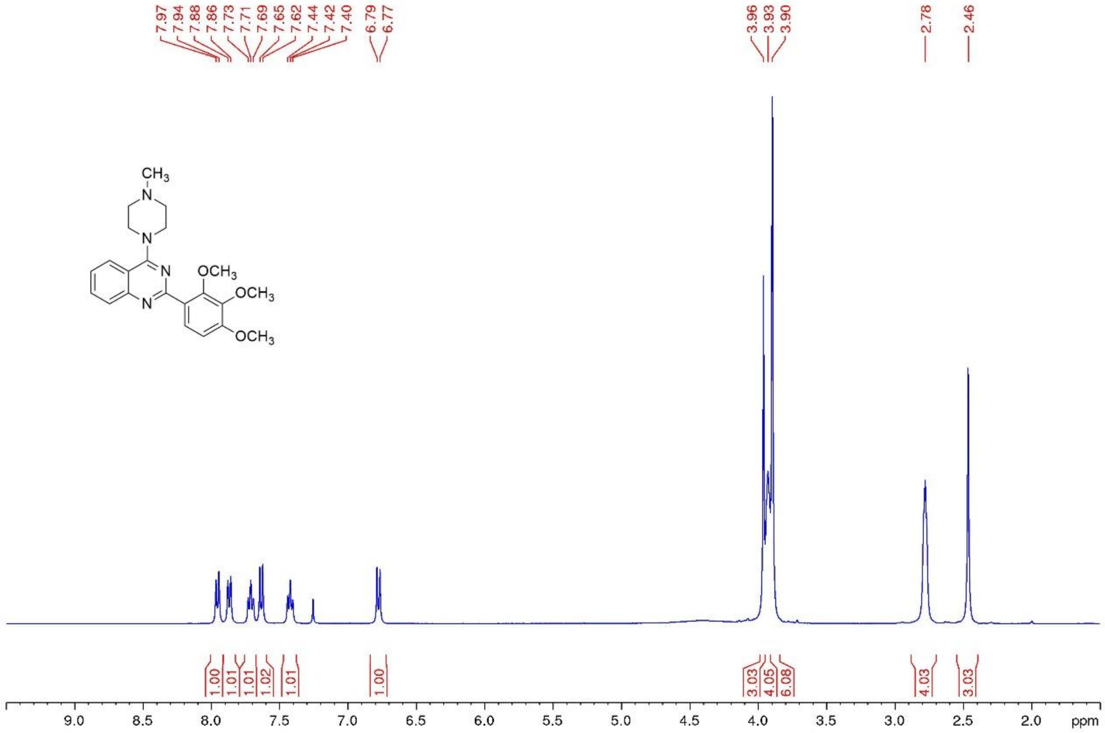

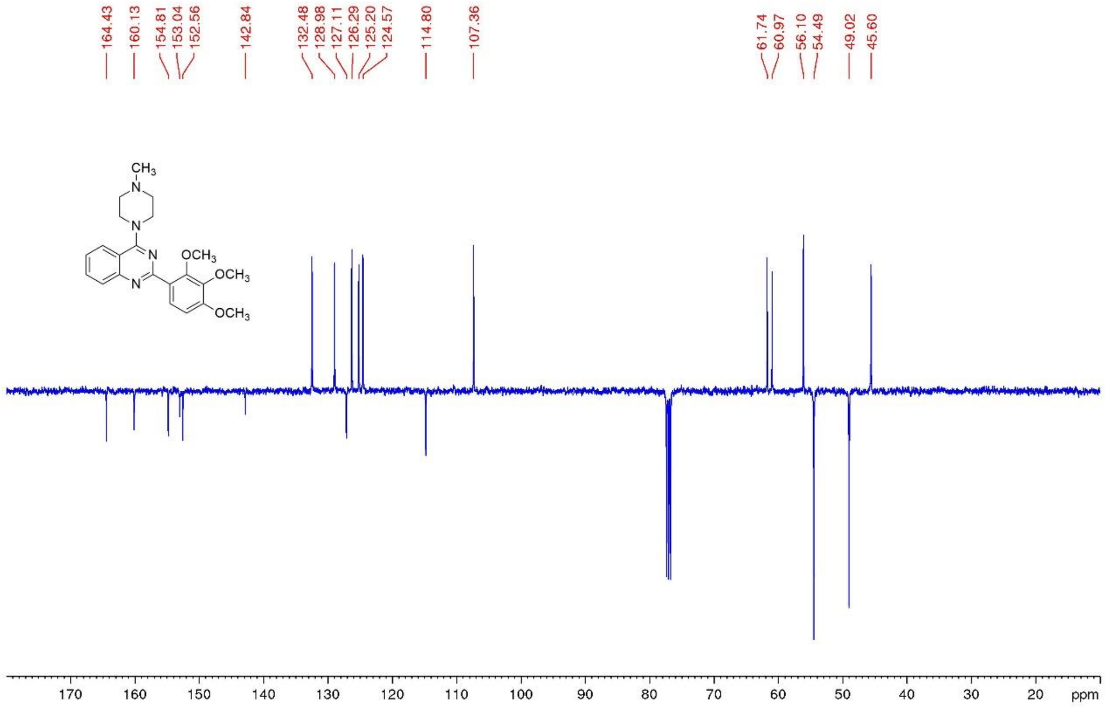

^{1}H-NMR and ^{13}C-APT-NMR spectra of compound **4e**

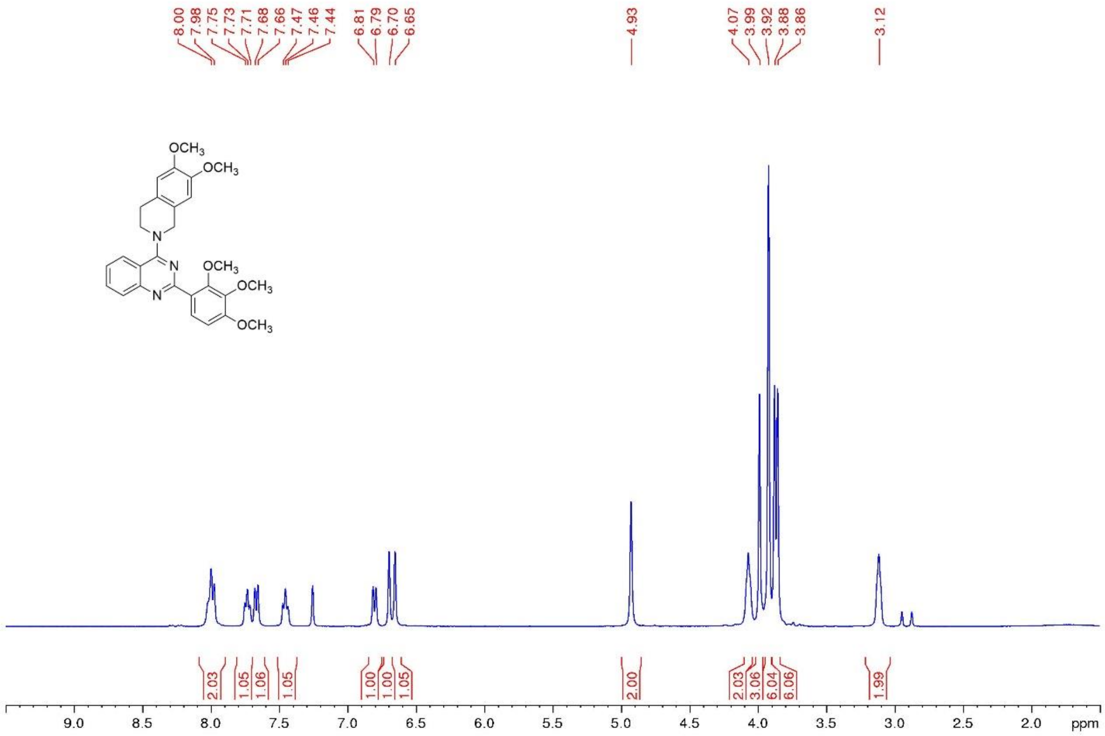

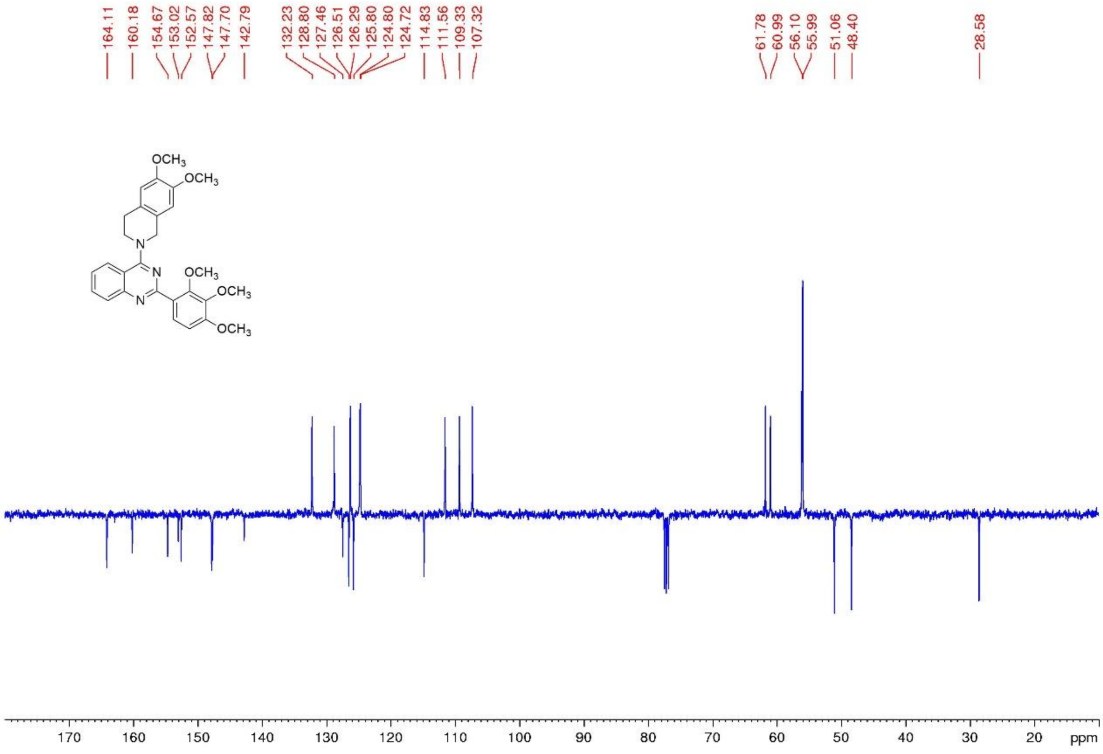

^{1}H-NMR and ^{13}C-APT-NMR spectra of compound **5a**

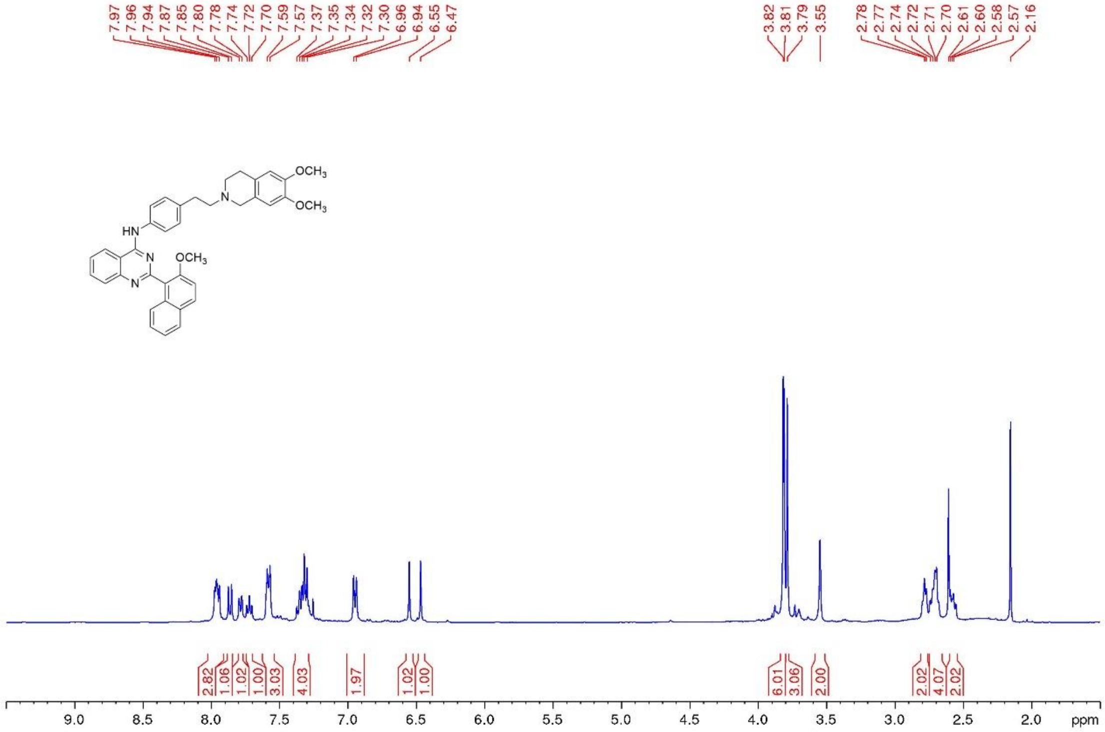

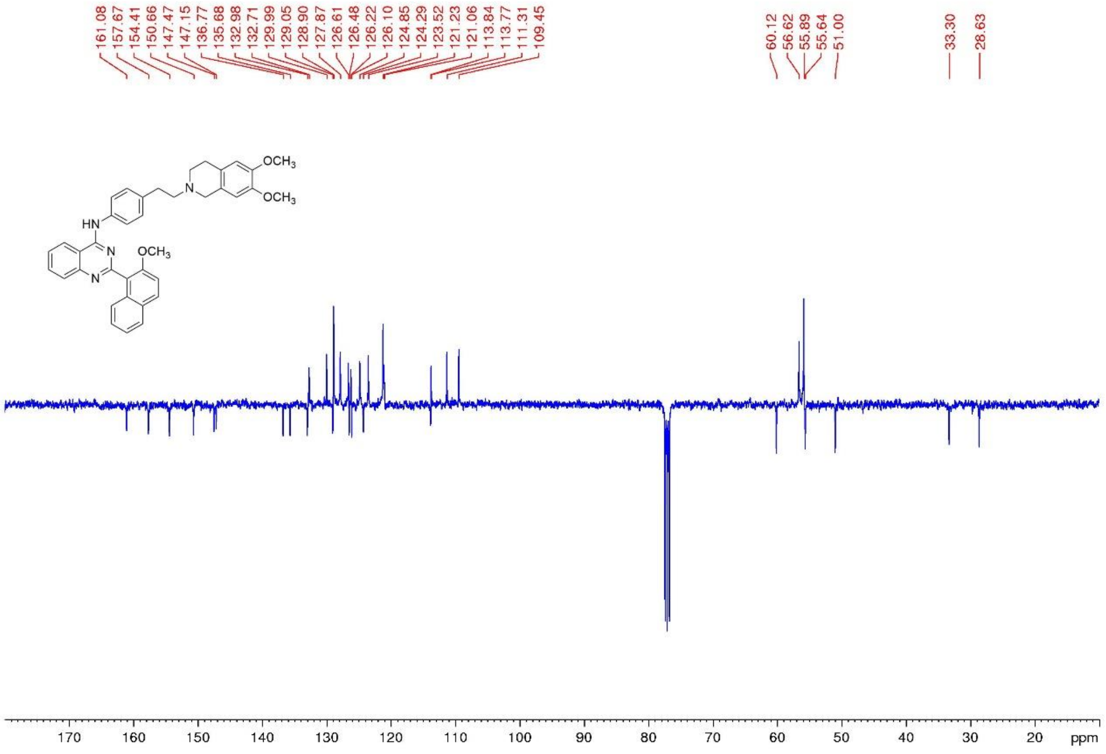

^{1}H-NMR and ^{13}C-APT-NMR spectra of compound **5b**

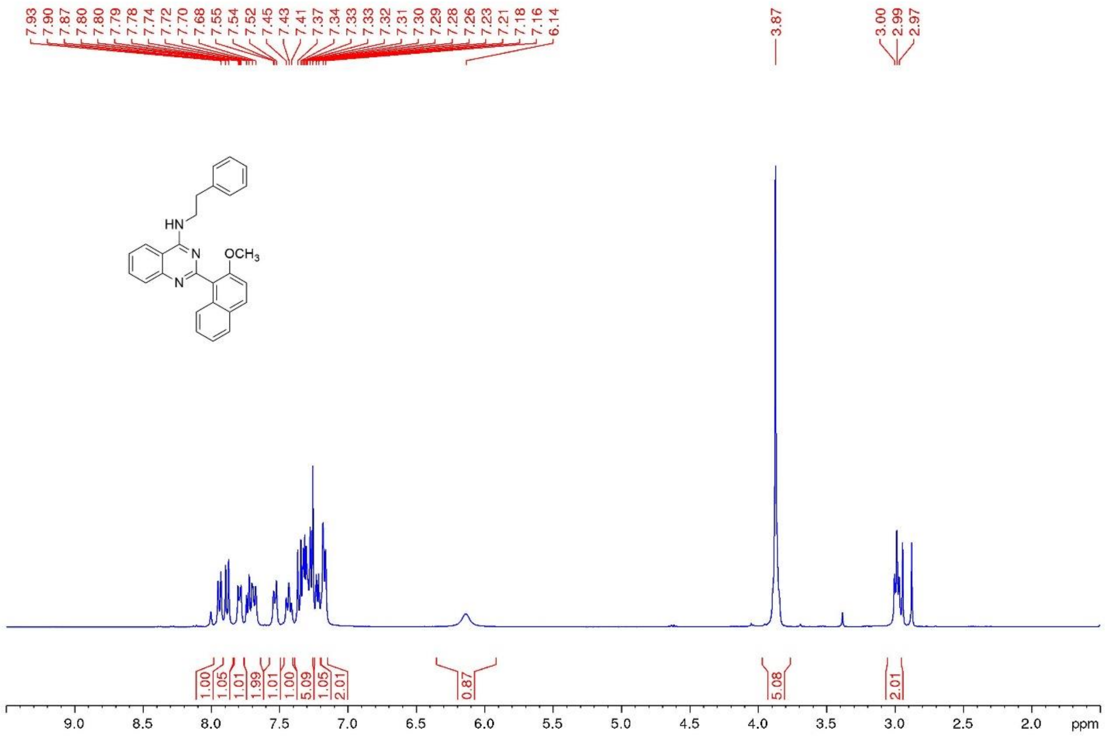

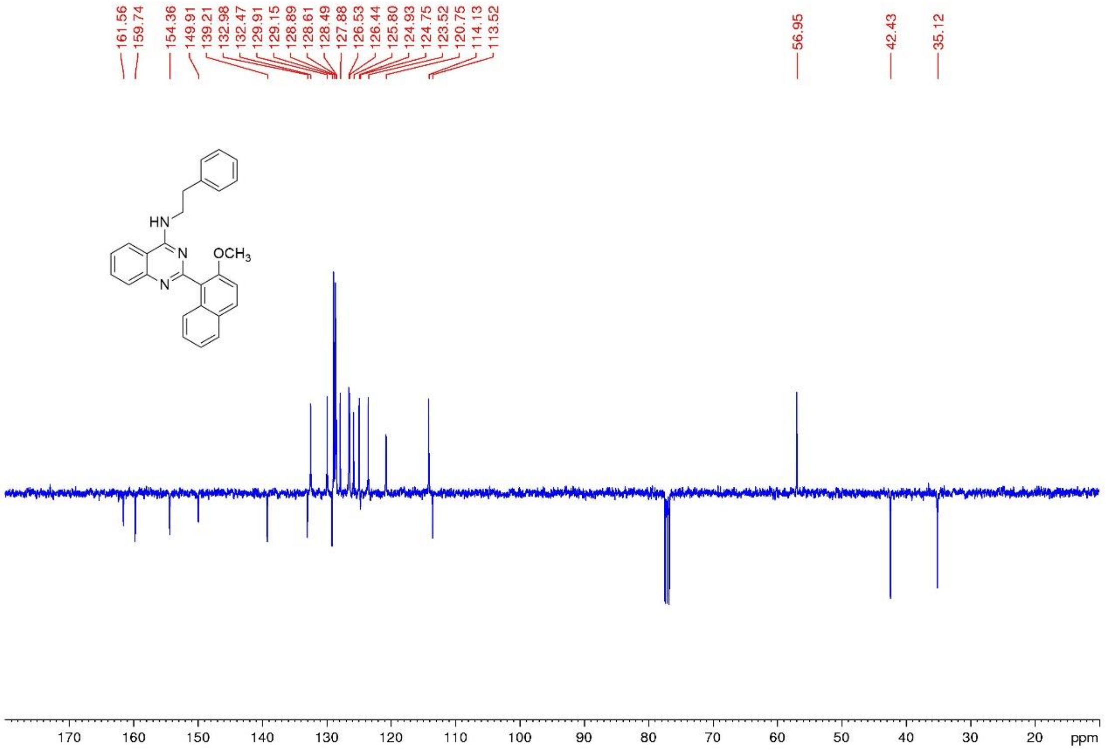

^{1}H-NMR and ^{13}C-APT-NMR spectra of compound **5c**

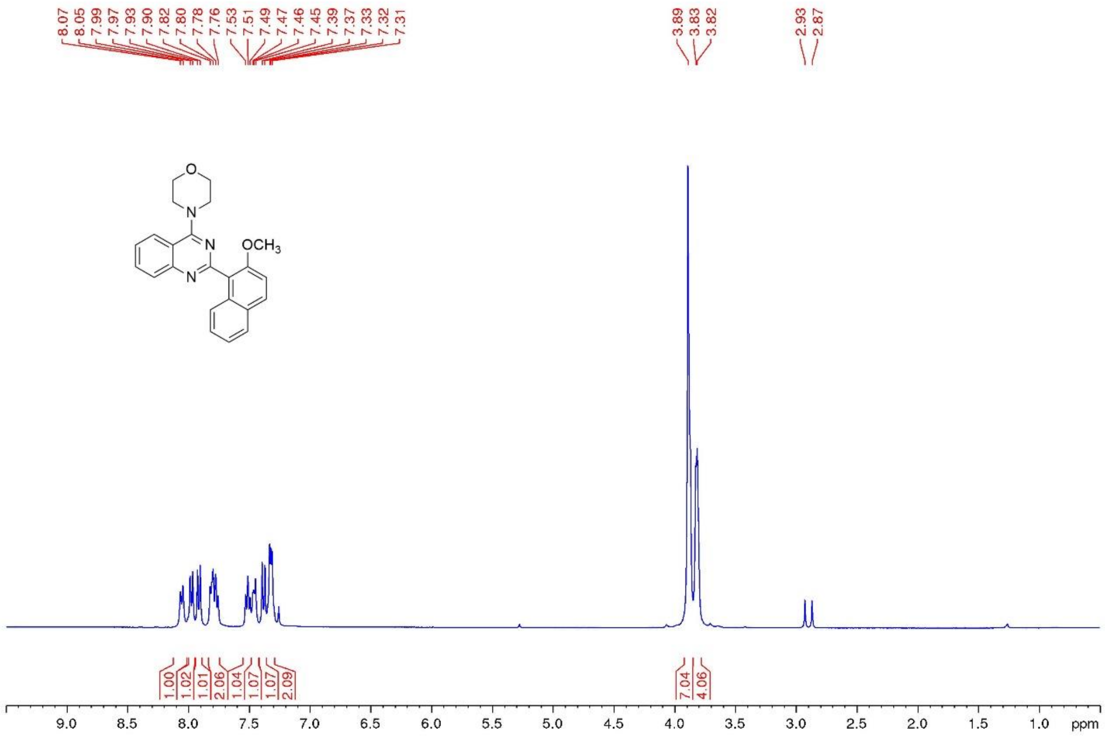

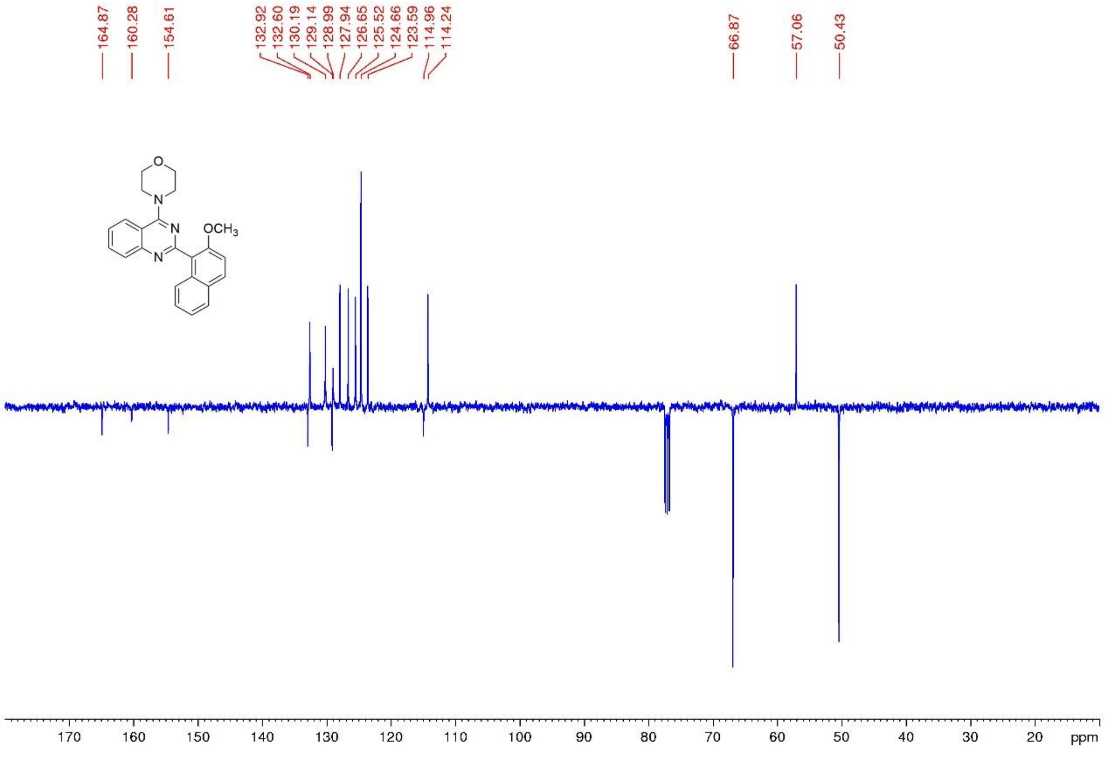

^{1}H-NMR and ^{13}C-APT-NMR spectra of compound **5d**

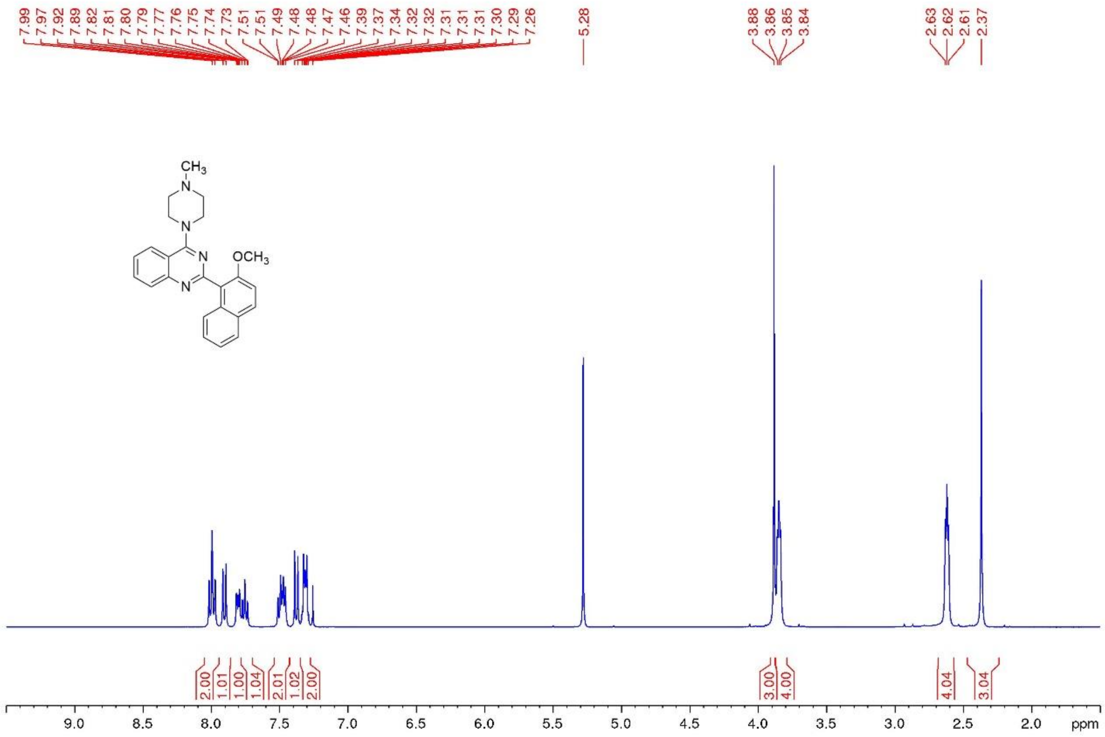

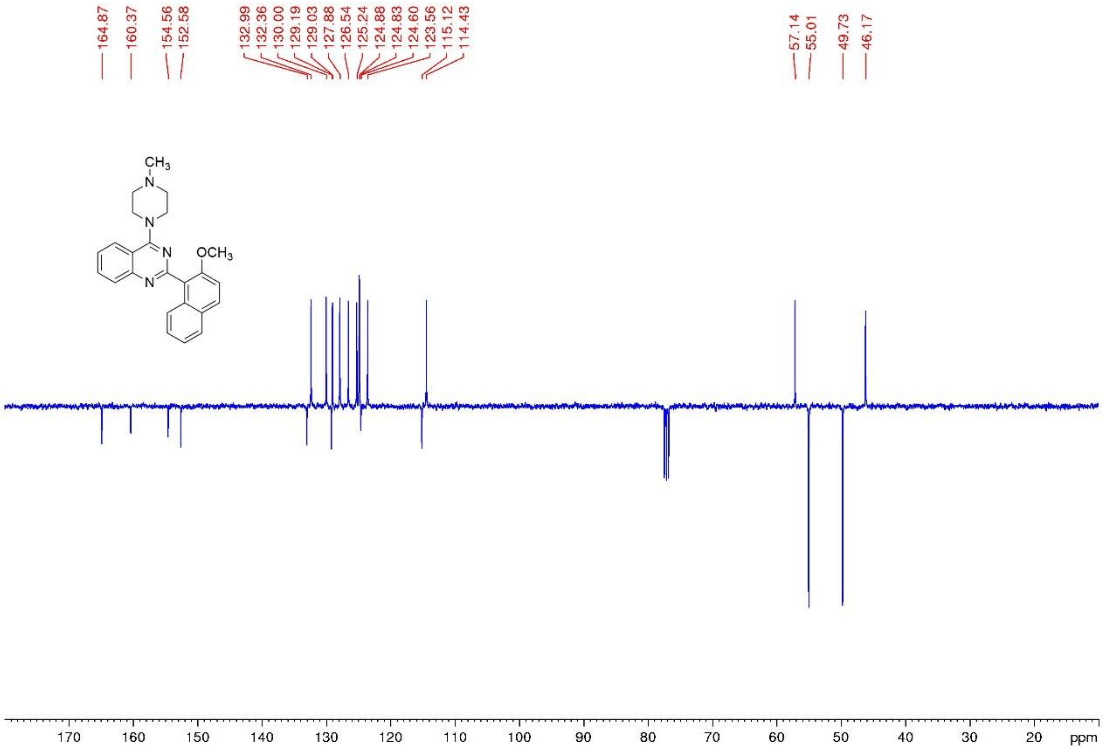

^{1}H-NMR and ^{13}C-APT-NMR spectra of compound **5e**

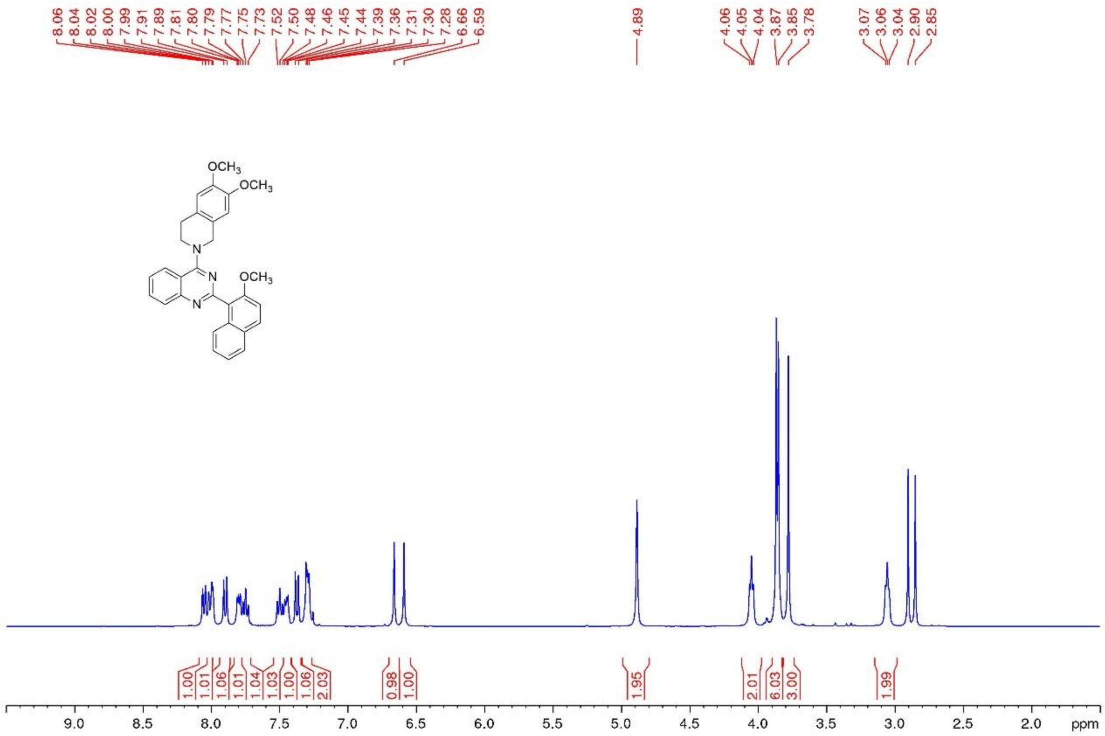

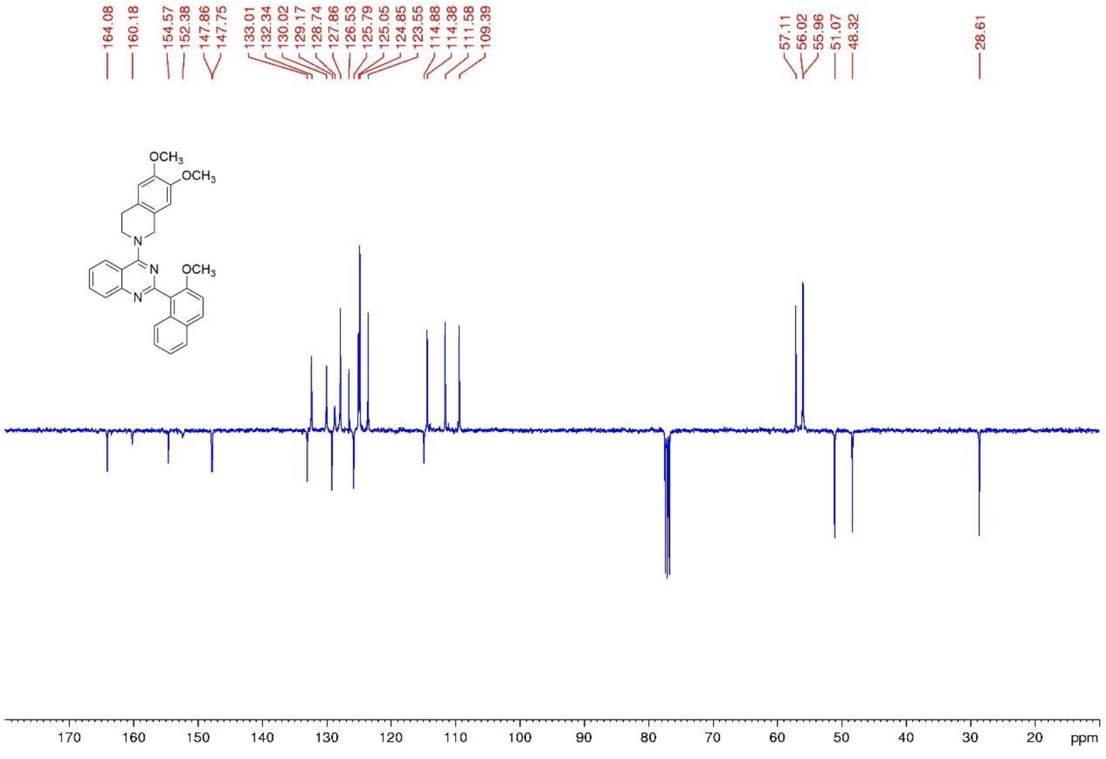

^{1}H-NMR and ^{13}C-APT-NMR spectra of compound **6a**

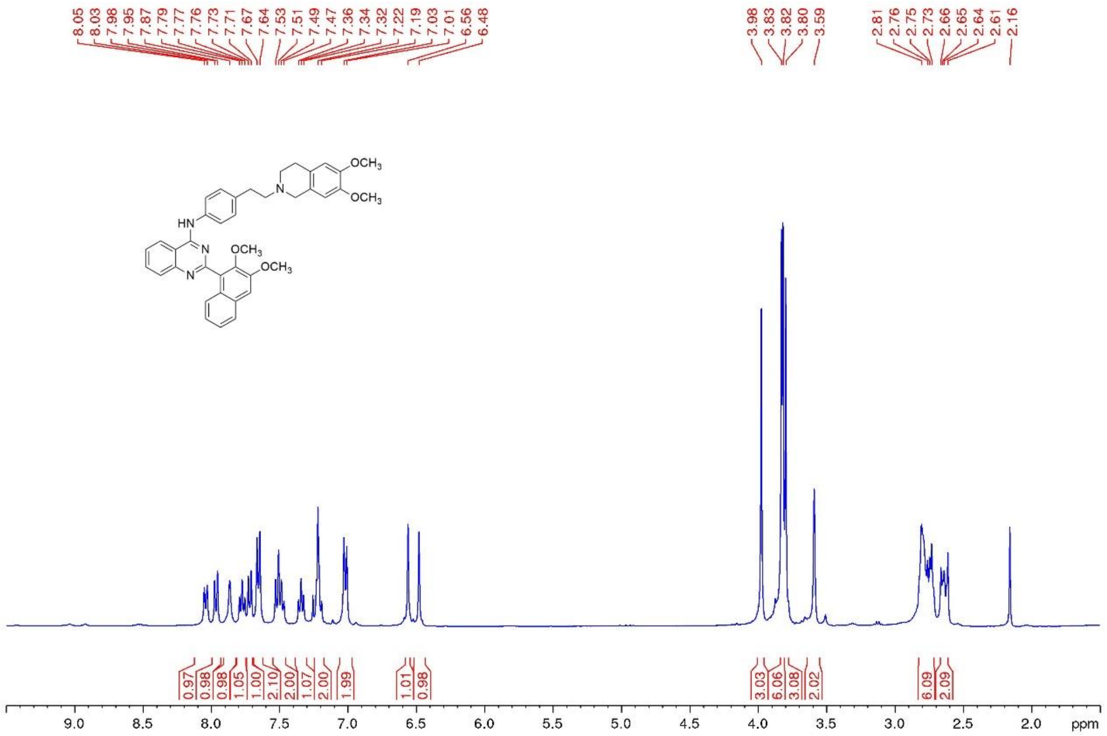

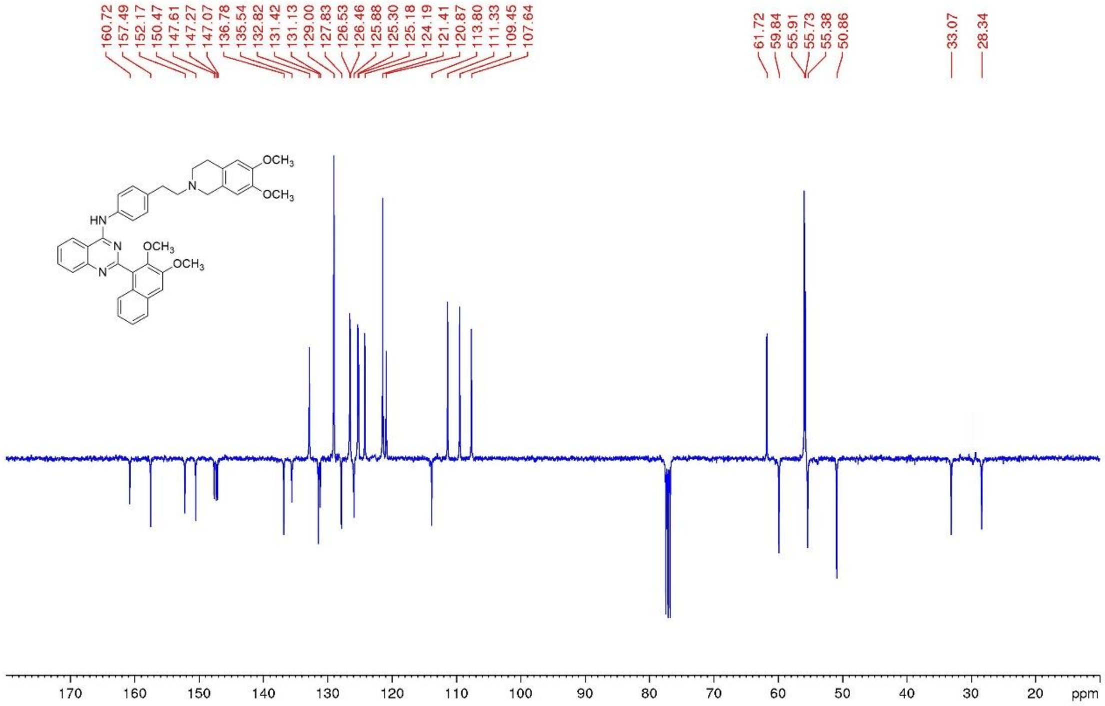

^{1}H-NMR and ^{13}C-APT-NMR spectra of compound **6b**

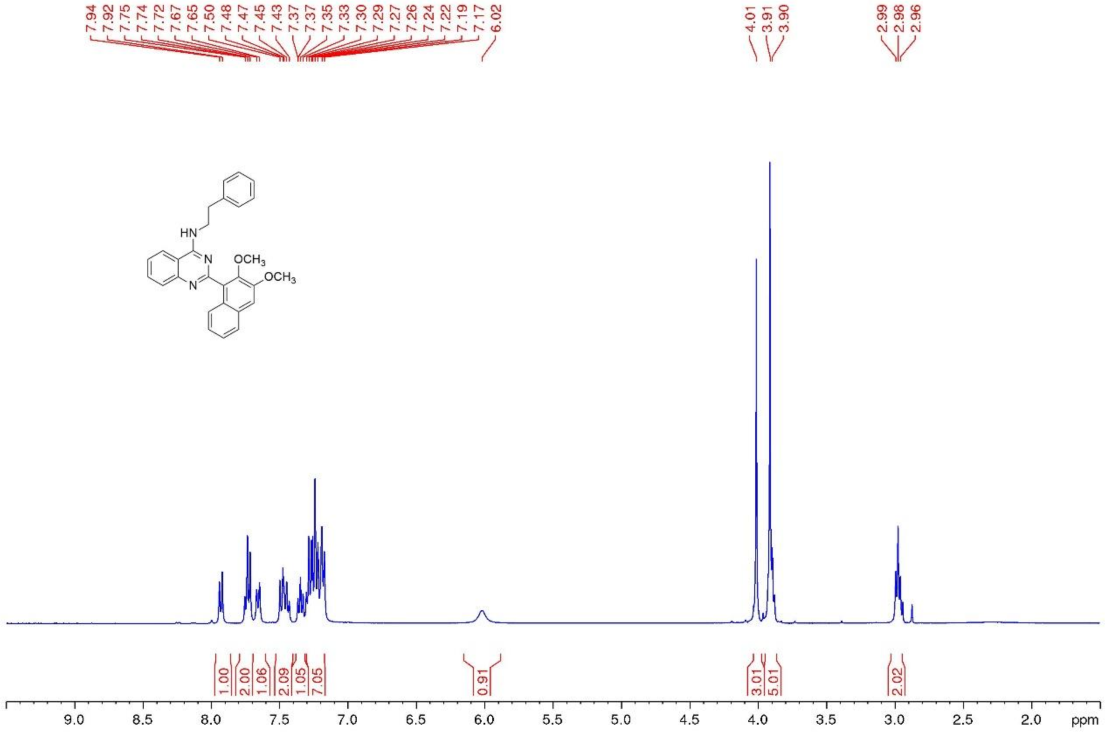

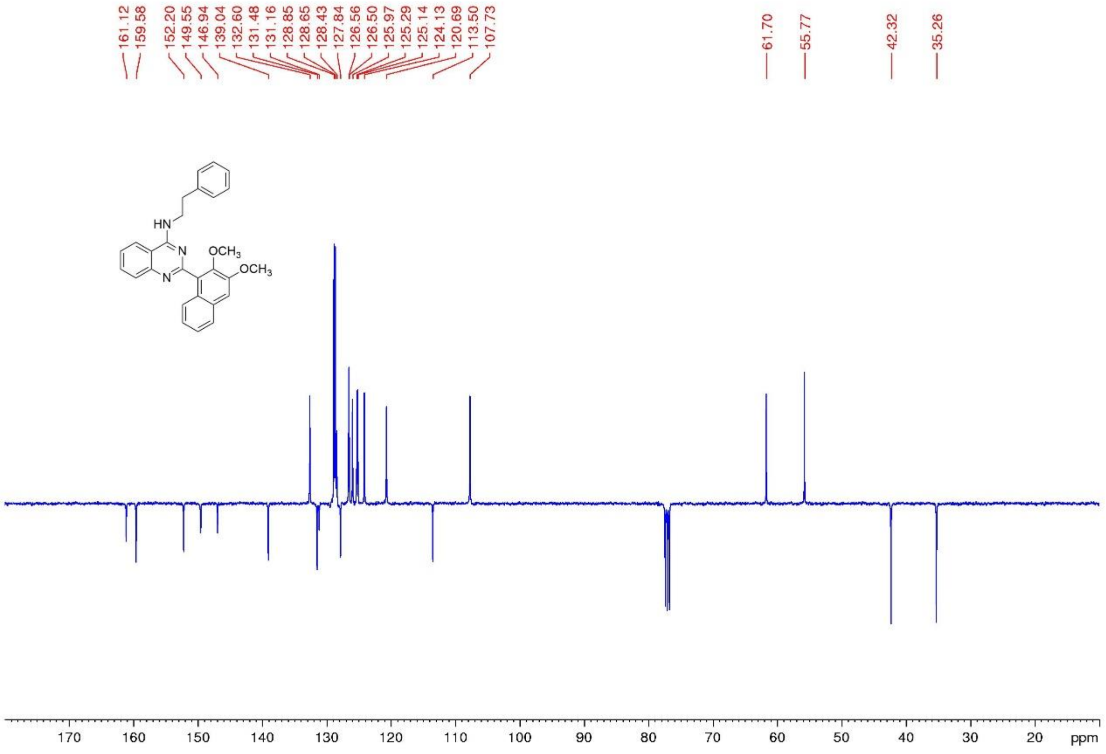

^{1}H-NMR and ^{13}C-APT-NMR spectra of compound **6c**

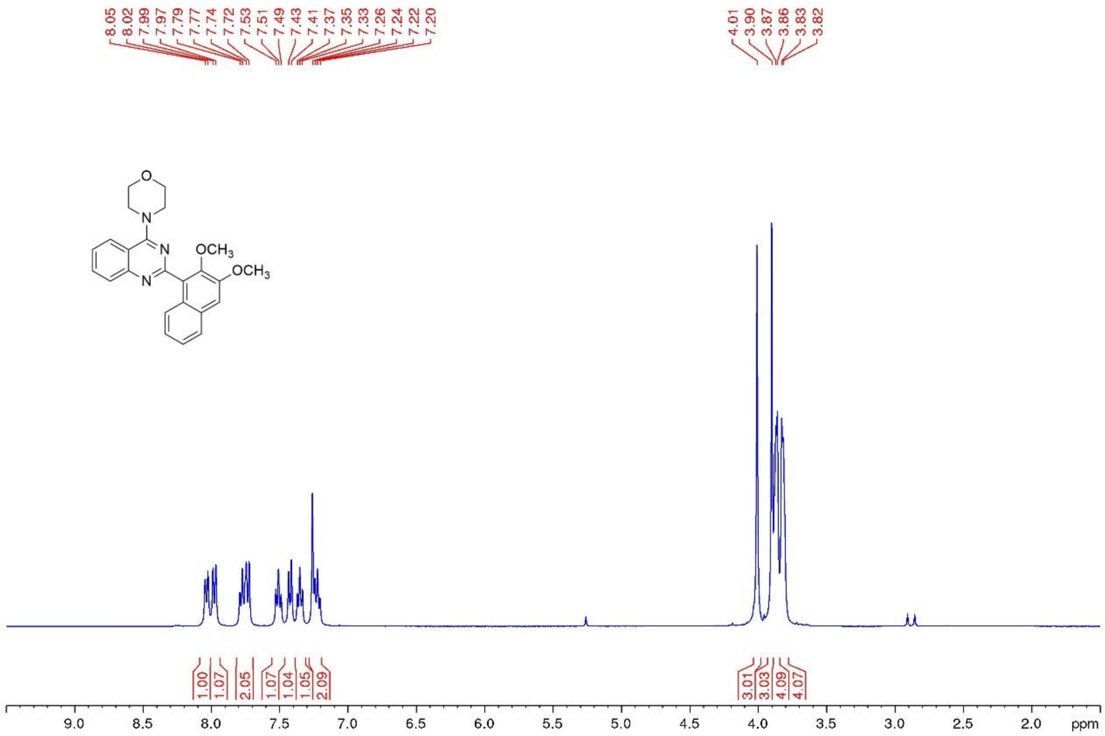

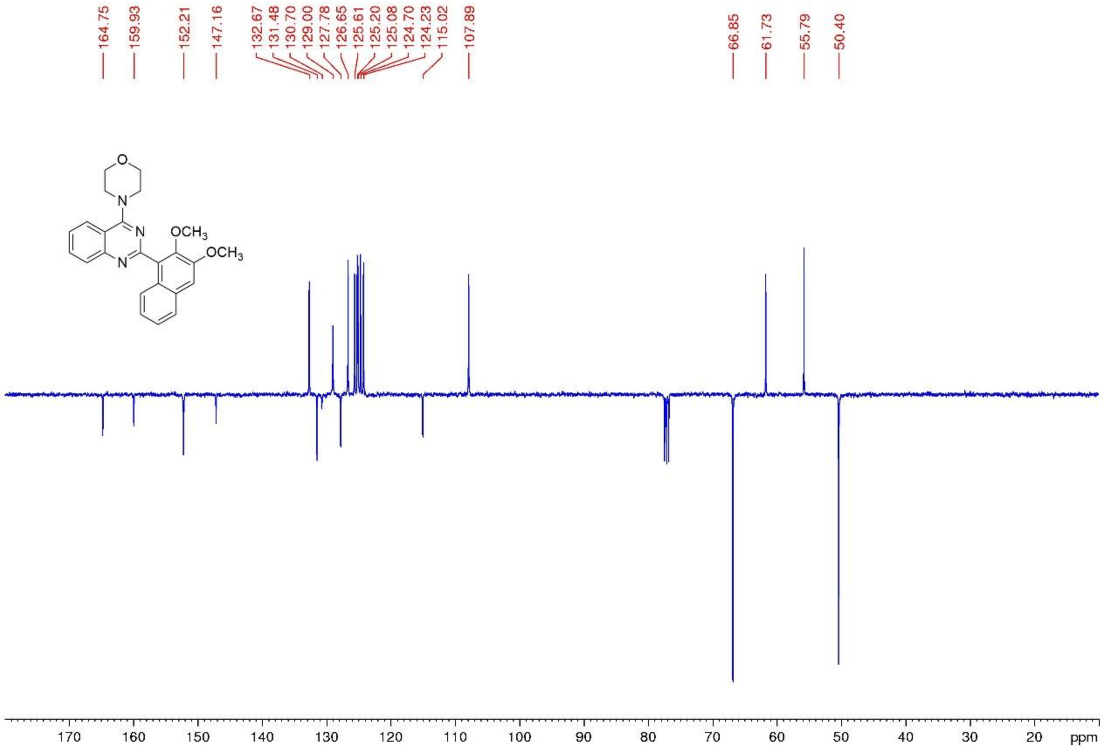

^{1}H-NMR and ^{13}C-APT-NMR spectra of compound **6d**

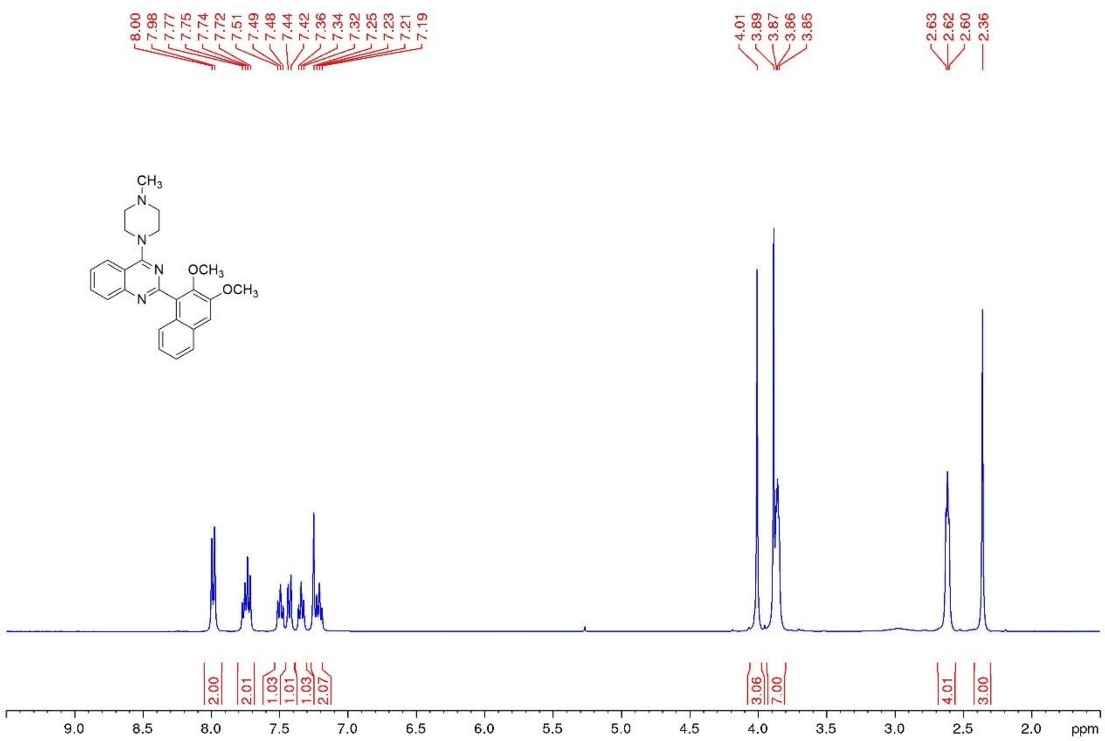

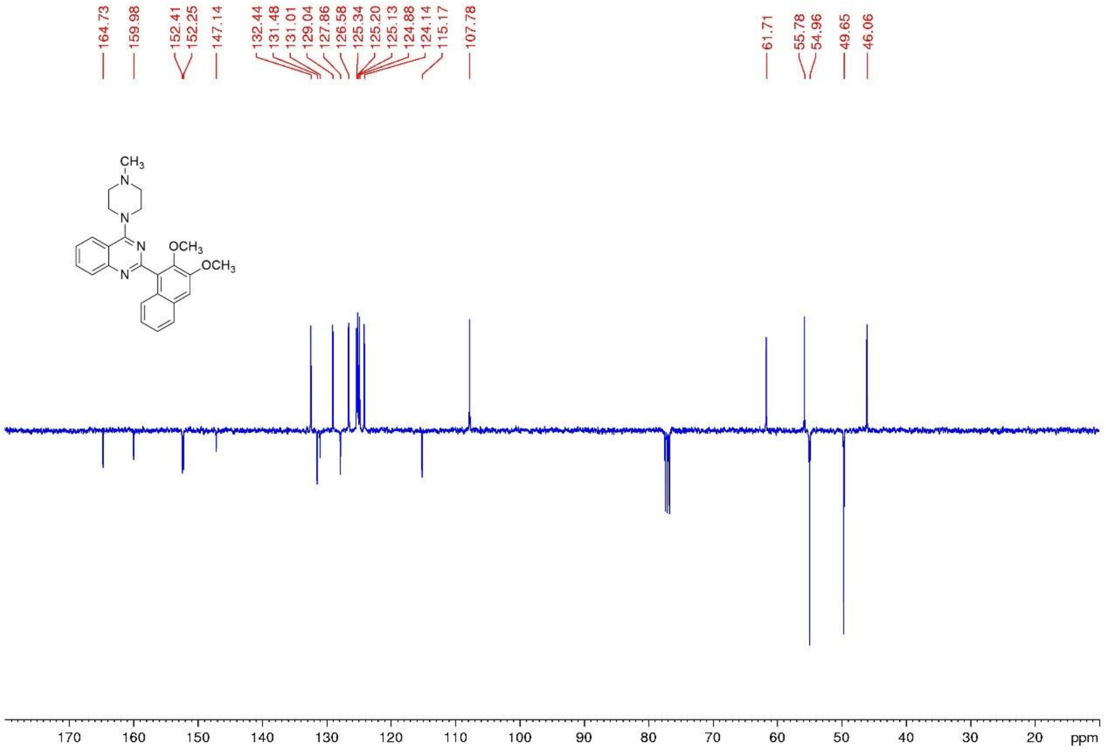

^{1}H-NMR and ^{13}C-APT-NMR spectra of compound **6e**

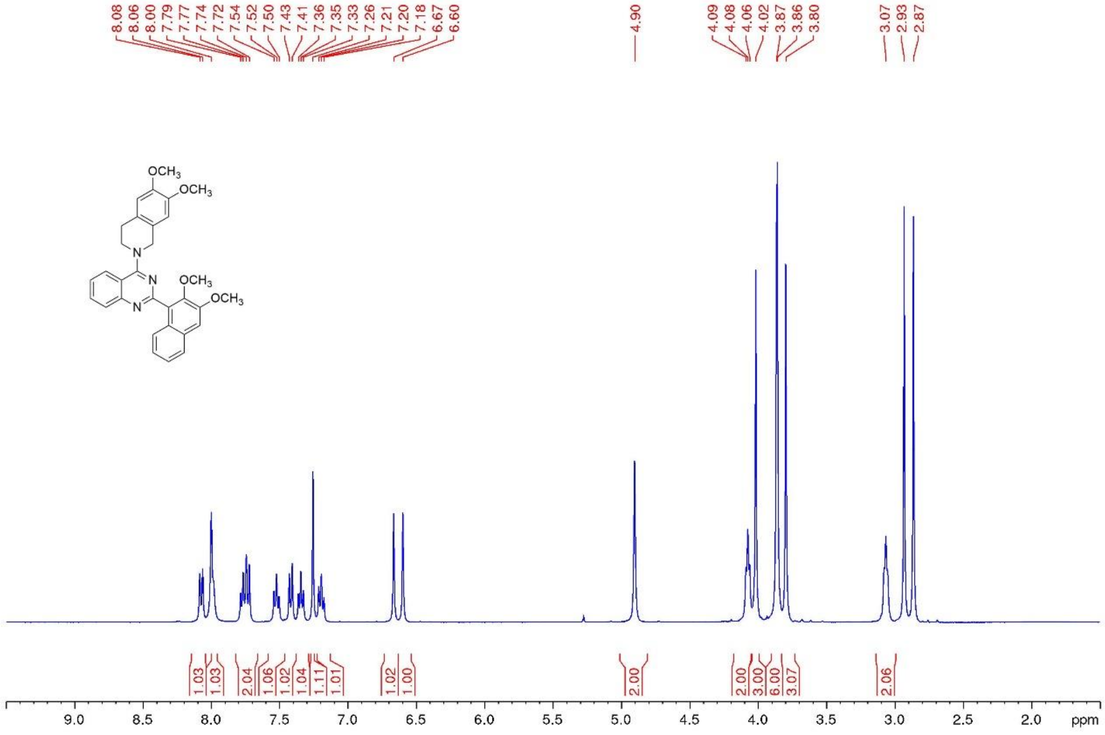

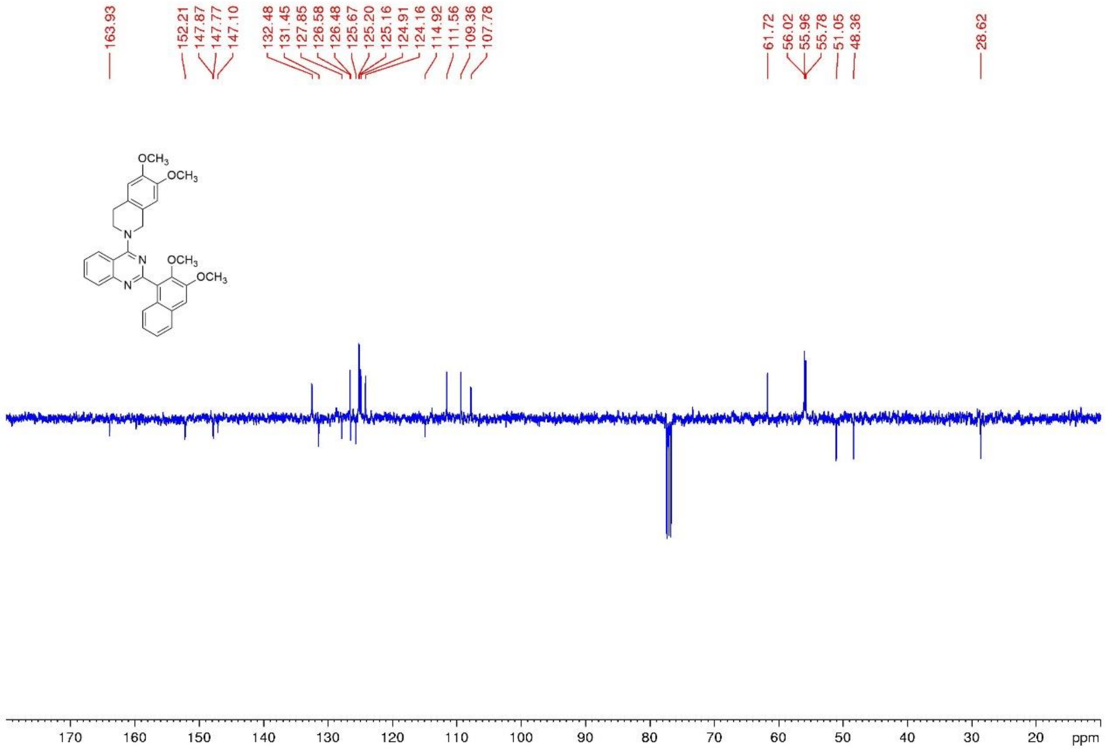

^{1}H-NMR and ^{13}C-APT-NMR spectra of compound **7a**

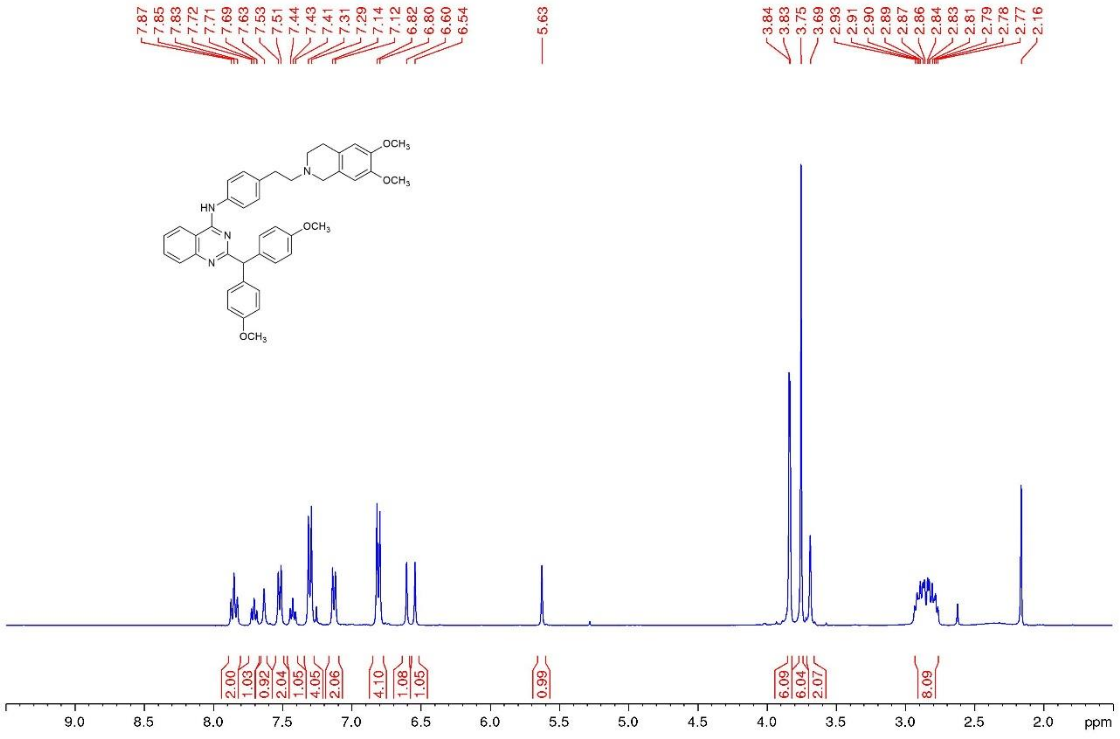

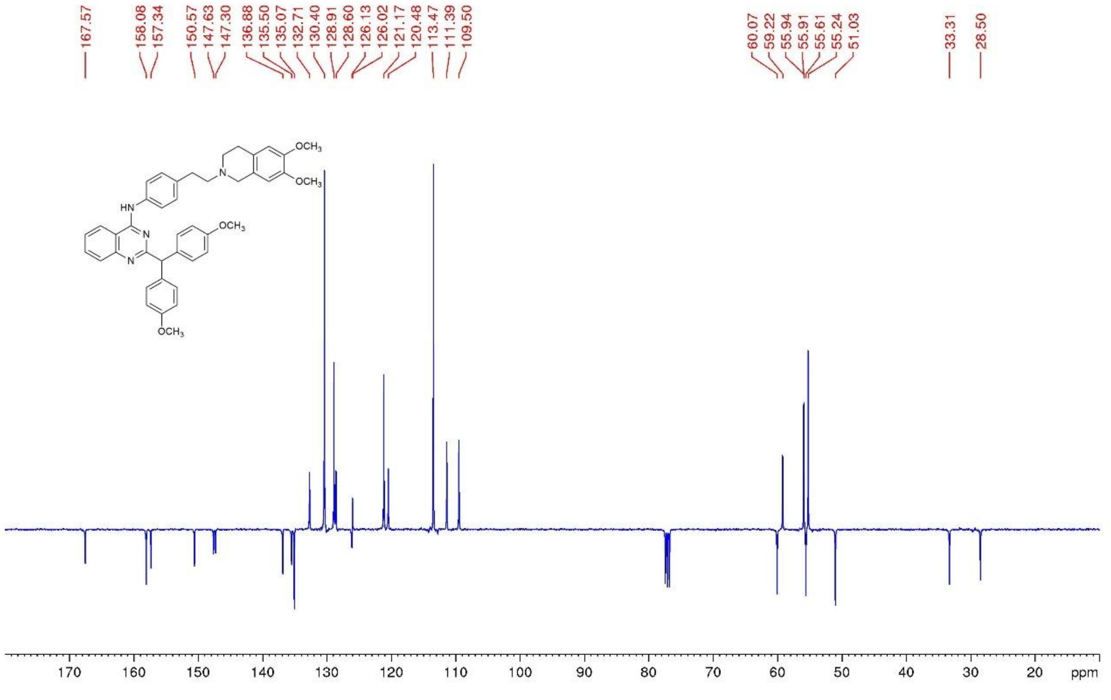

^{1}H-NMR and ^{13}C-APT-NMR spectra of compound **7b**

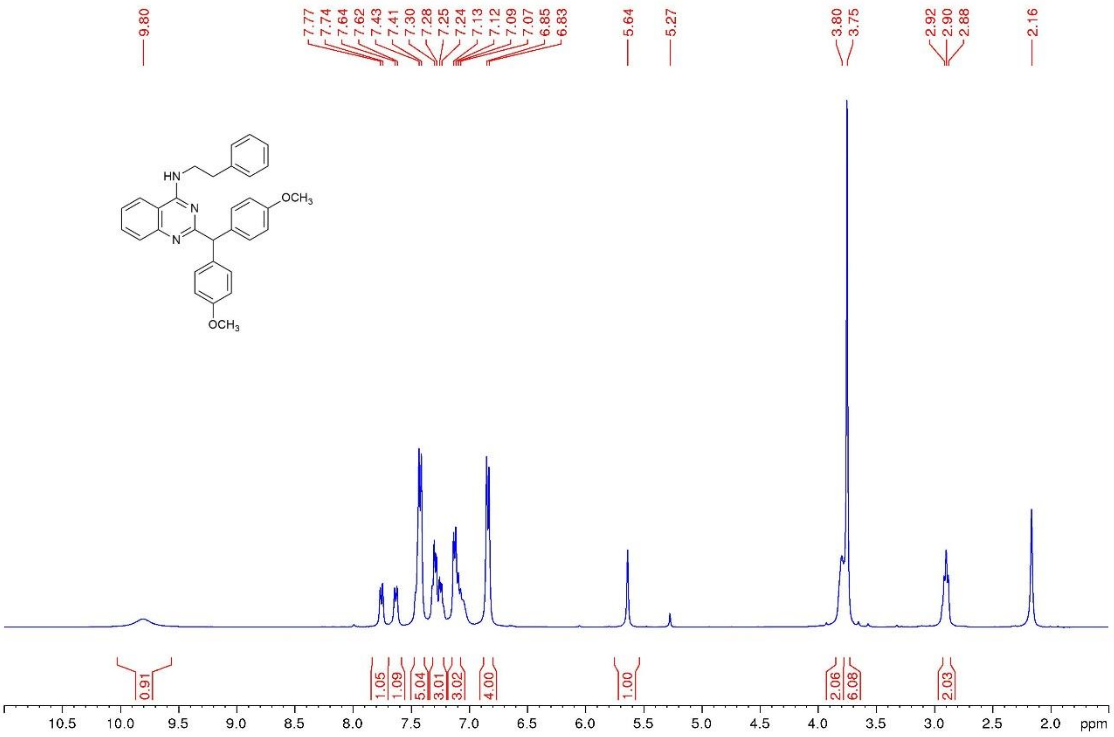

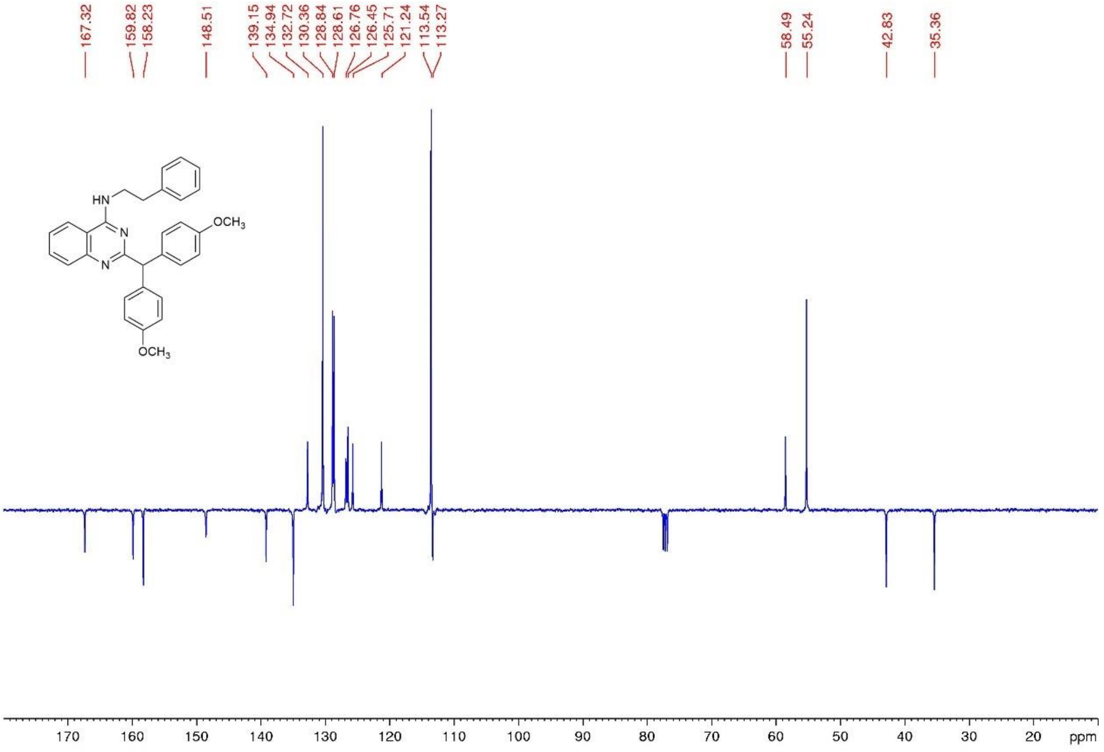

^{1}H-NMR and ^{13}C-APT-NMR spectra of compound **7c**

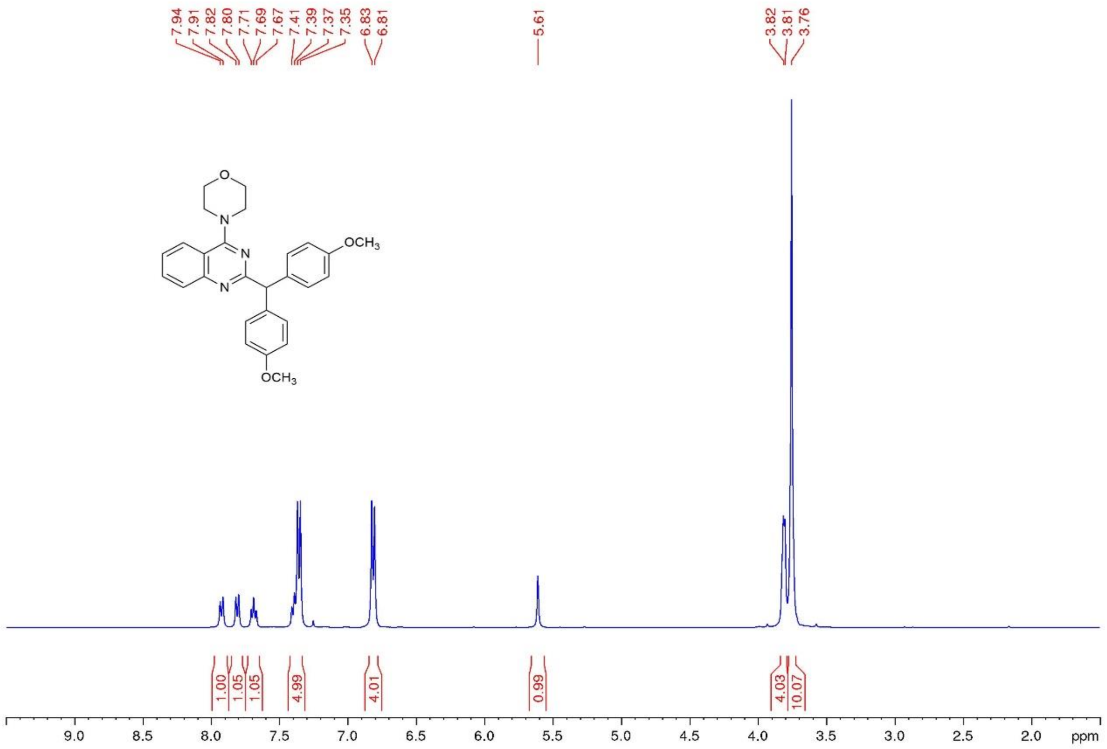

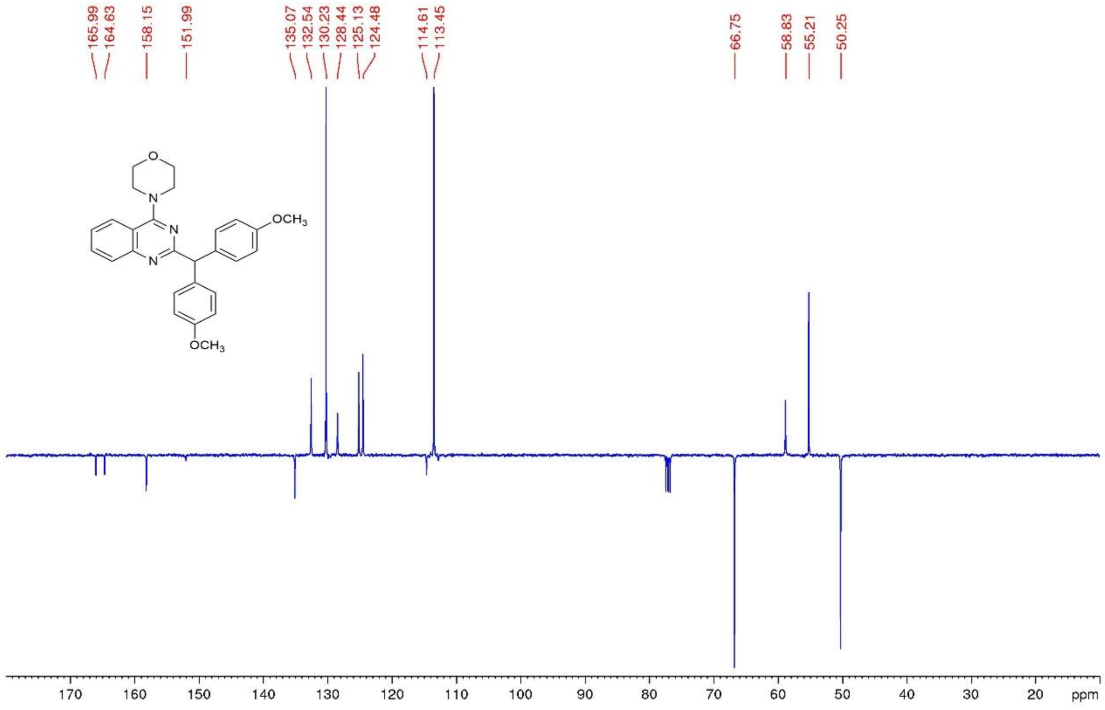

^{1}H-NMR and ^{13}C-APT-NMR spectra of compound **7d**

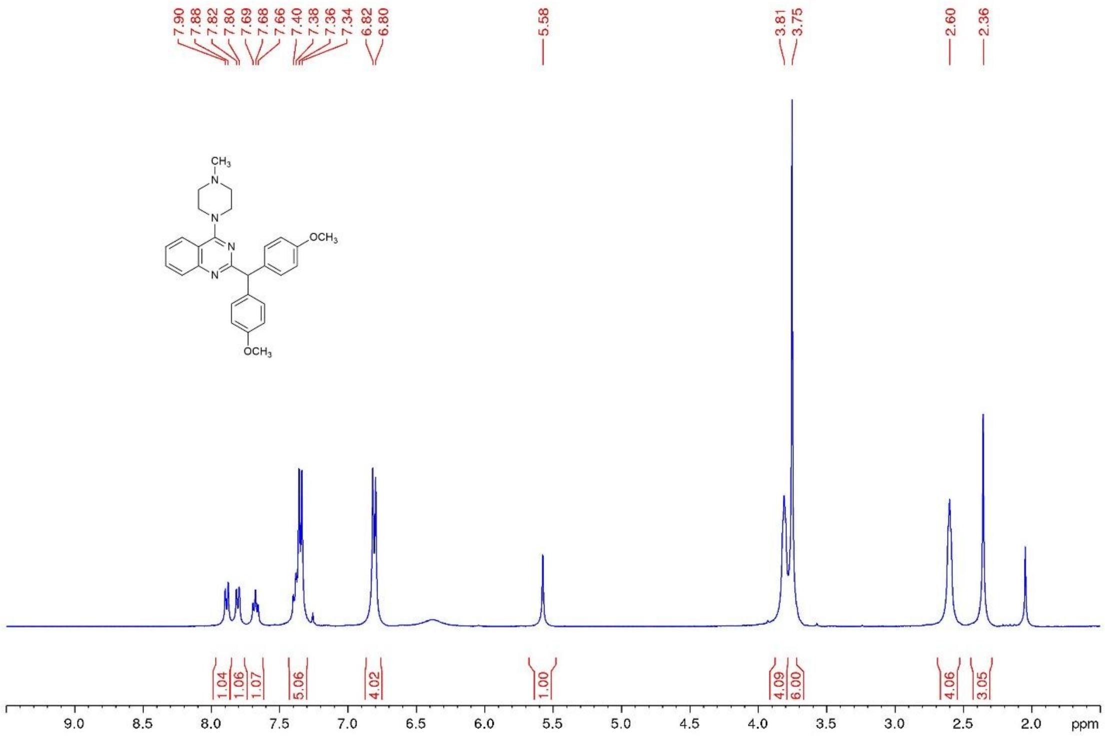

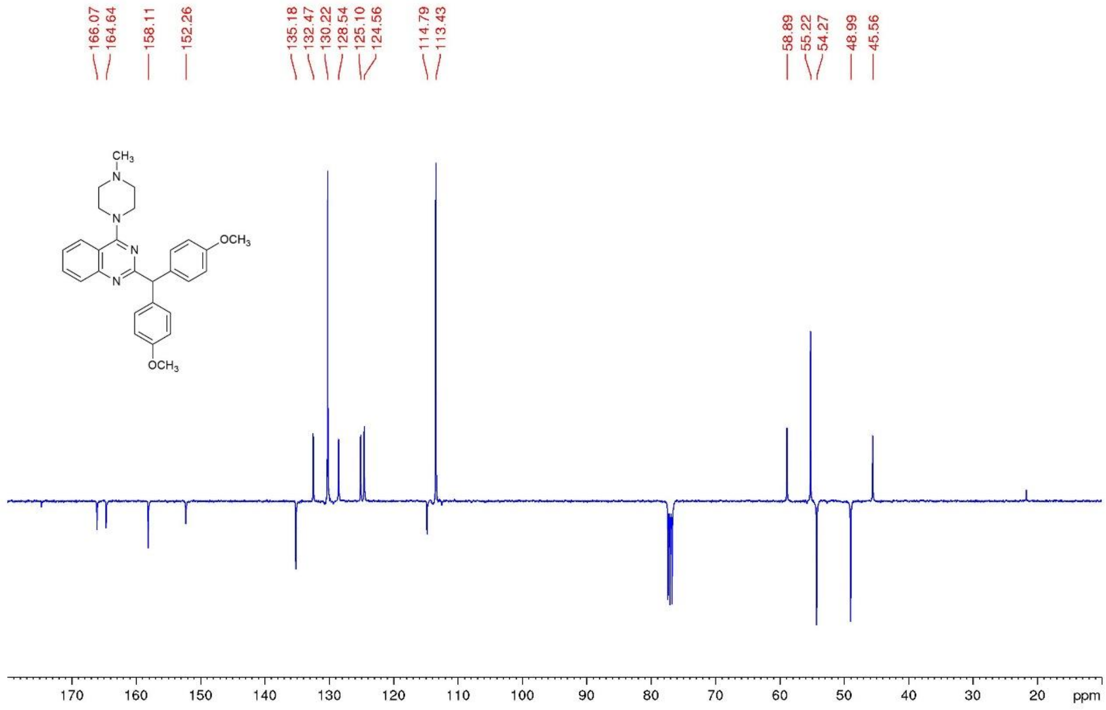

^{1}H-NMR and ^{13}C-APT-NMR spectra of compound **7e**

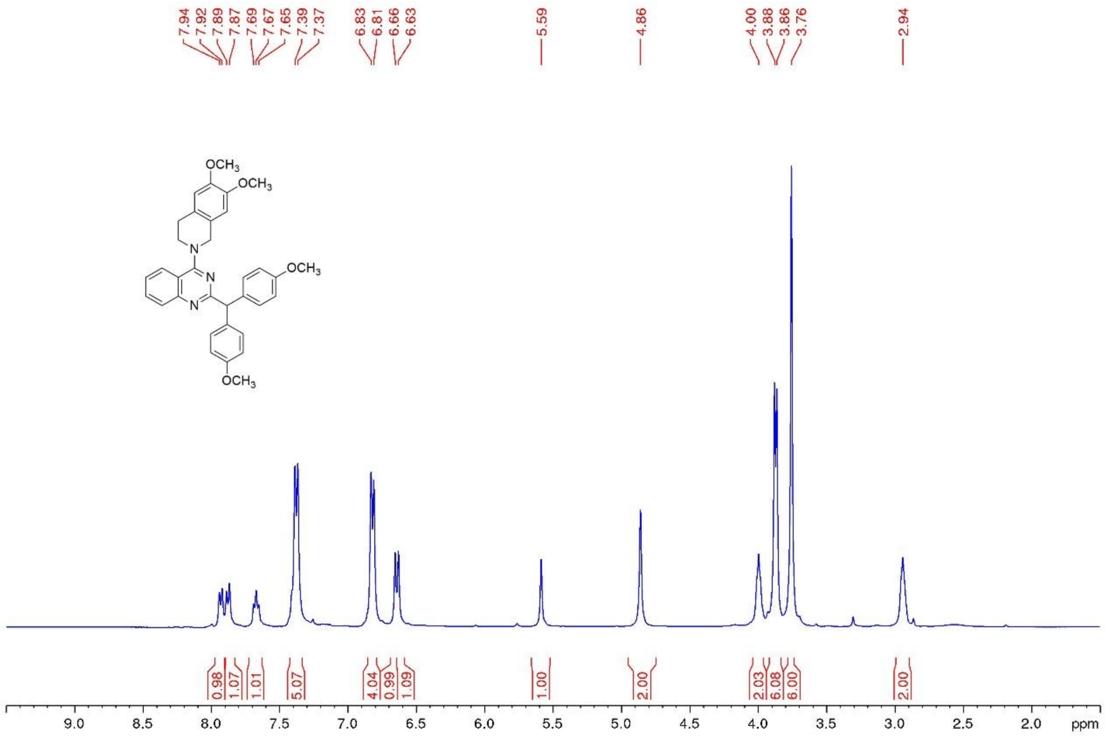

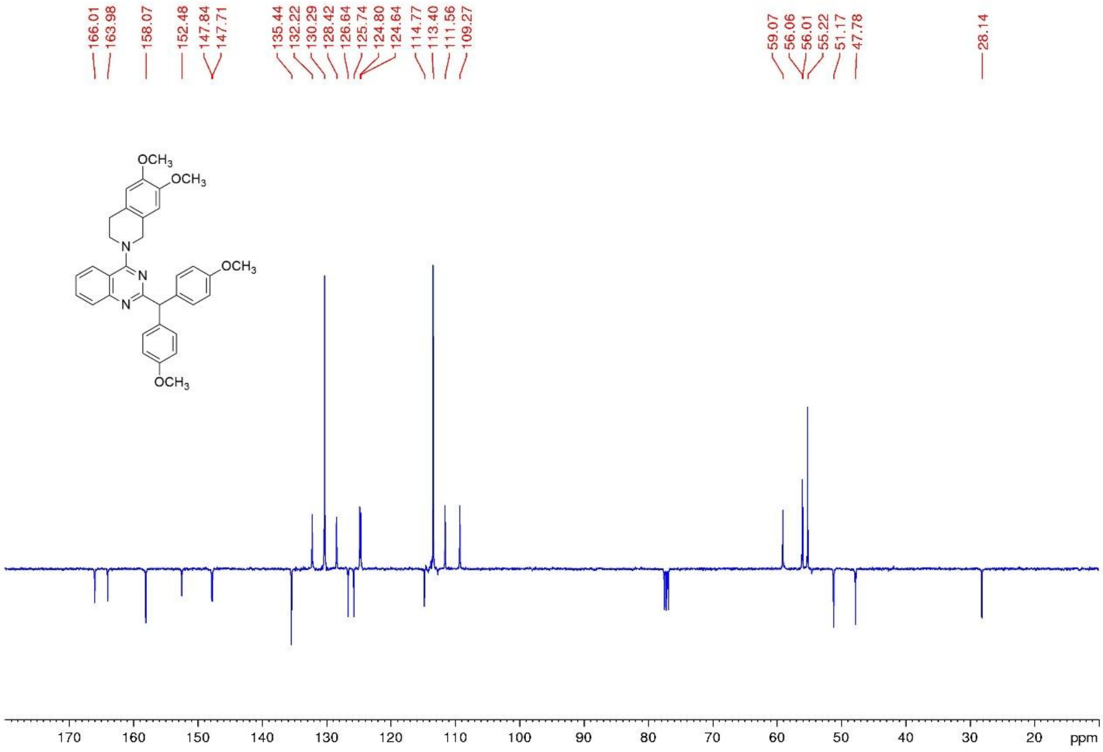

Supplement: Supplementary file 1 — Supporting Information [file CMDC-17-0-s001.pdf]
